# Novel Triazole-Carbohydrazide hydrazones with dual antioxidant and antibacterial potential

**DOI:** 10.1038/s41598-025-26016-x

**Published:** 2025-11-21

**Authors:** Safa A. Badawy, Nagwan M. Rewish, Ahmed A. Fadda, Mohamed R. Elmorsy

**Affiliations:** Department of Chemistry, Faculty of Science, Mansoura University, El- Gomhoria Street, Mansoura, 35516 Egypt

**Keywords:** Hydrazide-hydrazones, Antioxidants, Antibacterial, Molecular docking, DFT, Chemical biology, Computational biology and bioinformatics, Chemistry

## Abstract

In this study, a novel series of triazole-based carbohydrazide hydrazones (**NM-1** to **NM-11**) was synthesized and structurally characterized using spectroscopic methods including FT-IR, ^1^H NMR, ^13^C NMR, and mass spectrometry. The antioxidant activity of the synthesized compounds was assessed via the DPPH assay, revealing that compounds **NM-7** and **NM-10** exhibited potent radical scavenging capacity with IC_50_ values of **0.04 ± 0.05** and **0.06 ± 0.07** mg/mL, respectively, approaching that of ascorbic acid. Antibacterial evaluation against Gram-positive (*Staphylococcus aureus*,* Bacillus subtilis*) and Gram-negative (*Klebsiella pneumoniae*,* Enterobacter cloacae*) strains demonstrated that compounds **5**, **NM-7**,** NM-8**, and **NM-10** had significant inhibitory effects, particularly against Gram-positive bacteria. Molecular docking studies targeting *E. coli* DNA gyrase B (PDB ID: 6YD9) corroborated the biological findings, with compound **5** showing the highest binding affinity (-7.47 kcal/mol). Furthermore, density functional theory (DFT) calculations provided insights into electronic structures and reactivity descriptors, supporting the stability and bioactivity of selected derivatives. These findings collectively highlight the dual therapeutic and functional potential of these carbohydrazide-based molecules as promising candidates for further development in antimicrobial and antioxidant applications.

**Supplementary Information:**

The online version contains supplementary material available at 10.1038/s41598-025-26016-x.

## Introduction

**Fig. 1:**
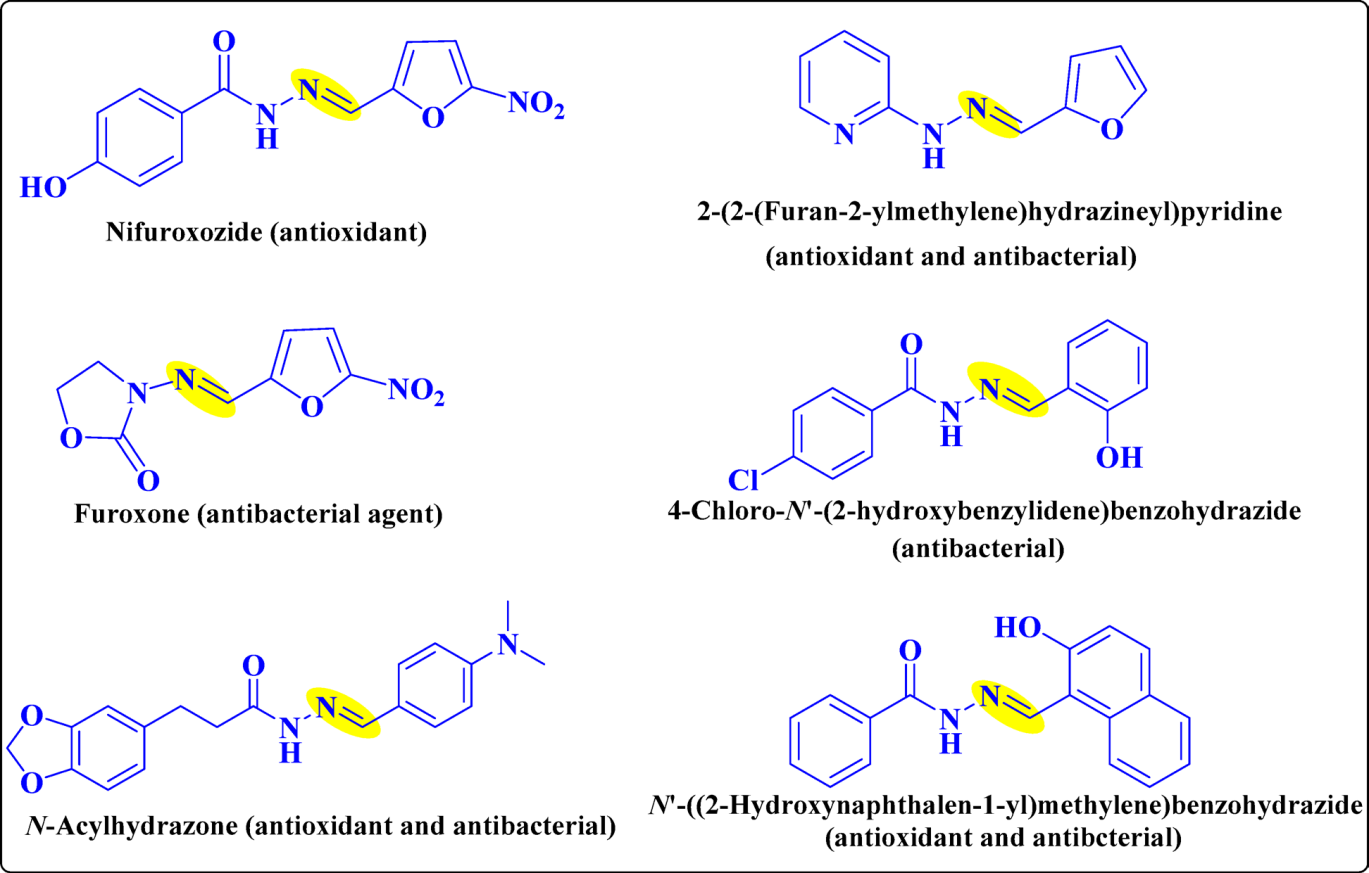
An activity of carbohydrazide in biology.

 Hydrazones are versatile organic compounds with broad structural and biological diversity, exhibiting activities such as anti-inflammatory, analgesic, anticonvulsant, antioxidant, anticancer, anti-HIV, and antibacterial effects, making them valuable in medicinal chemistry^1^. They are also applied in heterocyclic synthesis, metal coordination, and organocatalysis. Compared to imines, hydrazones offer greater hydrolytic stability, easier preparation, and improved crystallinity. Their dual reactivity arises from two nitrogen atoms of distinct nucleophilicity and a C = N bond, enabling flexible synthetic and biological applications^2^. Hydrazide–hydrazone derivatives, typically synthesized by condensing hydrazides with aldehydes or ketones in alcoholic solvents, are routinely characterized by diagnostic IR (C = *N* ~ 2215 cm⁻¹, C = O ~ 1650 cm⁻¹, N–H ~ 3300 cm⁻¹), ¹H NMR (imine δ 7–8 ppm, N–H δ 10–12 ppm), and ¹³C NMR (carbonyl δ 160–170 ppm) data^3^. Over the past decade, many such derivatives have shown promising antibacterial properties, an important feature in the context of multidrug resistance^4^. Derivatives bearing azetidin-2-one and pyrimidine motifs demonstrated strong activity against Gram-positive bacteria (e.g., *Staphylococcus aureus*, *Bacillus subtilis*) and moderate activity against Gram-negative strains (*Escherichia coli*, *Klebsiella pneumoniae*, *Enterobacter cloacae*, *Salmonella typhi*). Antifungal potential was also noted against *Aspergillus niger*, *Fusarium oxysporum*, and *Trichoderma viride*. SAR investigations highlight that halogen substitution on phenyl rings enhances antibacterial potency, underlining the role of structural modifications in optimizing activity^5^. Such findings support the potential of hydrazones as scaffolds for the development of novel antimicrobial agents, though further studies are required to clarify their precise mechanisms of action. As shown in Fig. 1, acyl hydrazones, in particular, represent an especially valuable subclass due to their diverse pharmacological activities, including antibacterial, antifungal, antineoplastic, antiviral, antioxidant, anti-inflammatory, analgesic, immunomodulatory, antidiabetic, anticonvulsant, and cardiovascular-modulating properties^6^. Their clinical relevance is underscored by approved drugs such as the antibiotic nitrofurazone, the antitubercular agent isoniazid, and the antidepressant isocarboxazid, all of which contain a hydrazide–hydrazone moiety^7^. More recently, evidence has shown that hydrazide–hydrazone derivatives also possess significant antioxidant properties. Because oxidative stress is implicated in numerous chronic diseases, antioxidant activity represents a critical pharmacological parameter. The radical scavenging activity of these compounds is commonly assessed using the stable radical 2,2-diphenyl-1-picrylhydrazyl (DPPH). Upon interaction with antioxidants, the deep violet DPPH solution undergoes decolorization, enabling spectrophotometric quantification^8,9^. The Brand-Williams method remains a standard approach, typically involving incubation of test compounds (31–250 µM) with a methanolic DPPH solution (1 mmol/L) for 30 min in the dark. Trolox is often employed as a reference antioxidant, and inhibition is calculated based on absorbance changes between control and sample solutions^10,11^. Complementary to experimental assays, computational approaches such as density functional theory (DFT) provide valuable insights into the electronic structure and reactivity of hydrazones. Energy-minimized conformations, global reactivity descriptors (e.g., hardness, electrophilicity index), and frontier molecular orbital (FMO) analyses reveal electronic distributions and reactive centers, supporting rational predictions of biological activity. These calculations aid in establishing reliable structure–property and structure–activity correlations, thus guiding the design of more effective derivatives. In this study, **NM-1** to **NM-11** derivatives were synthesized via condensation, cyclization, and Michael addition reactions, with structures confirmed by spectroscopic analyses and synthetic schemes. Density Functional Theory (DFT) is based on electron density as the fundamental variable rather than the many-body wave function. Its theoretical foundation lies in the Hohenberg–Kohn theorems^13^, which state that all ground-state properties of a many-electron system are uniquely determined by its electron density. Owing to its balance between computational efficiency and accuracy, DFT has become a widely used approach for predicting and analyzing molecular geometries, electronic distributions, reactivity descriptors, and spectroscopic properties, particularly in large or complex systems^14^. Beyond supporting experimental findings, DFT provides valuable insights into molecular structure, reactivity, and spectroscopy, enabling the prediction of properties and mechanisms that are often inaccessible to direct experimental measurement. Biological assays demonstrated that cyanoacryloyl-conjugated derivatives displayed the strongest antioxidant and antibacterial activities, underscoring their potential as multifunctional pharmacophores. Furthermore, molecular docking with *E. coli* DNA gyrase B (PDB ID: 6YD9) was performed to correlate binding interactions with antibacterial efficacy, with emphasis on cyanoacetyl, cyanoacryloyl, nitro, and chloro substituents as key contributors to ligand affinity and activity against Gram-positive strains.

## Results and discussions

### Synthesis of carbohydrazide-based compounds NM-1–11

**Fig. 2:**
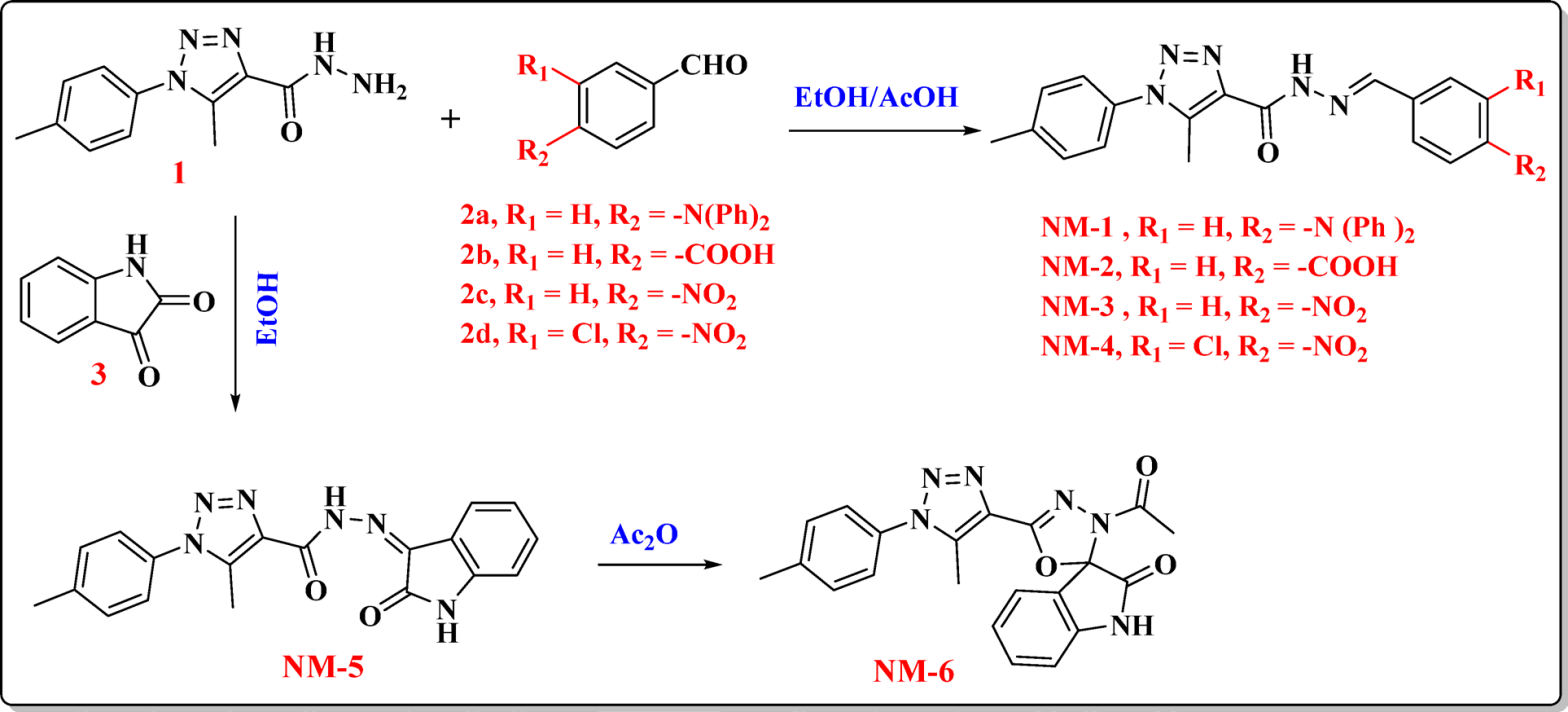
Synthesis of carbohydrazide compounds (**NM-1–6**).

The synthetic pathway commenced with 5-methyl-1-(*p*-tolyl)−1*H*−1,2,3-triazole-4-carbohydrazide as a synthetically valuable precursor for the construction of diverse heterocyclic derivatives. The hydrazide moiety (-CONHNH_2_) exhibits high reactivity toward carbonyl-containing compounds, enabling the formation of Schiff bases (hydrazones) through a well-established condensation mechanism^15^. This process involves the nucleophilic attack of the terminal –NH_2_ group on the electrophilic carbonyl carbon of aldehydes (**2a-2d**) or ketones (**3**), leading to a carbinolamine intermediate, which subsequently undergoes dehydration to furnish the corresponding hydrazone derivatives **NM-1** to **NM-5**. Specifically, condensation with 4-(diphenylamino) benzaldehyde yielded **NM-1**, while reactions with 4-formylbenzoic acid, 4-nitrobenzaldehyde, 3-chloro-4-nitrobenzaldehyde, and isatin produced **NM-2**, **NM-3**, **NM-4**, and **NM-5**, respectively. Notably, the isatin-derived hydrazone (**NM-5**) underwent intramolecular cyclization upon treatment with acetic anhydride, affording the spiro[indoline-oxadiazolone] framework **NM-6**. The proposed mechanism involves an initial acetylation step, followed by intramolecular nucleophilic attack and cyclization, resulting in the formation of a fused^1,3,4^oxadiazole ring system Fig. 2. The chemical structures of **NM-1–6** in were validated through elemental and spectroscopic analyses. The infrared spectra of the **NM-1** compounds revealed the characteristic vibrational bands corresponding to the N-H, =CH, and C = O functional groups at 3314, 3060, and 1686 cm⁻¹, respectively. The ^1^H NMR spectroscopy provided further substantiation. The protons of (2CH_3_) exhibited two singlet signals at *δ* 2.41 and 2.49 ppm. The imine (CH = N) and amine (N-H) protons exhibited singlet signals at *δ* 8.00 and 12.01 ppm, respectively. The ^13^C NMR spectra identified the carbon atoms of the two methyl groups (2CH_3_) and the carbonyl group at *δ* 9.4, 20.7, and 157.0 ppm. In the case of **NM-2**, the proton associated with the carboxylic acid (COOH) group was discerned as the origin of a downfield singlet observed at *δ* 13.09 ppm. The composition of the molecules was subsequently confirmed by ^13^C NMR spectroscopy. A unique signal, indicative of the carbonyl carbon in the carboxylic acid group, was observed at *δ* 166.9 ppm. Compound **NM-2** was confirmed at the molecular level using mass spectrometry, revealing a molecular ion peak at *m/z* = 363 (24.45%), consistent with the chemical formula C_19_H_17_N_5_O_3_. The IR spectrum of **NM-3** indicated the presence of suitable functional groups, exhibiting absorption bands at 3316 cm⁻¹ (N–H), 3040 cm^− 1^ (= C-H), and 1681 cm⁻¹ (C = O). The molecular ion peak at *m/z* = 364 (14.48%) in the mass spectra of **NM-3** corresponded to the calculated chemical formula C_18_H_16_N_6_O_3_. The infrared spectra of **NM-4** exhibited notable absorption bands at 3298 cm⁻¹ (N–H) and 1642 cm⁻¹ (C = O). The imine proton (CH = N) and the amine proton (N-H) are responsible for the single peaks at *δ* 9.93 ppm and *δ* 11.93 ppm, respectively, in the **NM-4** ^1^H NMR spectrum. The hydrazone counterpart, referred to as **NM-5**, was synthesized through the condensation of hydrazide **(1)** and indoline-2,3-dione in acetic acid. As illustrated in **(**Fig. 2**)**, the spirocyclic compound **NM-6** was synthesized through the cyclization of **NM-5** with acetic anhydride under elevated temperature conditions^16^. The infrared (IR) spectroscopy inspection of **NM-5** unveiled notable absorption bands at 1702 and 1671 cm^− 1^, indicative of the presence of two carbonyl groups, alongside a band at 3232 cm⁻¹ associated with the -NH group that is functional. The proton associated with the -NH group was discerned as the origin of a singlet signal at *δ* 11.32 ppm in the ^1^H NMR spectra of **NM-5**. The ^13^C NMR spectra showed signals at *δ* 157.2 and 162.4 ppm, which confirm that there are two carbonyl groups present. Furthermore, the amino (–NH) protons were discerned as a singlet at *δ* 11.34 ppm in the ¹H NMR spectrum of **NM-6**. The spirocyclic structure has two carbonyl carbons, shown by signals at *δ* 163.1 and 172.5 ppm in the ^13^C NMR spectra of **NM-6**. The mass spectrometry analysis showed a peak at *m/z* = 402 (12.42%), which matched the chemical formula C_21_H_18_N_6_O_3_.

### Synthesis of new carbohydrazide compounds NM-7 to NM-11

**Fig. 3:**
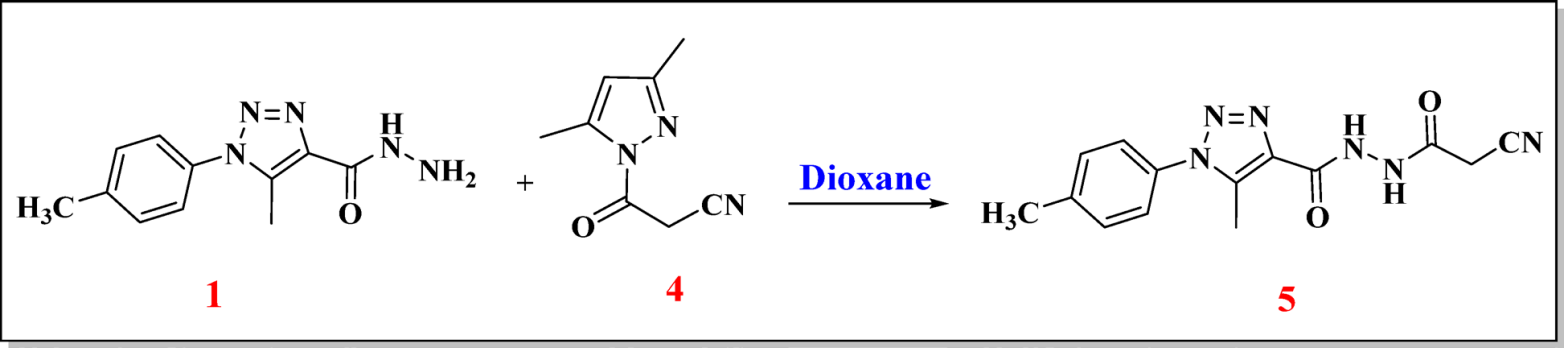
Synthesis of N’-(2-cyanoacetyl)−5-methyl-1-(p-tolyl)−1 H-1,2,3-triazole-4-carbohydrazide (5).

**Fig. 4:**
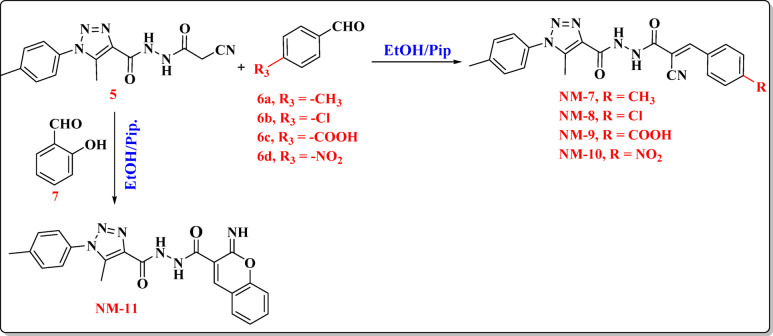
Synthesis of carbohydrazide-based dyes (**NM-7–11)**.

Figures 3 and 4 outline the stepwise synthesis of the carbohydrazide-based compounds **NM-7** to **NM-11**. The process began with the preparation of the key intermediate, *N*’-(2-cyanoacetyl)−5-methyl-1-(*p*-tolyl)−1*H*−1,2,3-triazole-4-carbohydrazide (**compound 5**). This compound was obtained by reacting 5-methyl-1-(*p*-tolyl)−1*H*−1,2,3-triazole-4-carbohydrazide (**compound 1**) with 3,5-dimethyl-1-cyanoacetylpyrazole (**compound 4**) in dioxane solvent. This transformation proceeds through nucleophilic attack by the hydrazide’s terminal –NH_2_ group on the electrophilic carbonyl carbon of the β-ketonitrile, forming a stable acylhydrazide bearing both cyano and pyrazolyl functionalities^17,18^.

As revealed in **Figure (4)**, This intermediate serves as a central platform for the synthesis of a series of derivatives (**NM-7** to **NM-11**) via condensation and Michael-type addition reactions with various aldehydes (**6a-6d**) and chromene (**7**) carbonyl derivatives. The activated methylene group, flanked by electron-withdrawing cyano and carbonyl groups, facilitates these transformations, enabling the formation of C = C bonds within extended conjugated systems, which contribute to product stability through resonance. The condensation reactions proceed via a well-defined mechanism. Initially, the activated methylene group, positioned α to the cyano functionality, undergoes nucleophilic addition to the electrophilic carbonyl carbon of the aldehyde or chromene derivative. This step leads to the formation of a carbinol intermediate, which subsequently undergoes acid- or base-catalyzed dehydration, yielding the final α, β-unsaturated hydrazide or iminochromene derivative, depending on the substrate. The presence of electron-withdrawing substituents on the aromatic aldehyde or chromene scaffold enhances the electrophilicity of the carbonyl carbon, thereby facilitating the condensation and improving overall reaction efficiency. A series of hydrazide derivatives (**NM-7** to **NM-11**) were synthesized via condensation of the acylhydrazide intermediate with various aromatic aldehydes and a chromene-based carbonyl compound. The electronic nature and substitution pattern of the electrophilic carbonyl substrates significantly influenced the structural features and potential reactivity of the resulting products. **NM-7**, **NM-8**, **NM-9**, **NM-10** Formed via condensation with *p*-tolylaldehyde (**6a**), 4-chlorobenzaldehyde (**6b**), 4-formylbenzoic acid (**6c**) and 4-nitrobenzaldehyde (**6d**), respectively yielding an α, β-unsaturated cyan-acryloyl hydrazide incorporating a *p*-tolyl (electron-donating) substituent. **NM-11**, Synthesized via condensation with a 2*H*-chromene-3-carbonyl derivative, affording a *N*’-(2-imino-2*H*-chromene-3-carbonyl) hydrazide Fig. 4. The chemical structures of the produced dyes have been validated through the application of infrared (IR) spectroscopy, ^1^H NMR, ^13^C NMR, and mass spectrometry (MS), among other methods involving spectroscopy. A series of spectroscopic analyses has been conducted to confirm the structure of **NM-7**. The infrared spectra revealed characteristic absorption bands associated with the N–H stretching vibration at 3300 cm⁻¹, cyano group (C ≡ N) at 2215 cm⁻¹, and the carbonyl group (C = O) at 1676 cm⁻¹. The singlet signals observed at δ 2.35, 2.41, and 2.49 ppm in the ¹H NMR spectrum can be attributed to the three methyl protons. The existence of the three-methyl carbon was substantiated by the relevant signals observed at *δ* 9.7, 21.2, and 21.5 ppm in the ^13^C NMR spectrum. In accordance with the chemical formula C_22_H_20_N_6_O_2_, the mass spectrum of **NM-7** revealed a molecular ion peak at *m/z* = 400 (15.18%). The infrared spectra of the compound **NM-8** exhibited absorption bands at 3344 cm⁻¹ (N-H), 2217 cm⁻¹ (C ≡ N), and 1652 cm⁻¹ (C = O), thereby suggesting that there were plenty of significant functional groups that lie within the molecule. The structural recognition of the compound **(NM-9)** was substantiated through the application of spectroscopic methods. The infrared spectrum revealed absorption bands analogous to the N–H (3322 cm⁻¹), cyano (C ≡ N, 2217 cm⁻¹), and carbonyl (C = O, 1659 cm⁻¹) functional groups. The ^1^H NMR spectrum demonstrated singlet signals for the vinylic (CH = C) and amine (N-H) protons at *δ* 7.92 and 10.76 ppm, respectively. A distinct signal for the carbonyl carbon was observed at *δ* 159.80 ppm in the ^13^C NMR spectrum. Furthermore, a molecular ion peak at *m/z* = 430 (10.75%) was seen in the mass spectrum, aligning with the chemical formula C_21_H_17_ClN_6_O_2_. The infrared spectra of dye **NM-10** showcased absorption bands at 3325 cm⁻¹ (N–H), 2925 cm⁻¹ (C–H) aliphatic, 2216 cm⁻¹ (C ≡ N), and 1648 cm⁻¹ (C = O), thereby affirming that there are significant functional groups. The singlet signals detected at *δ* 8.00 ppm and *δ* 10.78 ppm in the ^1^H NMR spectrum have been attributed to the vinylic (CH = C) and amine (N-H) protons, respectively. A notable resonance at *δ* 159.62 ppm, indicative of the carbonyl carbon, was observed in the ^13^C NMR spectrum. Consistent with the chemical formula C₂₂H₁₈N₆O₄, a molecular ion peak became apparent via mass spectrometry at *m/z* = 431 (24.72%). Likewise, the coumarin derivative **NM-11** has been created through the cyclo-condensation of compound **(5)** with salicylaldehyde in boiling ethanol in the presence of a catalytic amount of piperidine^19^. The presence of the two carbonyl groups within the coumarin ring system accounts for the absorption bands observed in the **NM-11** IR spectrum, specifically at 3138 cm⁻¹ (N-H) and at 1679 and 1658 cm⁻¹ (2 C = O). In the ^13^C NMR spectrum, two distinct carbonyl signals have been noticed at *δ* 160.07 and 157.3 ppm. The mass spectrum supported the proposed chemical formula C_21_H_18_N_6_O_3_ by showing a molecular ion peak at *m/z* = 402 (22.61%).

## DFT-Based analysis of frontier molecular orbitals and quantum descriptors of carbohydrazide derivatives (NM-1 to NM-11) in relation to their biological activity

The structures of carbohydrazide derivatives **NM-1** to **NM-11** were generated utilizing Gauss View 6.0 in conjunction with the Gaussian 09 W software package^20^. The HOMO-LUMO energy levels values and the associated band gap (ΔE) were computed to evaluate the compounds’ chemical reactivity, stability, biological potential, and quantum chemical properties are shown in Table 1. The calculated and analyzed frontier molecular orbitals comprise global chemical hardness (*η* = ½ (E_LUMO_ − E_HOMO_), global softness (*σ* = 1/2*η*), nucleophilicity index (*Nu* = 1/*ω*), electronegativity (*χ* = − ½ (E_HOMO_ + E_LUMO_)), and global electrophilicity index (ω = µ²/2*η*)^21^. When a chemical has an elevated antioxidant activity, it is frequently connected with a reduced band gap energy between its HOMO and LUMO^22^. The orbitals of the HOMO and LUMO molecules were utilized to assess the distribution of their wavefunctions and their relative reactivity. Table 1 shows that changing the substituents affected the band gap energies and reactivity of the compounds from **NM-1** to **NM-11**^23^. To better understand the relationship between molecular structure, electronic properties, and biological activity, quantum chemical descriptors for compounds **NM-1** to **NM-11** were calculated using DFT and are summarized in Table 1. The results indicate that compounds **NM-2**,** NM-3**,** NM-4**,** NM-5**, and **NM-10** exhibited the lowest HOMO–LUMO energy gaps (1.31–1.86 eV), reflecting enhanced electronic reactivity and potential for (ICT), primarily due to the presence of conjugated aromatic systems and electron-withdrawing groups such as nitro and carboxylic acid moieties. Notably, **NM-2**, containing a diphenylamino substituent, showed the lowest Eg and highest electrophilicity index (ω = 12.00 eV), correlating with its strong antioxidant and antibacterial activity. In contrast, **NM-6** exhibited the highest energy gap (4.59 eV), greatest hardness (η = 2.29 eV), and lowest softness (σ = 0.87 eV^− 1^), indicating low chemical reactivity, which aligns with its weak biological performance. Compounds with high electrophilicity (**NM-3** and **NM-4**) demonstrated strong antibacterial activity, while those with moderate nucleophilicity (**NM-7 and NM-8**) exhibited effective radical scavenging potential, likely due to their capacity to donate electrons. **NM-1** showed the highest nucleophilicity index (0.20 eV), yet its moderate biological performance suggests that optimal activity depends not only on nucleophilicity but also on balanced electronic characteristics and functional group distribution. Overall, the compounds that displayed the most favorable biological activity **(NM-2**,** NM-4**,** NM-8**,** and NM-10**) shared structural features such as extended π-conjugation, ICT-facilitating groups, and donor–acceptor functionalities, supporting the conclusion that specific electronic descriptors particularly low Eg, high ω, and balanced *Nu* are predictive of enhanced antioxidant and antibacterial properties in this class of carbohydrazide-based hydrazones.

**Table 1:** The produced substances’ quantum chemical descriptors for compounds **NM-1–11.**

Compounds	E_HOMO_ _(eV)_	E_LUMO (eV)_	E_g_ _(eV)_	Χ _(eV)_	µ _(eV)_	η _(eV)_	σ _(eV_^−1^_)_	ω _(eV)_	Nu _(eV)_
NM-1	−4.20	−2.17	2.03	3.18	−3.18	1.01	0.49	5.00	0.20
NM-2	−4.61	−3.30	1.31	3.95	−3.95	0.65	3.07	12.00	0.08
NM-3	−5.09	−3.24	1.85	4.16	−4.16	0.92	2.17	9.40	0.10
NM-4	−5.12	−3.35	1.77	4.23	−4.23	0.88	2.27	10.16	0.09
NM-5	−5.02	−3.16	1.86	4.09	−4.09	0.93	2.15	8.99	0.11
NM-6	−6.38	−1.79	4.59	4.08	−4.08	2.29	0.87	3.63	0.27
NM-7	−6.07	−2.54	3.53	4.30	−4.30	1.76	1.13	5.25	0.19
NM-8	−6.19	−2.58	3.61	4.38	−4.38	1.80	1.11	5.32	0.18
NM-9	−6.44	−3.23	3.21	4.83	−4.83	1.60	1.25	7.29	0.13
NM-10	−5.66	−3.88	1.78	3.72	−3.72	0.89	2.24	7.77	0.12
NM-11	−5.65	−2.08	3.57	3.86	−3.86	1.78	1.12	4.18	0.23

**Fig. 5:**
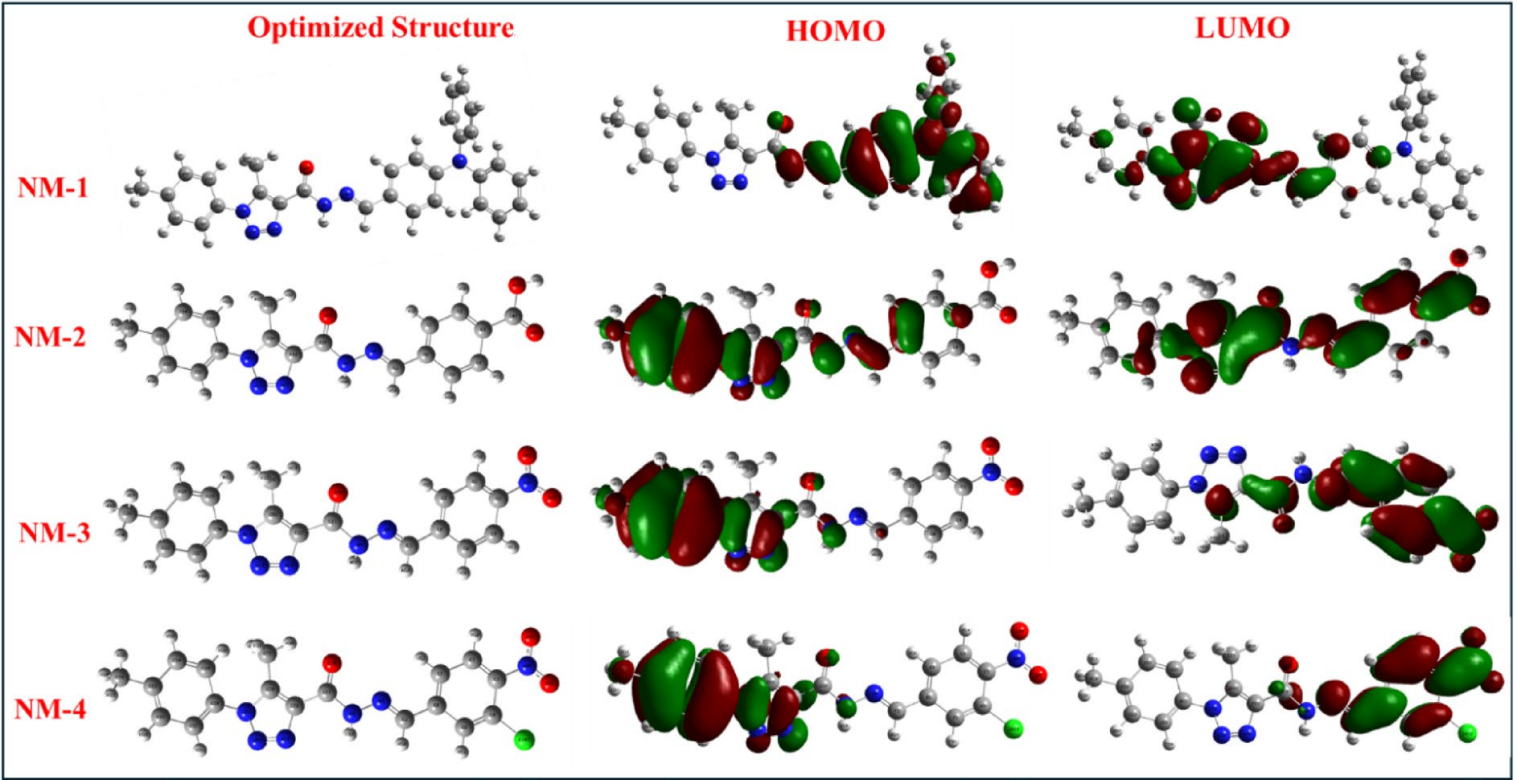
HOMOs and LUMOs geometries for compounds NM-1–4.

**Fig. 6:**
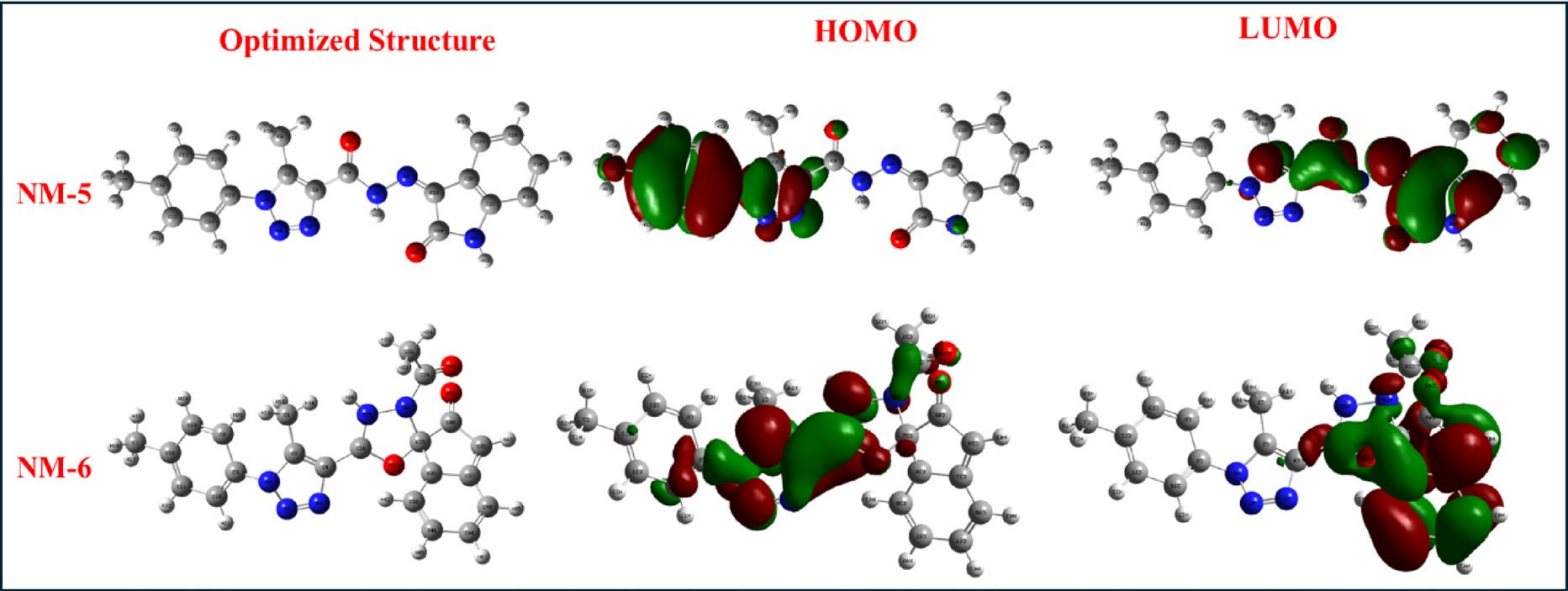
HOMOs and LUMOs geometries for compounds NM-5 and NM-6.

**Fig. 7:**
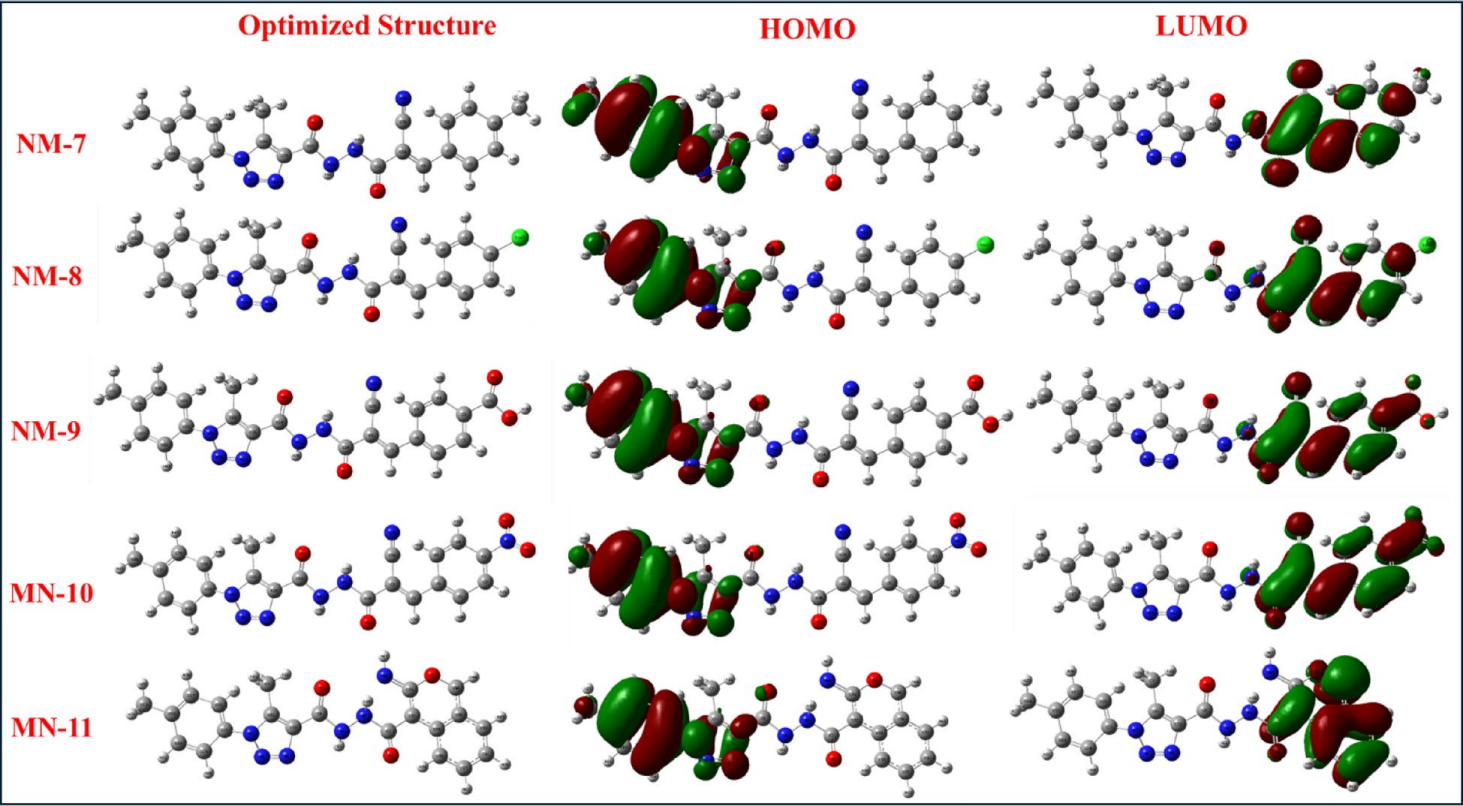
HOMOs and LUMOs geometries for compounds NM-7–11.

FMO analysis enhances comprehending the intramolecular charge transfer (ICT) and optoelectronic features found in molecules by evaluating the electronic charge transition between HOMO and LUMO^24,25^. The HOMO and LUMO correspond to the valence and conduction bands, respectively, as per band theory^26^. The conductive properties of the electronic charge density and the photon characteristics of **NM1-4** have been examined^27,28^. The HOMO and LUMO geometries of compounds **NM-1 to NM-11** provide crucial insights into their electronic distribution, reactivity, and bioactivity. As illustrated in Figs. 5, 6 and 7, the spatial distribution of frontier molecular orbitals (FMOs) varies significantly across the series, reflecting the nature of the substituent groups and the degree of π-conjugation in each compound. **NM-1**, featuring a strong electron-donating diphenylamino group, displays a delocalized HOMO spread across the donor moiety and the conjugated triazole-carbohydrazide core. The LUMO is localized predominantly on the triazole ring and the hydrazone linkage, indicating potential for intramolecular charge transfer (ICT) upon excitation. The resulting HOMO-LUMO separation correlates with its relatively wide band gap (2.03 eV), suggesting chemical stability and moderate reactivity. In contrast, **NM-2**, which bears a carboxylic acid group, exhibits HOMO and LUMO distributions localized over the π-conjugated system and the COOH group. The electron-withdrawing nature of the COOH moiety stabilizes the LUMO, narrowing the band gap (1.31 eV), and enhancing reactivity. Similarly, **NM-3** and **NM-4**, which contain nitro and chloro-nitro substituents respectively, show strong localization of the LUMO on the electron-withdrawing groups, resulting in deep HOMO levels and further narrowed band gaps (1.85 and 1.77 eV, respectively). These localized LUMO geometries support their observed potential as electrophilic agents and their ability to accept electrons in redox environments.

**NM-5** and **NM-6**, with spirocyclic frameworks, demonstrate distinct electronic configurations. **NM-5’s** HOMO is delocalized across the isatin-triazole core, with the LUMO positioned on the hydrazone linkage and carbonyl groups, consistent with moderate reactivity (Eg = 1.86 eV). In contrast, **NM-6** reveals a highly stabilized HOMO (−6.38 eV) and a shallow LUMO (−1.79 eV), attributed to poor orbital overlap due to its orthogonal spiro[indoline-oxadiazole] structure. The resulting wide band gap (4.59 eV) reflects low reactivity, making **NM-6** an excellent radical scavenger due to its high nucleophilicity and low electrophilicity.

For **NM-7** to **NM-11**, a variety of electron-donating and electron-withdrawing substitutions result in diverse FMO distributions. **NM-8** and **NM-9** show LUMO densities primarily on the cyanoacryloyl and benzoic acid groups, while their HOMOs are distributed over the *p*-tolyl and triazole regions, supporting extended conjugation and ICT behavior. **NM-9** benefits from enhanced stabilization of the LUMO by multiple withdrawing groups, resulting in a relatively small band gap (3.21 eV) despite its deep HOMO. **NM-10**, bearing a nitro group, has one of the most stabilized LUMOs (−3.88 eV), with HOMO density delocalized over the triazole-hydrazide core. This dual conjugation and substitution lead to a narrow band gap (1.78 eV), aligning with its notable antioxidant and antibacterial activity. **NM-11**, composed of a chromene-imino-carbonyl framework, shows partial LUMO delocalization across the carbonyl-containing moieties, but with limited overlap, resulting in a relatively large band gap (3.57 eV), suggesting reduced reactivity toward electrophilic species. Collectively, the HOMO-LUMO geometries indicate that molecules with extended conjugation and distinct donor-acceptor character (e.g., **NM-2**,** NM-4**,** NM-10**) show enhanced reactivity and biological activity. Conversely, molecules with interrupted or orthogonal conjugation (e.g., **NM-6**,** NM-11**) exhibit lower chemical reactivity but may be more suitable as electron reservoirs or radical scavengers.

### Frontier molecular orbitals and global reactivity descriptors

 To investigate the relationship between molecular structure and biological activity, Density Functional Theory (DFT) calculations were carried out for the synthesized carbohydrazide-based hydrazones (**NM-1 to NM-11**). Key global reactivity descriptors HOMO and LUMO energies, energy gap (Eg), chemical hardness (η), softness (σ), electrophilicity index (ω), and nucleophilicity index (Nu) are summarized in Table 1^29^. The HOMO–LUMO energy gap (Eg) is a useful indicator of molecular reactivity and kinetic stability: smaller gaps are generally associated with higher electronic reactivity and a greater propensity for intramolecular charge transfer (ICT), which can enhance both antioxidant and antibacterial interactions^30^. Compounds **NM-2**,** NM-3**,** NM-4**,** NM-5**, and **NM-10** exhibited low energy gaps (1.31–1.86 eV), consistent with their high reactivity and ICT potential. Among them, **NM-2** had the lowest Eg (1.31 eV) and the highest electrophilicity (ω = 12.00 eV). Structurally, **NM-2** features a p-tolyl-substituted triazole donor and a benzaldehyde-derived acceptor moiety, which supports strong donor–acceptor interaction and efficient ICT. However, despite this favorable electronic profile, **NM-2** showed poor antioxidant activity (IC_50_ = 3.55 ± 0.96 mg/mL), likely due to its low nucleophilicity (Nu = 0.08 eV). This suggests that excessive electron-accepting character may hinder radical scavenging if electron-donating ability is insufficient. In contrast, **NM-10**, with a similarly low Eg (1.78 eV), moderate electrophilicity (ω = 7.77 eV), and higher nucleophilicity (Nu = 0.12 eV), demonstrated significant antioxidant and antibacterial activity, indicating that a more balanced distribution of electron-donating and accepting functionalities may be favorable for bioactivity. At the opposite end of the spectrum, **NM-6** exhibited the widest Eg (4.59 eV), highest hardness (η), and lowest softness (σ), due to its spirocyclic, non-planar geometry that limits π-conjugation and orbital overlap. These features correlate well with its poor performance in both antioxidant and antibacterial assays, supporting the premise that wide HOMO–LUMO separation, high rigidity, and low softness are detrimental to biological function^31^. Compounds **NM-7**,** NM-8**, and **NM-9** displayed intermediate energy gaps (3.21–3.61 eV). Among these, **NM-8** (Eg = 3.61 eV, ω = 5.32 eV, Nu = 0.18 eV) showed excellent antioxidant activity (IC_50_ = 0.09 ± 0.05 mg/mL) and broad-spectrum antibacterial efficacy. These results suggest that moderate electrophilicity combined with high nucleophilicity and good orbital distribution can support dual-function bioactivity.

### Frontier molecular orbitals and intramolecular charge transfer

The spatial distribution of frontier molecular orbitals (FMOs) provides additional insight into the electronic transitions and charge transfer characteristics that underlie biological activity^32^. Representative HOMO and LUMO for compounds (**NM1-11**) are presented in Figs. 5, 6 and 7. For **NM-1**, the HOMO is distributed across the diphenylamino and triazole–carbohydrazide regions, while the LUMO is localized on the hydrazone linkage. With an Eg of approximately 2.03 eV, this orbital arrangement allows moderate ICT, which correlates with its intermediate antioxidant activity (IC_50_ = 1.16 ± 0.93 mg/mL). In **NM-2**, the HOMO is concentrated in the aromatic donor segments (*p*-tolyl and hydrazone moieties), while the LUMO is delocalized over the triazole and carbonyl regions. This configuration supports efficient ICT. However, the low nucleophilicity (Nu = 0.08 eV) and dominant electron-accepting behavior (high ω) align with its poor antioxidant performance. **NM-3** and **NM-4** display LUMO localization on strongly electron-withdrawing nitro and chloro-nitro substituents, respectively, and HOMO density in the phenyl–triazole systems. These features correspond to high electrophilicity and are consistent with their moderate antibacterial activity, particularly against Gram-positive strains, despite limited antioxidant capability^33,34^. **NM-5** shows good HOMO–LUMO delocalization across its isatin–triazole core, supporting moderate antioxidant activity (IC_50_ = 0.12 ± 0.06 mg/mL). In contrast, **NM-6** exhibits poor orbital overlap due to its orthogonal spirocyclic geometry, which restricts ICT and matches its low biological reactivity. Among **NM-7** to **NM-11**, **NM-8** shows favorable HOMO–LUMO separation, with the HOMO centered on the *p*-tolyl–triazole donor regions and the LUMO on the cyanoacryloyl acceptor. This spatial configuration promotes efficient ICT and corresponds well with its strong dual bioactivity. In contrast, **NM-11**, despite having a relatively high energy gap and poor LUMO delocalization, exhibited strong antioxidant activity^35^ (IC_50_ = 0.12 ± 0.19 mg/mL) but no detectable antibacterial activity. This suggests that while extended orbital conjugation and ICT are important for antimicrobial effects, antioxidant activity may be retained in systems with sufficient nucleophilic character, even in the absence of extensive delocalization.

## Biological activity

### Antioxidant activity

**Fig. 8:**
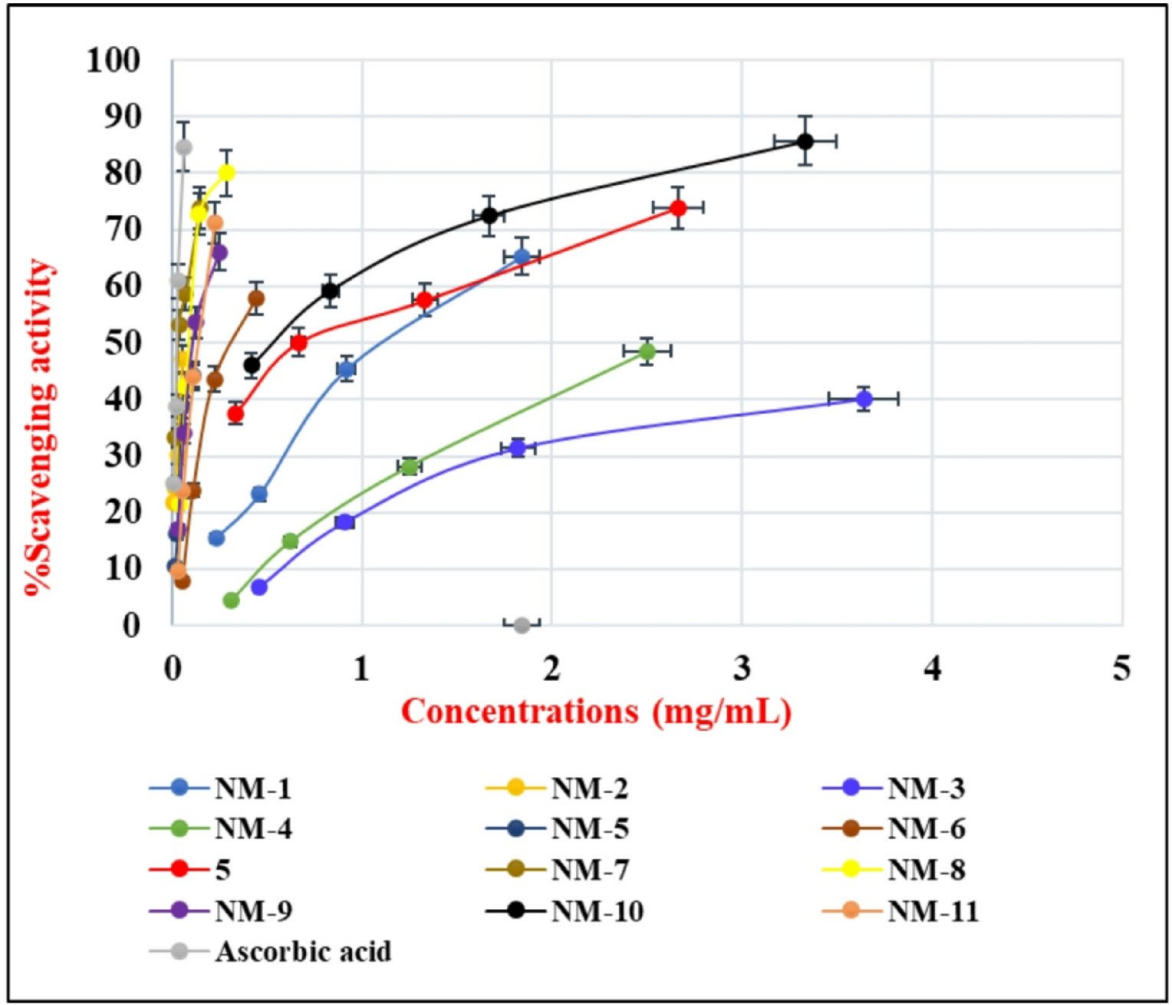
Plotting the Sample Concentrations Against Scavenging Activity Percentage.

**Fig. 9:**
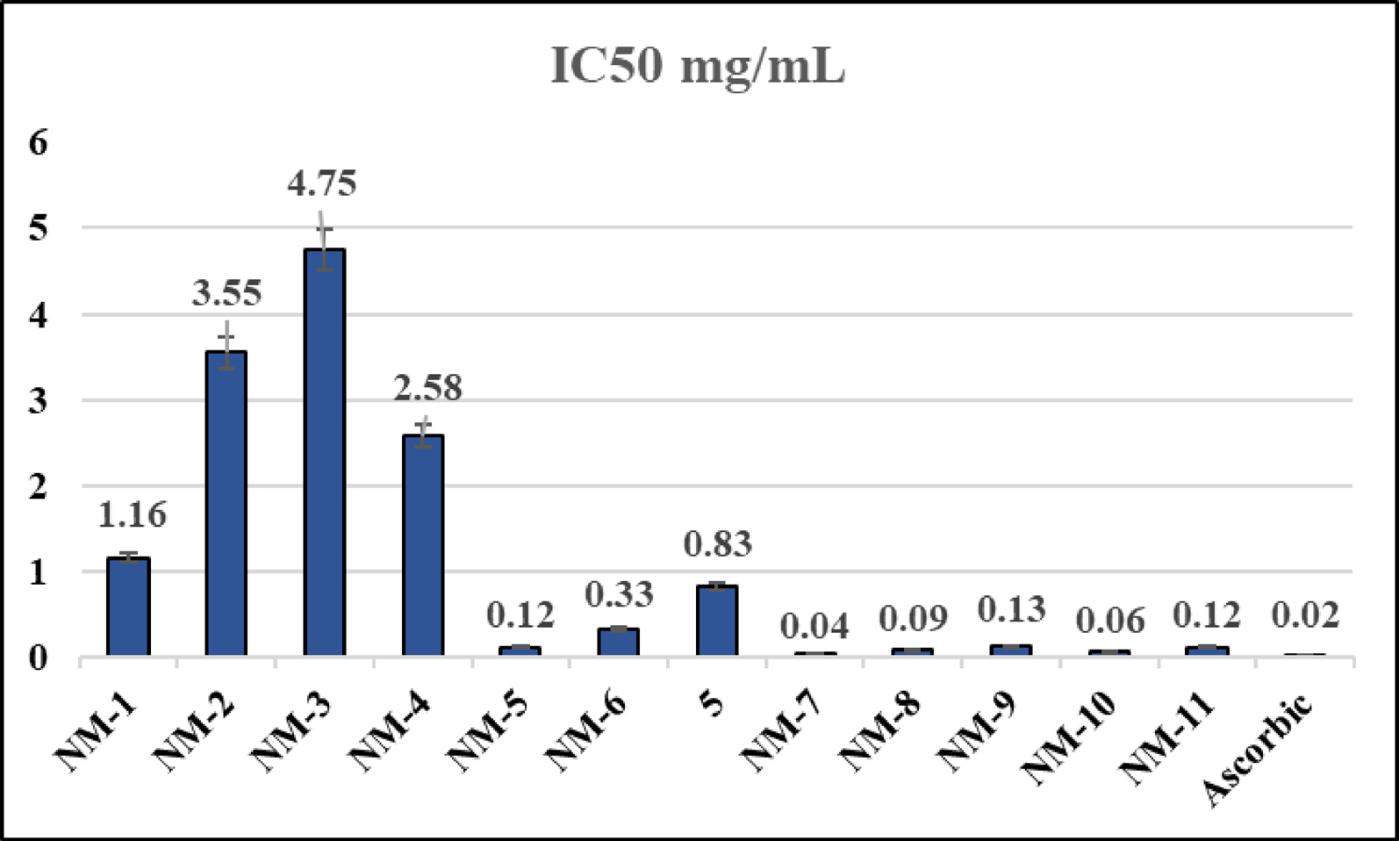
Comparison of the antioxidant results expressed as IC_50_ in mg/mL of the tested samples for NM-1–11 relative to the antioxidant standard.

Further investigation revealed that the newly identified compounds (**NM-1** to **NM-11**) exhibited antioxidant properties. For this aspect of the investigation, each of the compounds was dissolved in DMSO so that their activity could be evaluated under the identical conditions. It is usual practice to use the DPPH (2,2-diphenyl-1-picrylhydrazyl) free radical scavenging assay to evaluate the potential antioxidant properties of just synthesized substances. The absorbance readings from the test samples, compared to the control samples, helped calculate the percentage of antioxidant activity as shown in **Table (S1)**^36,37^. The carbohydrazide derivatives **(NM-1–11)** were evaluated for being able to decolorize 2,2’-diphenyl-1-picrylhydrazyl (DPPH) radical at a concentration of 0.135 mM using a modified DPPH approach in a (DMSO) solution. Ascorbic acid is used as a reference standard. Figures 8 and 9 present a bar chart illustrating the in vitro antioxidant activity data. The various studies were performed in triplicate, and the average results are demonstrated. All synthesized compounds demonstrated a clear dose-dependent DPPH radical scavenging effect, meaning their antioxidant activity increased proportionally with concentration. This behavior is consistent with typical antioxidant mechanisms, where a higher concentration increases the availability of hydrogen or electron donors capable of neutralizing free radicals^38^. Compounds containing electron-donating groups (EDGs) generally exhibited stronger antioxidant activity. EDGs increase electron density, facilitating hydrogen atom transfer (HAT) or single electron transfer (SET) to stabilize free radicals^39^. NM-1, containing a diphenylamino group (a strong EDG), showed moderate antioxidant activity, with 45.42 ± 1.01% scavenging at 0.92 mg/mL and 65.25 ± 0.9% at 1.84 mg/mL. Despite the EDG, its relatively high IC_50_ of 1.16 ± 0.93 mg/mL suggests limited overall efficacy, likely due to structural factors affecting radical stabilization compared to the activity of **NM-8** and **NM-10**. Electron-withdrawing groups (EWGs) such as nitro (–NO_2_) or chloro (–Cl) tend to decrease antioxidant activity by reducing the electron density around key donating sites, making hydrogen/electron transfer less favorable. **NM-3**, bearing a para-nitro group, was among the weakest antioxidants. It achieved only 6.75 ± 0.6% scavenging at 0.455 mg/mL and required 3.64 mg/mL to reach 40.09 ± 1.11%, with an IC_50_ of 4.75 ± 0.88 mg/mL. **NM-4**, containing both nitro and chloro substituents, showed slightly improved but still low activity: 14.83 ± 0.65% at 0.625 mg/mL with an IC_50_ of 2.58 ± 0.46 mg/mL. These results confirm that strong EWGs negatively impact radical scavenging ability, especially when positioned to withdraw electron density from active centers. Compounds **NM-5** and **NM-6** with extended conjugation and resonance-stabilized systems performed significantly better due to improved delocalization of unpaired electrons in the radical form. **NM-5**, featuring an oxoindolin-3-ylidene group, achieved 44.33 ± 0.05% scavenging at just 0.109 mg/mL, with an IC_50_ of 0.12 ± 0.06 mg/mL, indicating strong antioxidant potential due to resonance within its conjugated heterocyclic core. **NM-6**, which includes a spiro-indoline-oxadiazole system, showed moderate activity (57.81 ± 0.08% at 0.444 mg/mL) with an IC_50_ of 0.33 ± 0.13 mg/mL. Its structural rigidity and conjugated scaffold likely aid electron delocalization. Compound **5** contain both EDGs and EWGs, resulting in intermediate antioxidant performance. Compound **5** possesses a cyanoacetyl group (EWG), but the presence of conjugated aromatic rings likely compensates, allowing 73.93 ± 0.11% scavenging at 2.663 mg/mL, decreasing to 37.56 ± 1.01% at 0.333 mg/mL. The IC_50_ of 0.83 ± 0.58 mg/mL suggests moderate efficacy. The strong antioxidant activity of **NM-7** and **NM-8** is due to the presence of key functional groups that stabilize free radicals. The cyanoacryloyl moiety, conjugated with aromatic rings, promotes electron delocalization, while the cyano group enhances electrophilicity for better radical interaction. The aromatic substituents (*p*-tolyl in **NM-7** and 4-chlorophenyl in **NM-8**) aid in resonance stabilization. The 1,2,3-triazole ring and carbohydrazide group contribute through hydrogen bonding and electron donation. In comparison, **NM-7** demonstrated slightly lower maximum scavenging 73.75 ± 0.05% at a much lower concentration of 0.146 mg/mL and exhibited a significantly lower IC_50_ of 0.045 ± 0.05 mg/mL, indicating its higher activity at lower concentration. NM-8 exhibited the highest scavenging activity, achieving 80.07 ± 0.01% inhibition at 0.285 mg/mL with an IC₅₀ of 0.09 ± 0.05 mg/mL. **NM-9** demonstrated moderate antioxidant activity (IC_50_ = 0.13 ± 0.08 mg/mL), likely due to the conjugated system linking cyano, carbonyl, and benzoic acid moieties, which supports radical stabilization despite the presence of some electron-withdrawing functionality. **NM-10**, despite containing a nitro-substituted aryl group (a strong electron-withdrawing group), exhibited high antioxidant activity, with 45.98 ± 0.05% scavenging at 0.417 mg/mL and 85.67 ± 0.08% at 3.333 mg/mL. Its low IC_50_ 0.06 ± 0.07 mg/mL indicates strong radical scavenging capacity. This performance suggests that the compound’s structural features possibly extended conjugation or favorable positioning of functional groups compensate for the presence of the nitro group, allowing efficient electron or hydrogen donation for DPPH radical neutralization. **NM-11**, which incorporates a chromene derivative, showed good antioxidant activity (IC_50_ = 0.12 ± 0.19 mg/mL). The extended conjugation across oxygen and nitrogen atoms contributes to the stabilization of radical intermediates, enhancing its performance. From strongest to weakest antioxidant activity:

**Ascorbic acid** (0.02 ± 0.03) > **NM-7** (0.04 ± 0.05) > **NM-10** (0.06 ± 0.07) > **NM-8** (0.09 ± 0.05) > **NM-5** (0.12 ± 0.06) ≈ **NM-11** (0.12 ± 0.19) > **NM-9** (0.13 ± 0.08) > **NM-6** (0.33 ± 0.13) > **Compound 5** (0.83 ± 0.58) > **NM-1** (1.16 ± 0.93) > **NM-4** (2.58 ± 0.46) > **NM-2** (3.55 ± 0.96) > **NM-3** (4.75 ± 0.88). The DPPH assay revealed that antioxidant activity within the triazole-carbohydrazide series is strongly influenced by the presence of H-donor sites and the ability of the molecule to delocalize unpaired electron density. The most active scavengers were the cyanoacryloyl-containing derivatives **(NM-7**,** NM-8**,** NM-10**; IC_50_ = 0.04 ± 0.05, 0.09 ± 0.05, and 0.06 ± 0.07 mg/mL, respectively). Their high activity is attributed to extended α, β-unsaturated carbonyl/nitrile conjugation, which stabilizes radical intermediates via resonance and facilitates single electron transfer (SET) and hydrogen atom transfer (HAT) pathways^40^, while the adjacent hydrazide N–H provides a labile hydrogen. In contrast, nitro benzylidene derivatives (**NM-3**,** NM-4**) displayed poor activity (IC_50_ ≥ 2.6 mg/mL), indicating that a single strong electron-withdrawing group is insufficient unless incorporated into a conjugated acceptor system. Bulky electron-donating substituents (diphenyl amino in **NM-1**) exhibited only moderate activity, likely due to steric hindrance and electronic localization that limit effective radical stabilization. Overall, these findings suggest that optimal antioxidant activity within this scaffold requires a synergistic balance of hydrogen- and electron-donating functionalities, particularly from hydrazide and triazole motifs, together with a conjugated cyanoacryloyl acceptor to stabilize the resulting radical species.

### Antibacterial activity

Hydrazide-containing compounds have demonstrated promising antibacterial activity, particularly against drug-resistant strains of *Staphylococcus aureus*. In this study, a series of synthetic hydrazide derivatives were evaluated in vitro against four clinically isolated bacterial strains: *Staphylococcus aureus*, *Bacillus subtilis*, *Klebsiella pneumoniae*, and *Enterobacter cloacae*^41^ (Table 2, **Figure S2**). **Compound 5** exhibited strong antibacterial activity against Gram-positive bacteria, with inhibition zones of 17 ± 1.04 **mm** for *B. subtilis* and 17 ± 1.09 **mm** for *S. aureus*. In contrast, it was inactive against the tested Gram-negative strains. Similarly, **compounds NM-2**,** NM-5**,** NM-6**,** NM-9**,** NM-11**, and **C** showed no detectable antibacterial activity against any of the tested strains as show in **Figure S2**. **Compound NM-7** showed selective but moderate activity against *S. aureus* (13 ± 1.06 mm), with no activity against the other strains tested. In comparison, the reference antibiotic **azithromycin (Ab)** exhibited superior and broader activity, with inhibition zones of 23 ± 1.03 **mm** for *B. subtilis*, 26 ± 1.04 **mm** for *S. aureus*, and 21 ± 1.01 **mm** for *K. pneumoniae*. Notably, **compound NM-8** demonstrated the broadest spectrum of activity, displaying inhibition zones of 13 ± 1.15 **mm** for *B. subtilis*, 17 ± 1.05 **mm** for *S. aureus*, and 12 ± 1.02 **mm** for both *K. pneumoniae* and *E. cloacae*. The broad-spectrum efficacy of **NM-8** may be attributed to the presence of a **chlorine (Cl)** substituent, which can enhance membrane permeability and interaction with bacterial targets. **Compound NM-10** also exhibited noteworthy activity against Gram-positive bacteria, with inhibition zones of 15 ± 1.13 **mm** for *B. subtilis* and 16 ± 1.08 **mm** for *S. aureus*. The enhanced activity of **NM-10** is likely due to the presence of electron-withdrawing nitro (-NO_2_) groups in its structure. These functional groups create localized electron-deficient regions that may facilitate stronger interactions with essential biomolecules such as proteins, nucleic acids, and enzymes^42^. Additionally, the significant dipole moment of **NM-10** could contribute to its high affinity for bacterial targets, thereby increasing its antibacterial efficacy^43^. However, azithromycin showed no activity against *E. cloacae*. Overall, the hydrazide derivatives showed greater effectiveness against Gram-positive bacteria than Gram-negative bacteria, which is consistent with the higher intrinsic resistance of Gram-negative organisms due to their outer membrane barrier. The structure–activity relationship (SAR) analysis indicates that the presence of electron-withdrawing groups (–NO_2_, Cl) plays a key role in enhancing antibacterial activity^44^. These findings suggest that hydrazide derivatives, particularly compounds (**5**,** NM-8**, and **NM-10)**, hold potential as lead structures for the development of new antibacterial agents targeting resistant Gram-positive pathogens.

**Table 2:** Antibacterial activity of the synthesized compounds **5** and **NM7-11** against tested microbes (NA = no activity, Ab = azithromycin as standard, C = DMSO as negative control).

Compound	Bacillus subtilis	S. aureus	Klebsiella Pneumonia	Enterobacter cloacae
5	17 ± 1.04	17 ± 1.09	NA	NA
NM-7	NA	13 ± 1.06	NA	NA
NM-8	13 ± 1.15	17 ± 1.05	12 ± 1.02	12 ± 1.02
NM-9	NA	NA	NA	NA
NM-10	15 ± 1.13	16 ± 1.08	NA	NA
NM-11	NA	NA	NA	NA
C (DMSO)	NA	NA	NA	NA
(Ab) azithromycin	23 ± 1.03	26 ± 1.04	21 ± 1.01	NA

### Synergistic antibacterial effects of Hydrazide derivatives combined with Azithromycin

The antibacterial efficacy of selected hydrazide derivatives was further assessed in combination with azithromycin to explore potential synergistic effects. Notably, even when azithromycin was applied at half its standard concentration, the combined formulations exhibited antibacterial activity comparable to that of the full-strength antibiotic, particularly against Gram-positive bacteria. This observation suggests that the tested compounds may enhance or preserve the antibiotic’s efficacy, potentially by modifying its molecular interactions or facilitating its uptake, without compromising its biological activity^45^. Compounds **NM-1**, **NM-3**, and **NM-4**, which demonstrated no detectable antibacterial activity when tested individually against Gram-negative bacteria, showed significant activity against Gram-positive strains when used in combination with azithromycin. Specifically, in the presence of the antibiotic, **NM-1**, **NM-3**, and **NM-4** produced inhibition zones of 19 ± 1.11 mm, 19 ± 1.09 mm, and 19 ± 1.02 mm, respectively, against *Bacillus subtilis*, and 21 ± 1.05 mm, 21 ± 1.06 mm, and 21 ± 1.12 mm, respectively, against *Staphylococcus aureus*. Although these values were slightly lower than those observed for azithromycin alone (23 ± 1.04 mm for *B. subtilis* and 23 ± 1.09 mm for *S. aureus*), the results clearly indicate additive effect Table 3. No antibacterial activity was observed against *Klebsiella pneumoniae* or *Enterobacter cloacae* for these compounds, either alone or in combination with azithromycin, indicating that the synergistic effect was selective to Gram-positive bacteria. This selectivity may be attributed to fundamental differences in cell wall composition, outer membrane permeability, and efflux pump activity between Gram-positive and Gram-negative organisms. Overall, these findings highlight the potential of hydrazide derivatives such as **NM-1**, **NM-3**, and **NM-4** as antibiotic adjuvants that can improve therapeutic outcomes against Gram-positive pathogens. Further mechanistic studies are warranted to elucidate the nature of this synergism and optimize such combinations for potential clinical application.

**Table 3:** Investigating the atrial antibiotic resistance to combat the drug’s resistance.

Compounds	Bacillus subtilis	Staphylococcus aureus	Klebsiella Pneumonia	Enterobacter cloacae
NM-1	19 ± 1.11	21 ± 1.05	NA	NA
NM-3	19 ± 1.09	21 ± 1.06	NA	NA
NM-4	19 ± 1.02	21 ± 1.12	NA	NA
C (DMSO)	NA	NA	NA	NA
(Ab) azithromycin	23 ± 1.04	23 ± 1.09	15 ± 1.05	NA

The antibacterial screening revealed clear structure–activity trends depending on both substituent type and microbial strain. **Comp. 5**,** NM-7**,** NM-8**, and **NM-10** produced measurable inhibition zones, particularly against *Bacillus subtilis* and *Staphylococcus aureus*, whereas Gram-negative strains (*K. pneumoniae*, *E. cloacae*) were less responsive, consistent with their intrinsic permeability barriers. Electron-withdrawing substituents enhanced antibacterial activity; for example, nitro- and chloro-substituted derivatives (**NM-3**,** NM-4**,** NM-10**) exhibited stronger effects against Gram-positive strains, likely due to increased electrophilicity that favors interactions with bacterial enzymes and nucleophilic biomolecules. The cyanoacyl and cyanoacryloyl groups (**Comp. 5**,** NM-7**,** NM-8**) may further contribute by acting as Michael acceptors, enabling potential covalent modification of protein nucleophiles and disruption of bacterial function. Notably, **NM-8**, which combines a chloro substituent with a cyanoacryloyl group, displayed the broadest spectrum, suggesting that synergistic electron-withdrawing effects and extended conjugation enhance both target engagement and possibly membrane penetration. By contrast, the diphenyl amino derivative (**NM-1**) showed negligible activity, consistent with the unfavorable steric and electronic effects of bulky electron-donating substituents. This finding highlights the distinct structural requirements underlying radical scavenging and antibacterial mechanisms. Compounds **NM-1**,** NM-3**, and **NM-4**, despite showing limited direct activity against Gram-negative bacteria, exhibited marked potentiation when combined with azithromycin. At half the standard antibiotic dose, these combinations produced inhibition zones comparable to full-strength azithromycin, particularly against *S. aureus* and *B. subtilis*. The observed synergism may arise from several mechanisms, including increased bacterial membrane permeability facilitating antibiotic uptake, modulation of efflux pump activity, or complementary interactions with bacterial targets^46^. Such effects suggest that triazole carbohydrazide derivatives may function as antibiotic adjuvants, enhancing the efficacy of conventional antibiotics against resistant strains. Overall, electron-withdrawing and electrophilic substituents appear to strengthen antibacterial effects, while certain derivatives also display synergy with azithromycin, underscoring their dual potential as both direct antimicrobials and resistance-modifying agents. This duality highlights the importance of rational substituent selection in tailoring compounds for multifunctional therapeutic applications.

## Docking analysis

Docking studies showed that the compounds tested fit well into the active site of the E. coli DNA gyrase B protein (**PDB ID: 6YD9**), with binding free energy scores ranging from − 6.3183 to −7.4696 kcal/mol. Based on the interaction profiles and docking scores presented in Table 4, these ligands engage significantly with key residues through non-covalent interactions and exhibit an optimal fit within the protein’s binding pocket^47,48^.

**Table 4:** Specific interactions between the ligands and the target proteins, as well as predictive Docking scores.

Cpd. No.	Binding energy (S) Kcal/mol	RMSD	Distance (Å)	Binding interactions		
				Ligand	Receptor	Interaction type
NM-1	−6.3183	1.8221	3.01	N15 -nitrile group	Arg 136	H-acceptor
			3.55	Triazole ring	Pro 79	pi-H
			4.04	Benzene ring	Ile 94	pi-H
NM-3	−6.5985	1.4663	4.10	Benzene ring	Arg 136	pi-cation
NM-4	−7.1205	0.8302	3.05	C3 from benzene ring	Asp 73	H-donor
5	−7.4696	1.2063	3.13	N17- nitrile group	Asp 73	H-donor
			3.98	Triazole ring	Ile 78	pi-H
NM-7	−7.2626	1.4014	3.43	Benzene ring	Arg 136	π -cation
NM-8	−6.9653	0.8455	3.18	N30 -nitrile group	Arg 136	H-acceptor
			3.37	N30 -nitrile group	Arg 136	H-acceptor
NM-10	−6.9983	1.8767	3.18	N32 -nitrile group	Gly 77	H-acceptor

**Fig. 10:**
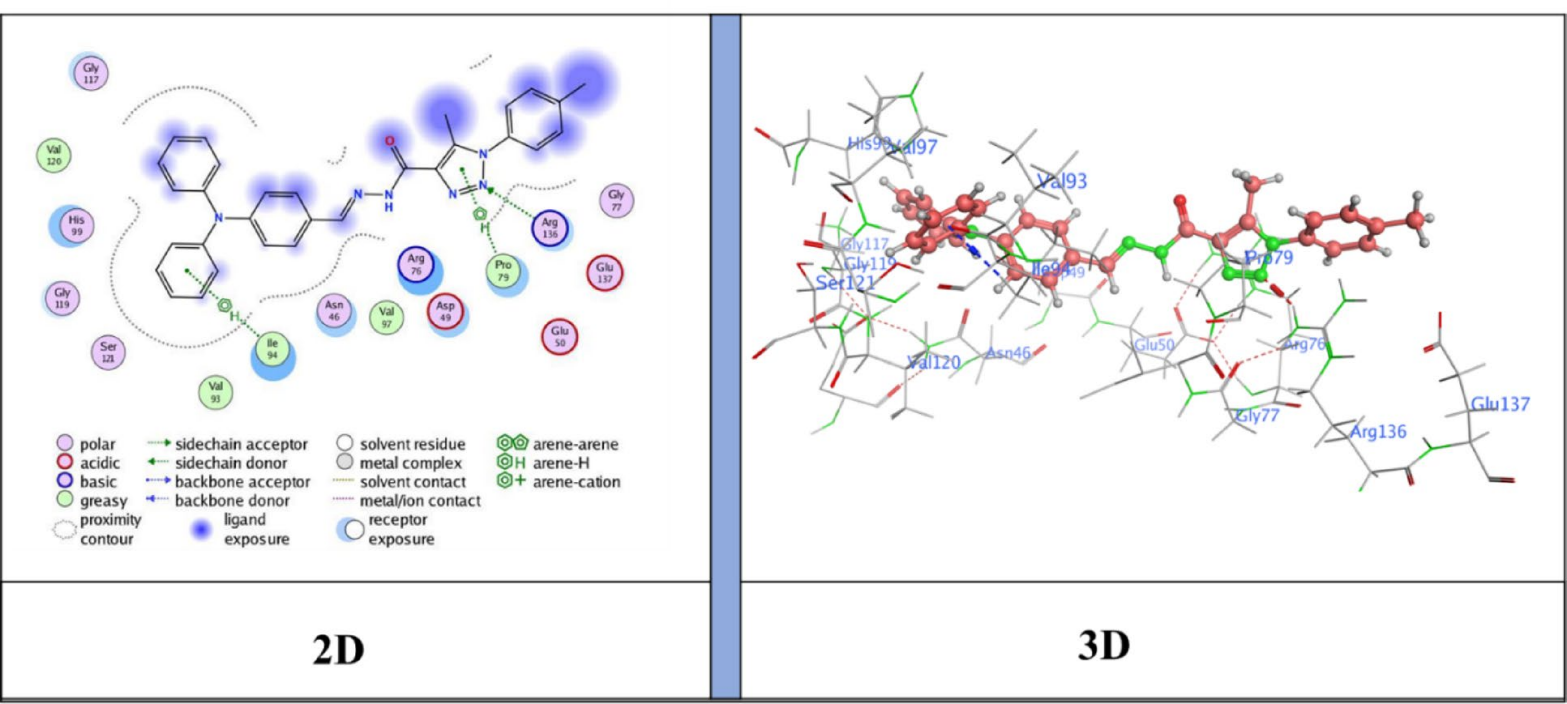
Ligand (**NM-1**) interaction with protein (**6YD9**).

**Fig. 11:**
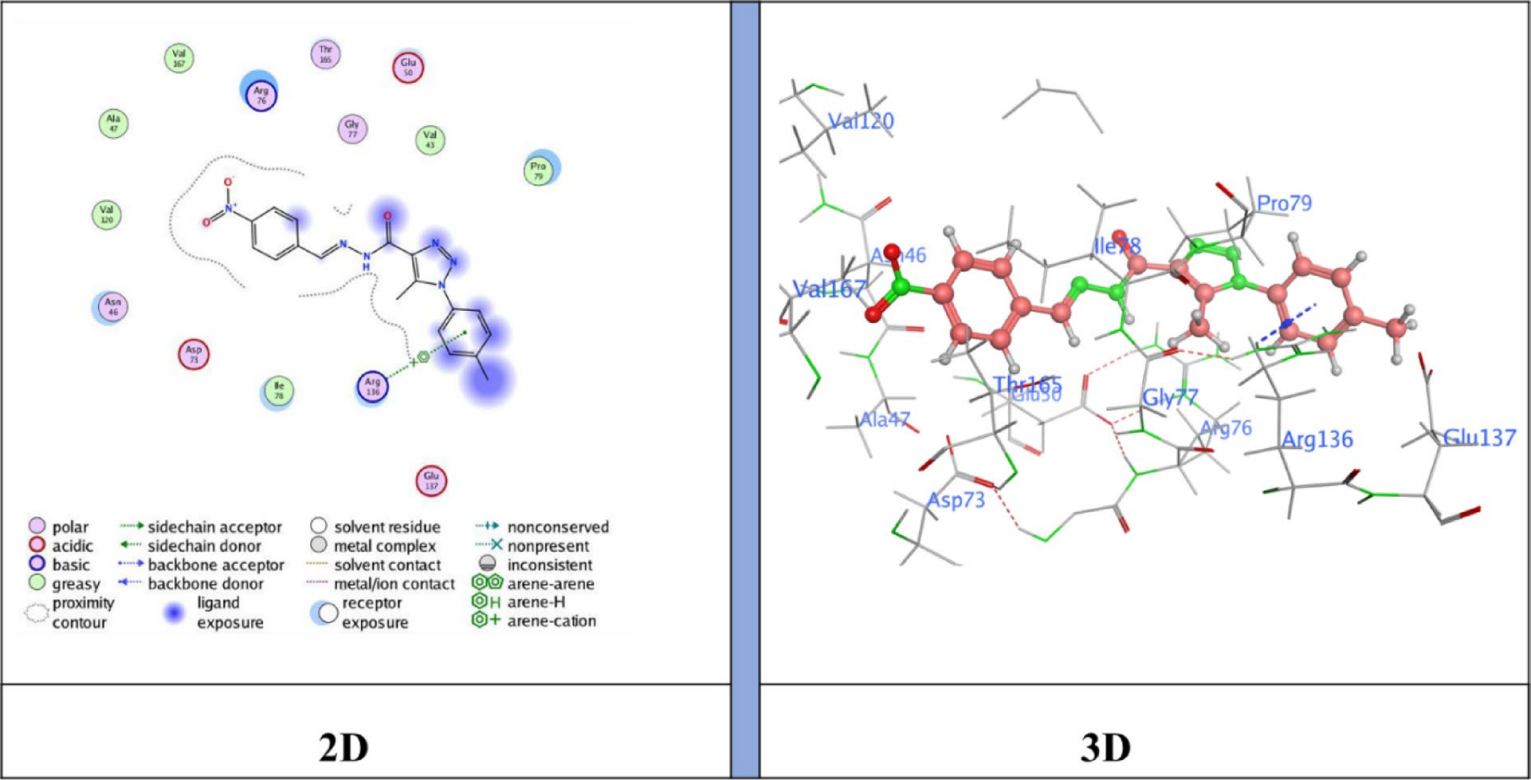
Ligand (**NM-3**) interaction with protein (**6YD9**).

**Fig. 12:**
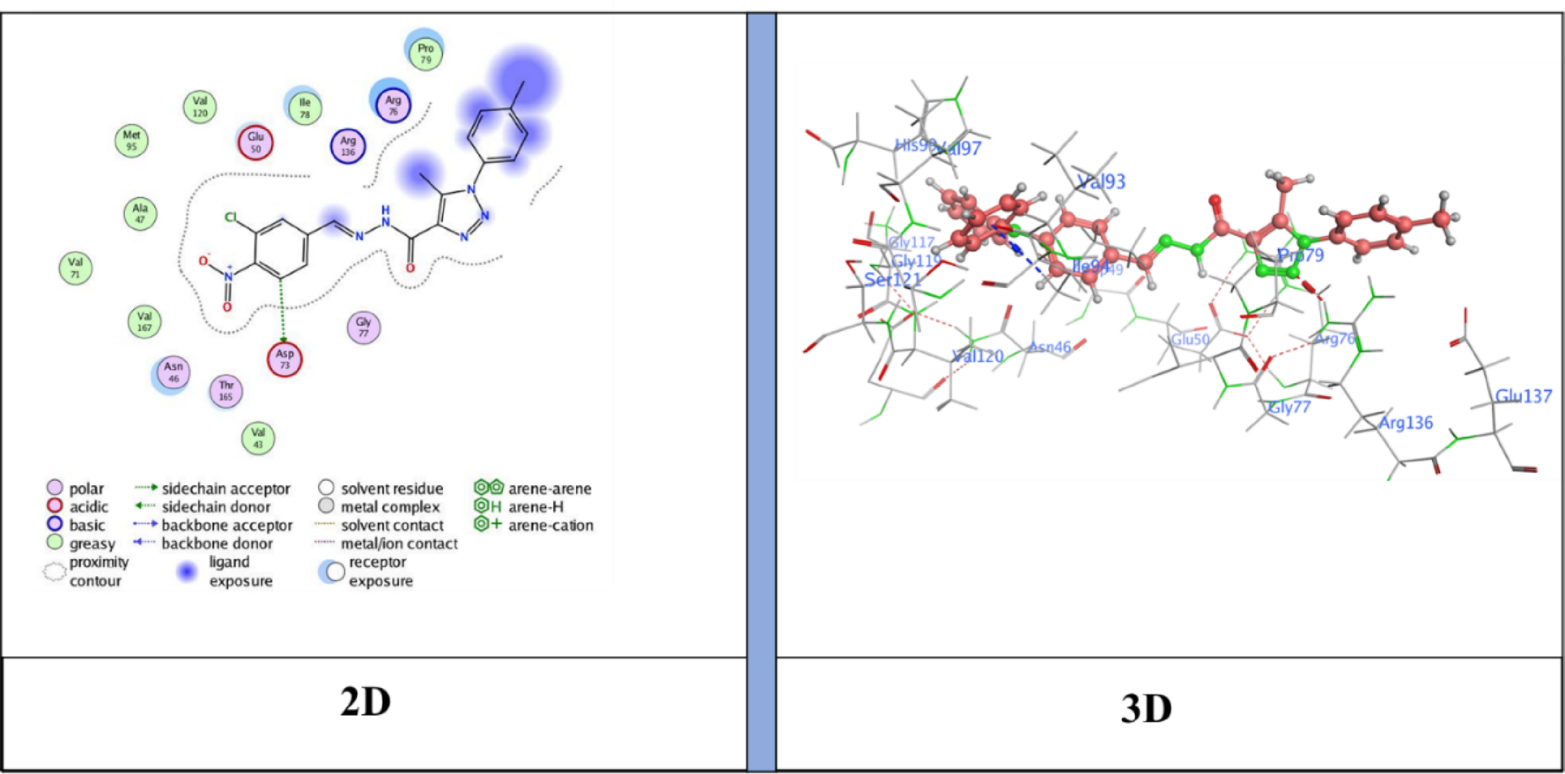
Ligand (**NM-4**) interaction with protein (**6YD9**).

**Fig. 13:**
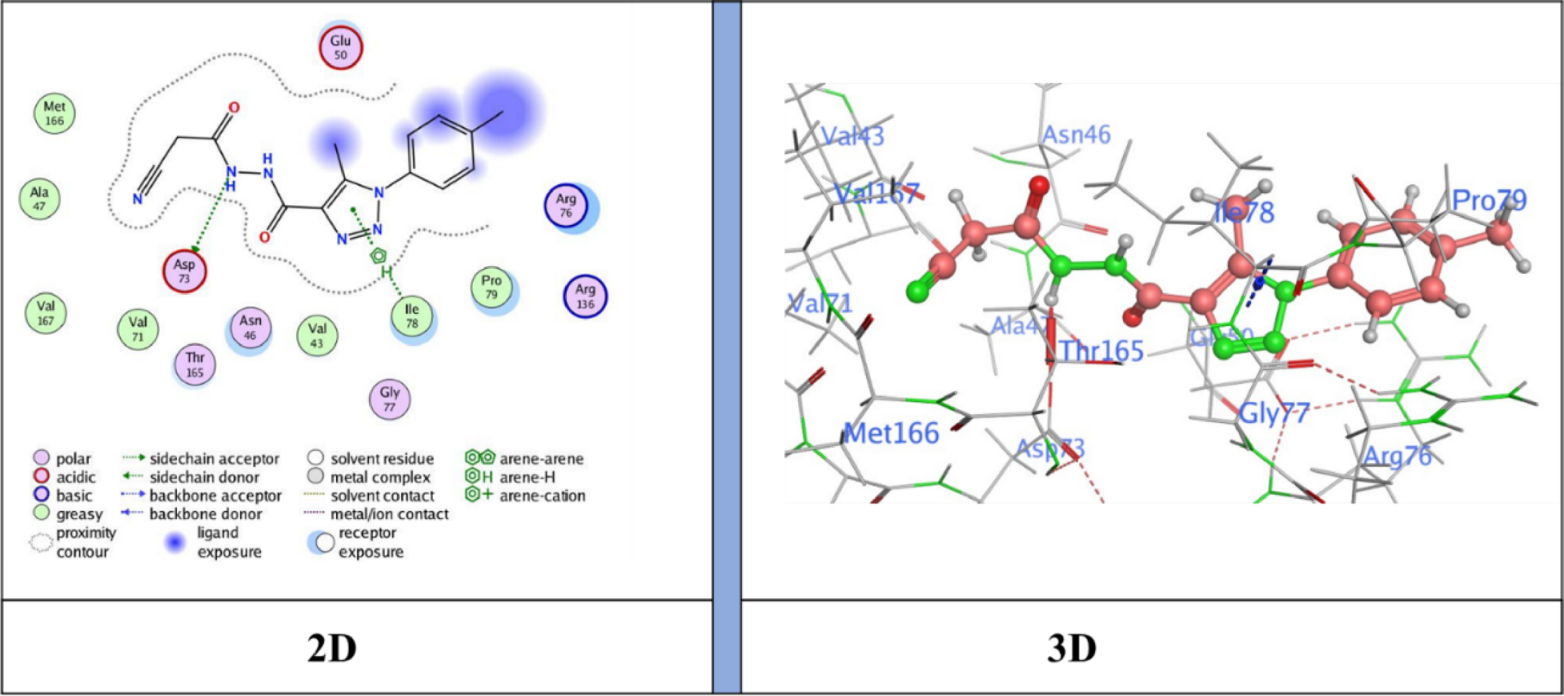
Ligand (**5**) interaction with protein (**6YD9**).

**Fig. 14:**
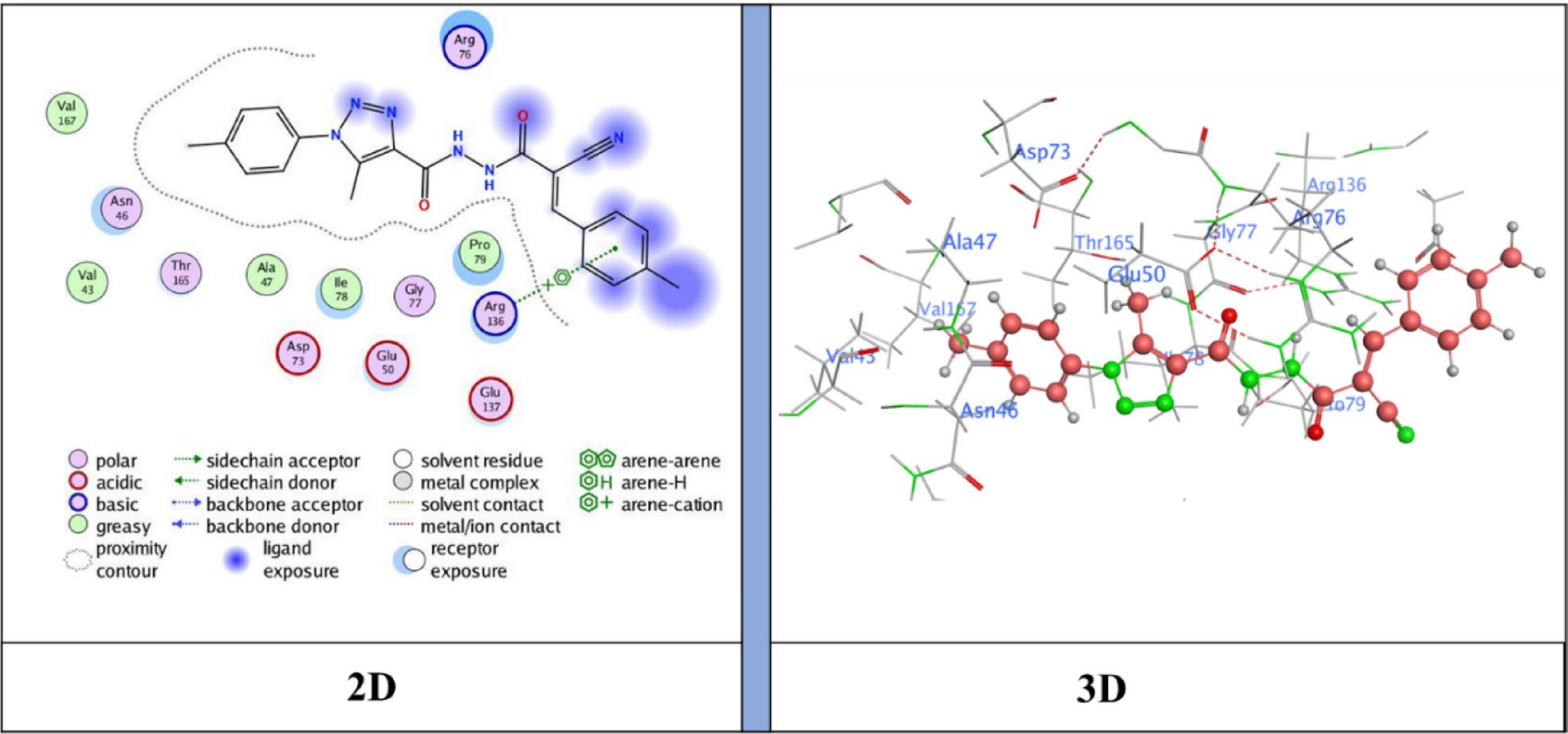
Ligand (**NM-7**) interaction with protein (**6YD9**).

**Fig. 15:**
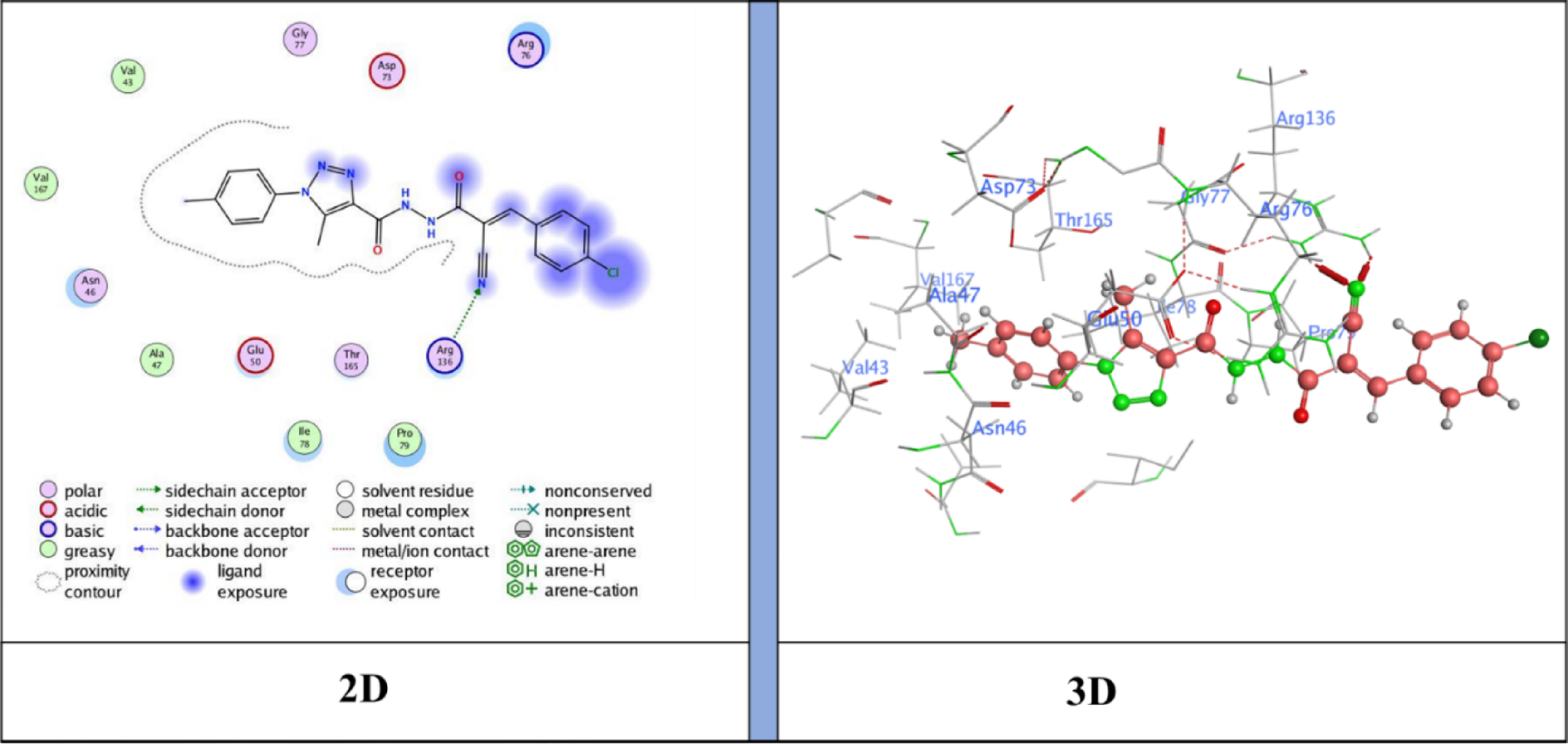
Ligand (**NM-8**) interaction with protein (**6YD9**).

**Fig. 16:**
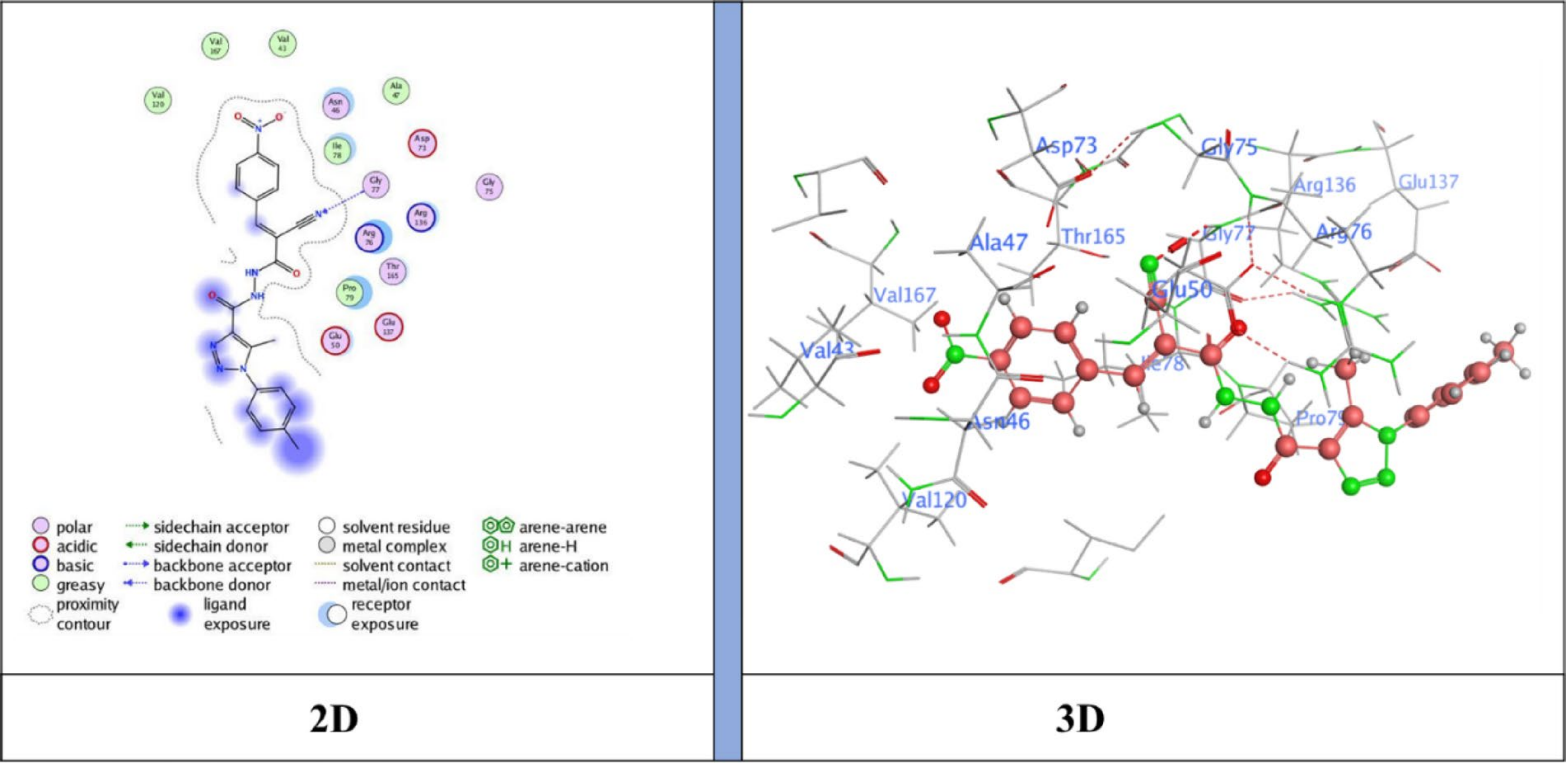
Ligand (**NM-10**) interaction with protein (**6YD9**).

The docking results showed binding energies ranging from − 6.31 to −7.47 kcal/mol, with **compound 5** (*N*’-(2-cyanoacetyl)−5-methyl-1-(*p*-tolyl)−1*H*−1,2,3-triazole-4-carbohydrazide) exhibiting the highest binding affinity (−7.47 kcal/mol), followed by **NM-7** (−7.26 kcal/mol), **NM-4** (−7.12 kcal/mol), **NM-10** (−7.00 kcal/mol), **NM-8** (−6.96 kcal/mol), **NM-3** (−6.59 kcal/mol), and **NM-1** (−6.31 kcal/mol). The interaction profiles revealed important structure–activity insights: **NM-1** (diphenyl amino derivative) displayed the weakest affinity, forming π–H contacts with Pro79 and Ile94 and H-bond acceptor interaction with Arg136 Fig. 10. The reduced score is likely due to steric hindrance from the bulky diphenyl amino group, which limited deeper binding. **NM-3** (4-nitrobenzylidene derivative) showed stronger binding than **NM-1**, stabilized by π–cation interactions with Arg136 Fig. 11. The nitro substituent enhanced polarity and improved polar surface contacts. **NM-4** (3-chloro-4-nitrobenzylidene derivative) achieved further enhancement through both chloro and nitro substituents, generating an H-donor bond with Asp73 that optimized orientation in the binding pocket Fig. 12. **Compound 5** (cyanoacetyl derivative), the best binder, combined hydrogen bonding (Asp73) and π–H interactions (Ile78), indicating that the cyanoacetyl moiety plays a central role in stabilizing ligand binding Fig. 13. **NM-7** (cyanoacryloyl derivative) benefited from π–cation interactions with Arg136, and its extended conjugation allowed better fit within the binding pocket Fig. 14. **NM-8** (chloro-substituted cyanoacryloyl derivative) established two H-acceptor interactions with Arg136 via its nitrile group Fig. 15, supporting moderate yet stable binding. **NM-10** (nitro-substituted cyanoacryloyl derivative) formed an H-bond with Gly77 through the nitrile nitrogen and engaged in additional electrostatic stabilization due to its nitro group Fig. 16. These findings confirm that electron-withdrawing groups (-NO_2_, - Cl) and cyanoacetyl/cyanoacryloyl functionalities strongly enhance binding by contributing additional hydrogen bonding, π–cation, and polar interactions. Conversely, bulky substituents (as in **NM-1**) negatively influence binding through steric hindrance.

### Antibacterial activity and correlation with molecular Docking

These computational findings correlated well with the in vitro antibacterial assays. Compounds **NM-1**, **NM-3**, and **NM-4** displayed notable inhibition of *B. subtilis* (19 mm) and *S. aureus* (21 mm), consistent with their docking scores (–6.31 to − 7.12 kcal/mol). Their nitro and chloro substituents are likely to enhance electron-withdrawing effects, improving both binding affinity and bacterial cell interactions. Compound **5**, which displayed the lowest docking energy (–7.47 kcal/mol), inhibited *Bacillus subtilis* (17 ± 1.04 mm) and *Staphylococcus aureus* (17 ± 1.09 mm). Its predicted interactions with Asp73 (hydrogen bonding) and Ile78 (π–H interaction) likely contribute to its broad Gram-positive activity. Similarly, **NM-7**, stabilized by π–cation interactions with Arg136, showed selective inhibition of *S. aureus* (13 ± 1.06 mm), consistent with strong binding affinity but potentially limited penetration across other bacterial strains. Interestingly, **NM-8** (–6.96 kcal/mol) demonstrated the widest antibacterial spectrum, being active against *B. subtilis*, *S. aureus*, *Klebsiella pneumoniae*, and *Enterobacter cloacae*. The presence of a chloro-substituted cyanoacryloyl group likely enhances polarity, facilitating interactions with diverse bacterial targets. In contrast, **NM-9**, **NM-11** lacked significant antibacterial activity, aligning with the absence of favorable docking interactions. **NM-10**, which formed nitrile-mediated hydrogen bonds with Gly77, also exhibited consistent inhibition of *B. subtilis* (15 ± 1.13 mm) and *S. aureus* (16 ± 1.08 mm). These results demonstrate that favorable docking energies, particularly those involving cyanoacetyl, nitro, and chloro substituents, are associated with enhanced antibacterial activity. The correlation was most evident against Gram-positive strains (*B. subtilis* and *S. aureus*), underscoring the importance of structural features in determining both binding affinity and biological activity^49^.

## Experimental

Melting points (m.p.) were determined using a Gallenkamp melting point apparatus. Infrared (IR) spectra were recorded on a BRUKER INVENIO FT-IR spectrometer. ^1^H and ^13^C NMR spectra were obtained on JEOL ECA-500 (500 MHz for ^1^H and 125 MHz for ^13^C) using DMSO-d6 as solvent. Chemical shifts (δ) are reported in parts per million (ppm). The purity of the synthesized compounds was verified by thin-layer chromatography (TLC) on silica gel plates, employing petroleum ether/ethyl acetate mixtures as eluents, with visualization under a UV lamp. Mass spectra and elemental analyses were carried out on a Thermo DSQ II spectrometer at the Faculty of Science, Al-Azhar University.

### Synthesis of compounds NM-1–6

A mixture of 5-methyl-1-(*p*-tolyl)−1*H*−1,2,3-triazole-4-carbohydrazide **(1)** (0.231 g, 1 mmol), various substituted aldehydes (**2a-d**) (1 mmol), 30 mL of dry ethanol, and a few drops of acetic acid was placed in a 50 mL round-bottom flask. The reaction mixture was refluxed for 3 h and then allowed to cool to room temperature. The resulting precipitation was filtered and washed with hot ethanol to afford compounds **NM-1** to **NM-4**. For the synthesis of **NM-5**, hydrazide **(1)** (0.231 g, 1 mmol) was reacted with indoline-2,3-dione (**3**) (0.15 g, 1 mmol) in 20 mL of ethanol under reflux for 2 h. The resulting solid was collected and recrystallized from a heated ethanol/1,4-dioxane solution (2:1) to yield yellow crystals. To prepare **NM-6**, compound **NM-5** (0.36 g, 1 mmol) was dissolved in 10 mL of acetic anhydride and refluxed for 5 h. After cooling, the solid product was isolated and recrystallized from ethanol, yielding **NM-6** as yellow crystals.

#### N’-(4-(Diphenylamino) benzylidene)−5-methyl-1-(p-tolyl)−1 H-1,2,3-triazole-4-carbohydrazide (NM-1)

Pale yellow powder (68% yield); m.p. 286–288 °C. IR (ῡ, cm^− 1^): 3314 (N-H), 3060 (= CH), 1686 (C = O),^1^H NMR (DMSO-*d*_6_): *δ* (ppm): 2.41 (s, 3 H, -CH_3_), 2.49 (s, 3 H, -CH_3_), 6.95 (d, *J* = 9.00 Hz, 2 H, Ar-H), 7.07 (d, *J* = 7.00 Hz, 4 H, Ar-H), 7.11 (d, *J* = 7.50 Hz, 2 H, Ar-H), 7.34 (t, *J* = 8.00 Hz, 4 H, Ar-H), 7.43 (d, *J* = 9.00 Hz, 2 H, Ar-H), 7.52 (d, *J* = 9.00 Hz, 2 H, Ar-H), 7.56 (d, *J* = 8.00 Hz, 2 H, Ar-H), 8.47 (s, 1H, CH = N), 12.01 (s, 1H, N-H). ^13^C NMR (DMSO-*d*_6_): *δ* (ppm): 9.4, 20.7, 121.4 (2 C), 124.0 (2 C), 125.0 (4 C), 125.2 (2 C), 127.6, 128.3 (2 C), 129.7 (4 C), 130.1 (2 C), 132.8, 137.3, 137.7, 139.9, 146.5 (2 C), 147.7, 148.9, 157.0. Mass analysis (m/z, %): 486 (M^+,^ 28.04), 438 (44.10), 434 (77.31), 423 (52.27), 415 (34.66), 409 (40.82), 386 (100.00), 362 (37.05), 366 (28.88), 221 (27.39), 176 (43.05), 164 (45.06), 151 (50.73), 150 (41.66), 80 (44.41). Analysis for: C_30_H_26_N_6_O (486.58): Calculated: C, 74.05; H, 5.39; N, 17.27. Found: C, 74.11; H, 5.40; N, 17.33%.

#### 4-((2-(5-Methyl-1-(p-tolyl)−1 H-1,2,3-triazole-4-carbonyl) hydrazineylidene)methyl)benzoic acid (NM-2)

White crystals (68% yield); m.p. above 300 °C. IR (ῡ, cm^− 1^): 3330 (N-H), 2976 (C-H) aliphatic, 1676 (C = O). ^1^H NMR (DMSO-*d*_6_): *δ* (ppm): 2.41 (s, 3 H, -CH_3_), 2.49 (s, 3 H, -CH_3_), 7.44 (d, *J* = 8.00 Hz, 2 H, Ar-H), 7.53 (d, *J* = 8.00 Hz, 2 H, Ar-H), 7.81 (d, *J* = 8.00 Hz, 2 H, Ar-H), 8.00 (d, *J* = 8.00 Hz, 2 H, Ar-H), 8.63 (s, 1H, CH = N), 12.33 (s, 1H, N-H), 13.09 (s, 1H, -COOH). ^13^C NMR (ppm): *δ* 9.4, 20.8, 125.2 (2 C), 127.1 (2 C), 129.8 (2 C), 130.1 (2 C), 131.6, 132.7, 137.1, 138.1, 138.5, 139.9, 146.8, 157.4, 166.9. Mass analysis (m/z, %): 363 (M^+,^ 24.45), 322 (82.09), 229 (30.28), 211 (49.50), 206 (100.00), 192 (63.41), 189 (30.39), 164 (86.26), 157 (47.39), 145 (39.65), 123 (52.76), 120 (31.48), 98 (30.36), 95.05 (62.70). Analysis for: C_19_H_17_N_5_O_3_ (363.38): Calculated: C, 62.80; H, 4.72; N, 19.27. Found: C, 62.89; H, 4.71; N, 19.30%.

#### 5-Methyl-N’-(4-nitrobenzylidene)−1-(p-tolyl)−1 H-1,2,3-triazole-4-carbohydrazide (NM-3)

Off white crystals (80% yield); m.p. 265–267 °C. IR (ῡ, cm^− 1^): 3316 (N-H), 3040 (= C-H), 1681 (C = O). ^1^H NMR (DMSO-*d*_6_): *δ* (ppm): 2.41 (s, 3 H, -CH_3_), 2.49 (s, 3 H, -CH_3_), 7.44 (d, *J* = 8.00 Hz, 2 H, Ar-H), 7.53 (d, *J* = 8.00 Hz, 2 H, Ar-H), 7.95 (d, *J* = 9.00 Hz, 2 H, Ar-H), 8.30 (d, *J* = 9.00 Hz, 2 H, Ar-H), 8.68 (s, 1H, CH = N), 12.48 (s, 1H, -NH). ^13^C NMR (DMSO-*d*_6_): *δ* (ppm): 9.06, 20.4, 123.8, 123.8, 124.8, 125.1, 127.7 (2 C), 129.7, 129.9, 130.0, 132.6, 136.9, 137.9, 139.7, 140.5, 145.4, 147.7. Mass analysis (m/z, %): 364 (M^+,^ 14.85), 326 (28.42), 273 (32.25), 147 (34.80), 127 (42.87), 108 (29.89), 83 (37.91), 79 (25.18), 71 (51.05), 69 (69.22), 67 (41.48), 57 (47.33), 55 (100.00), 54 (41.14), 45 (30.10). Analysis for: C_18_H_16_N_6_O_3_ (364.37): Calculated: C, 59.34; H, 4.43; N, 23.07. Found: C, 59.40; H, 4.42; N, 23.11%.

#### N’-(3-Chloro-4-nitrobenzylidene)−5-methyl-1-(p-tolyl)−1 H-1,2,3-triazole-4-carbohydrazide (NM-4)

Brown powder (65% yield); m.p. above 300 °C. IR (ῡ, cm^− 1^): 3298 (N-H), 1642 (C = O). ^1^H NMR (DMSO-*d*_6_): *δ* (ppm): 2.41 (s, 3 H, -CH_3_), 2.49 (s, 3 H, -CH_3_), 6.83 (d, *J* = 8.00 Hz, 2 H, Ar-H), 7.43 (d, *J* = 8.00 Hz, 2 H, Ar-H), 7.51 (d, *J* = 2.50 Hz, 1H, Ar-H), 7.53 (d, *J* = 3.50 Hz, 1H, Ar-H), 8.45 (s, 1H, Ar-H), 9.93 (s, 1H, CH = N), 11.93 (s, 1H, N-H). ^13^C NMR (DMSO-*d*_6_): *δ* (ppm): 9.4, 20.8, 115.7 (2 C),125.2 (2 C), 125.4, 128.9 (2 C), 130.1 (2 C), 132.8, 137.3, 137.6, 139.9, 148.3, 157.0, 159.4. Mass analysis (m/z, %): 398 (M^+,^ 11.51), 388 (72.18), 386 (42.06), 384 (33.54), 381 (38.85), 374 (57.52), 371 (32.02), 367 (38.40), 353 (37.79), 335 (46.83), 319 (100.00), 302 (40.91), 133 (44.85), 126 (50.04), 122 (33.37), 116 (36.76). Analysis for: C_18_H_15_ClN_6_O_3_ (398.81): Calculated: C, 54.21; H, 3.79; Cl, 8.89; N, 21.07. Found: C, 54.29; H, 3.80; N, 21.12%.

#### 5-Methyl-N’-(2-oxoindolin-3-ylidene)−1-(p-tolyl)−1 H-1,2,3-triazole-4-carbohydrazide (NM-5)

Yellow crystal (85% yield); m.p. above 300 °C. IR (ῡ, cm^− 1^): 3232 (N-H), 1702 and 1671 (C = O). ^1^H NMR (DMSO-*d*_6_): *δ* (ppm): 2.40 (s, 3 H, -CH_3_), 2.49 (s, 3 H, -CH_3_), 6.92 (d, *J* = 8.00 Hz, 1H, Ar-H), 7.08 (t, *J* = 7.00 Hz, 1H, Ar-H), 7.36 (t, *J* = 7.00 Hz, 1H, Ar-H), 7.43 (d, *J* = 7.50 Hz, 2 H, Ar-H), 7.52 (d, *J* = 8.00 Hz, 2 H, Ar-H), 7.57 (d, *J* = 7.50 Hz, 1H, Ar-H), 11.32 (s, 1H, N-H(. ^13^C NMR (DMSO-*d*_6_): *δ* (ppm): 9.1, 20.4, 110.8, 119.7, 120.6, 122.3, 124.9 (2 C), 129.8 (2 C), 131.4, 132.4, 136.3, 137.6, 138.6, 139.8, 142.3, 157.2, 162.4. Analysis for: C_19_H_16_N_6_O_2_ (360.38): Calculated: C, 63.33; H, 4.48; N, 23.32. Found: C, 63.42; H, 4.49; N, 23.39%.

#### 3’-Acetyl-5’-(5-methyl-1-(p-tolyl)−1 H-1,2,3-triazol-4-yl)−3’H-spiro[indoline-3,2’-^1,3,4^ oxadiazol]−2-one (NM-6)

Yellow crystal (80% yield); m.p. above 300 °C. IR (ῡ, cm^− 1^): 3231 (N-H), 1703 and 1670 (C = O). ^1^H NMR (DMSO-*d*_6_): *δ* (ppm): 1.92 (s, 3 H, -CH_3_), 2.45 (s, 3 H, -CH_3_), 2.60 (s, 3 H, -CH_3_), 6.97 (d, *J* = 8.00 Hz, 1H, Ar-H), 7.14 (t, *J* = 7.50 Hz, 1H, Ar-H), 7.40 (d, *J* = 7.50 Hz, 1H, Ar-H), 7.48 (d, *J* = 8.50 Hz, 2 H, Ar-H), 7.57 (d, *J* = 8.50 Hz, 2 H, Ar-H), 7.62 (d, *J* = 7.50 Hz, 1H, Ar-H), 11.34 (s, 1H, N-H). ^13^C NMR (DMSO-*d*_6_): *δ* (ppm): 9.9, 21.2, 21.5, 111.6, 120.4, 121.4, 123.1, 125.7 (3 C), 130.6 (2 C), 132.2, 133.0, 136.9, 138.4, 139.5, 140.6, 143.0, 163.1, 172.5. Mass analysis (m/z, %): 402 (M^+,^ 12.42), 368 (67.53), 367 (42.93), 338 (29.76), 313 (48.71), 311 (37.38), 306 (30.47), 193 (43.49), 191 (33.70), 101 (26.67), 97 (37.89), 92 (100.00), 90 (25.66), 82 (50.87). Analysis for: C_21_H_18_N_6_O_3_ (402.41): Calculated: C, 62.68; H, 4.51; N, 20.88, Found: C, 62.77; H, 4.50; N, 20.93%.

### Synthesis of N’-(2-cyanoacetyl)−5-methyl-1-(p-tolyl)−1 H-1,2,3-triazole-4-carbohydrazide (5)

In a dry 50 mL round-bottom flask, 0.23 g (1 mmol) of 5-methyl-1-(*p*-tolyl)−1*H*−1,2,3-triazole-4-carbohydrazide **(1)** and 0.16 g (1 mmol) of freshly prepared 3-(3,5-dimethyl-1*H*-pyrazol-1-yl)−3-oxopropanenitrile **(4)** were dissolved in 30 mL of dry dioxane. The reaction mixture had undergone reflux for a 3 h. The off-white precipitate was generated and isolated through filtration following the cooling of the solution to ambient temperature, yielding compound (**5**). Off-white powder with a yield of 70%; melting point = 178–180 °C^11^.

Off white (75% yield), m.p. 178–180 °C. IR (ῡ, cm^− 1^): 3228 (N-H), 3038 (= C-H), 2933 (C-H) aliphatic, 2258 (C ≡ N), 1702 and 1665 (C = O). ^1^H NMR (DMSO-*d*_6_): *δ* (ppm): 2.41 (s, 3 H, -CH_3_), 2.48 (s, 3 H, -CH_3_), 3.79 (s, 2 H, -CH_2_), 7.43 (d, *J* = 8.00 Hz, 2 H, Ar-H), 7.50 (d, *J* = 8.00 Hz, 2 H, Ar-H), 10.29 (s, 1H, -NH), 10.59 (s, 1H, -NH). ^13^C NMR (DMSO-*d*_6_): *δ* (ppm): 9.3, 20.8, 23.9, 115.7, 125.3 (2 C), 130.1 (2 C), 132.8, 136.4, 137.7, 139.9, 159.9, 161.8. Analysis for: C_14_H_14_N_6_O_2_ (298.12): Calculated: C, 56.37; H, 4.73; N, 28.17. Found: C, 56.47; H, 4.74; N, 28.20%.

### General synthesis of compounds (NM-7–11)

The *N*’-(2-cyanoacetyl)−5-methyl-1-(*p*-tolyl)−1*H*−1,2,3-triazole-4-carbohydrazide (**5**) (0.29 g, 1 mmol) slurry was placed in a dry 100 mL RB flask that 25 mL of ethanol was used to dissolve, and then various substituted aldehydes were added (1 mmol) **(***p*-methylbenzaldehyde (**6a**), *p*-chlorobenzaldehyde (**6b**), *p*-formylbenzoic acid (**6c**), *p*-nitrobenzaldehyde (**6d**), and 0.3 mL of piperidine was added. The reaction mixture was refluxed for 3 h to give product **NM-7–10**. To a solution of compound (**5**) (0.29 g, 1mmol) in absolute ethanol (20 mL), salicylaldehyde (0.12 g, 1mmol) was added. The reaction mixture was heated under reflux at 200 °C for 3 h, and then allowed to cool. The precipitate that formed was filtered off, washed with ethanol, dried and recrystallized from ethanol to afford compound (**NM-11**).

#### N’-(2-Cyano-3-(p-tolyl) acryloyl)−5-methyl-1-(p-tolyl)−1 H-1,2,3-triazole-4-carbohydrazide (NM-7)

Yellow powder (70%) yield; m.p. above 300 °C. IR (ῡ, cm^− 1^): 3300 (N-H), 2947 (C-H) aliphatic, 2215 (C ≡ N), 1676 (C = O). ^1^H NMR (DMSO-*d*_6_): *δ* (ppm): 2.35 (s, 3 H, -CH_3_), 2.41 (s, 3 H, -CH_3_), 2.49 (s, 3 H, -CH_3_), 7.30 (d, *J* = 7.50 Hz, 2 H, Ar-H), 7.42 (d, *J* = 8.00 Hz, 2 H, Ar-H), 7.51 (d, *J =* 8.50 Hz, 2 H, Ar-H), 7.80 (d, *J* = 8.50 Hz, 2 H, Ar-H), 7.92 (s, 1H, CH = C), 9.54 (s, 1H, N-H).^13^C NMR (DMSO-*d*_6_): *δ* (ppm): 9.7, 21.2, 21.5, 110.7, 119.3, 125.6 (3 C), 129.7 (2 C), 130.0, 130.5 (2 C), 131.3, 133.5, 136.3, 138.8, 140.1, 140.9, 140.2, 155.1, 160.2. Mass analysis (m/z, %): 400 (M^+,^ 15.18), 371(49.12), 370 (54.74), 326 (30.68), 247 (30.63), 246 (91.96), 244 (44.44), 233 (51.38), 158 (51.65), 144 (100.00), 73 (39.57), 71 (45.08), 64 (36.41), 62 (45.82), 56 (35.70), 52 (44.01). Analysis for: C_22_H_20_N_6_O_2_ (400.44): Calculated: C, 65.99; H, 5.03; N, 20.99. Found: C, 66.08; H, 5.02; N, 21.04%.

#### N’-(3-(4-Chlorophenyl)−2-cyanoacryloyl)−5-methyl-1-(p-tolyl)−1 H-1,2,3-triazole-4-carbohydrazide (NM-8)

Orange powder (82% yield); m.p. above 300 °C. IR (ῡ, cm^− 1^): 3344 (N-H), 3033 (= C-H), 2217 (C ≡ N), 1652 (C = O). ^1^H NMR (DMSO-*d*_6_): *δ* (ppm): 2.41 (s, 3 H, -CH_3_), 2.49 (s, 3 H, -CH_3_), 7.42 (d, *J* = 7.50 Hz, 2 H, Ar-H), 7.51 (d, *J* = 7.50 Hz, 2 H, Ar-H), 7.54 (d, *J* = 7.50 Hz, 2 H, Ar-H), 7.90 (s, 1H, Ar-H), 7.91 (s, 2 H, Ar-H), 10.77 (s, 1H, N-H). ^13^C NMR (DMSO-*d*_6_): *δ* (ppm): 9.7, 21.2, 112.9, 119.1, 125.6 (2 C), 129.5 (2 C), 130.6 (2 C), 131.3 (2 C), 133.0, 133.4, 135.1, 136.4, 138.7, 140.2, 142.3, 155.0, 159.8. Mass analysis (m/z, %): 420 (M^+,^ 100.00), 411 (60.65), 402 (68.83), 400 (36.81), 392 (37.22), 369 (37.60), 337 (34.30), 310 (77.16), 277 (33.68), 265 (33.88), 250 (57.62), 192 (39.01), 189 (35.84), 147 (33.40), 134 (75.99). Analysis for: C_21_H_17_ClN_6_O_2_ (420.86): Calculated: C, 59.93; H, 4.07; N, 19.97. Found: C, 59.99; H, 4.05; N, 20.01%.

#### 4-(2-Cyano-3-(2-(5-methyl-1-(p-tolyl)−1 H-1,2,3-triazole-4-carbonyl) hydrazineyl)−3-oxoprop-1-en-1-yl) benzoic acid (NM-9)

Yellow powder (75% yield); m.p. above 300 °C. IR (ῡ, cm^− 1^): 3332 (N-H),3039 (= C-H), 2217 (C ≡ N), 1659 (C = O). ^1^H NMR (DMSO-*d*_6_): *δ* (ppm): 2.41 (s, 3 H, -CH_3_), 2.49 (s, 3 H, -CH_3_), 7.42 (d, *J* = 8.00 Hz, 2 H, Ar-H), 7.51 (d, *J* = 9.00 Hz, 2 H, Ar-H), 7.79 (d, *J* = 8.00 Hz, 2 H, Ar-H), 7.90 (d, *J* = 2.50 Hz, 2 H, Ar-H), 7.92 (s, 1H, CH = C), 10.76 (s, 1H, N-H). Mass analysis (m/z, %): 430 (M^+,^ 10.75), 388 (27.98), 305 (20.75), 149 (60.04), 131 (23.14), 89 (36.53), 85 (24.98), 76 (45.81), 75 (100.00), 74 (36.09), 72 (41.56). Analysis for: C_22_H_18_N_6_O_4_ (430.42): Calculated: C, 61.39; H, 4.22; N, 19.53. Found: C, 61.49; H, 4.20; N, 19.59%.

#### N’-(2-Cyano-3-(4-nitrophenyl) acryloyl)−5-methyl-1-(p-tolyl)−1 H-1,2,3-triazole-4-carbohydrazide (NM-10)

Orange powder (68% yield); m.p. 278–280 °C. IR (ῡ, cm^− 1^): 3325 (N-H), 3048 (= C-H), 2925 (C-H) aliphatic, 2216 (C ≡ N), 1648 (C = O). ^1^H NMR (DMSO-*d*_6_): *δ* (ppm): 2.41 (s, 3 H, -CH_3_), 2.49 (s, 3 H, -CH_3_), 7.42 (d, *J* = 7.50 Hz, 2 H, Ar-H), 7.51 (d, *J* = 7.50 Hz, 2 H, Ar-H), 8.00 (s, 1H, CH = C), 8.11 (d, *J* = 8.50 Hz, 2 H, Ar-H), 8.31 (d, *J* = 8.50 Hz, 2 H, Ar-H), 10.78 (s, 1H, N-H). ^13^C NMR (DMSO-*d*_6_): *δ* (ppm): 9.7, 21.2, 118.5, 124.5 (2 C), 125.6 (3 C), 126.5, 130.5 (4 C), 133.5, 136.4, 139.0, 140.1, 140.5, 140.8, 147.9, 159.6. Mass analysis (m/z, %):431 (M^+,^ 24.72), 377 (36.53), 366 (41.28), 291 (32.08), 263 (30.92), 182 (63.9), 148 (32.42), 135 (48.89), 103 (62.64), 100 (34.72), 85 (100.00), 84 (76.92), 80.52 (33.36). Analysis for: C_21_H_17_N_7_O_4_ (431.41): Calculated: C, 58.47; H, 3.97; N, 22.73. Found: C, 58.54; H, 3.98; N, 22.77%.

#### N’-(2-Imino-2 H-chromene-3-carbonyl)−5-methyl-1-(p-tolyl)−1 H-1,2,3-triazole-4-carbohydrazide (NM-11)

Yellow powder (60% yield); m.p. above 300 °C. IR (ῡ, cm^− 1^): 3138 (N-H), 3067 (= C-H), 1679 and 1658 (C = O). ^1^H NMR (DMSO-*d*_6_): *δ* (ppm): 2.40 (s, 3 H, -CH_3_), 2.43 (s, 3 H, -CH_3_), 6.60 (s, 1H, Ar-H), 7.03 (t, *J* = 7.00 Hz, 1H, Ar-H), 7.13 (d, *J* = 7.50 Hz, 1H, Ar-H), 7.23 (t, *J* = 7.00 Hz, 2 H, Ar-H), 7.41 (d, *J* = 8.00 Hz, 2 H, Ar-H), 7.46 (d, *J* = 8.00 Hz, 2 H, Ar-H), 7.51 (t, *J* = 7.00 Hz, 1H, Ar-H), 7.71 (d, *J* = 6.00 Hz, 1H, Ar-H), 8.05 (s, 1H, CH = C). ^13^C NMR (DMSO-*d*_6_): *δ* (ppm): 9.4, 20.8, 115.7, 116.3, 118.6, 118.9, 124.2, 125.3, 128.8, 129.3, 129.9, 130.2, 132.8, 133.3, 139.4, 139.9, 153.2, 153.3, 156.1, 157.3, 160.0. Mass analysis (m/z, %):402 (M^+,^ 22.61), 365 (23.07), 356 (25.97), 352 (21.29), 351 (83.94), 349 (86.22), 329 (19.06), 274 (36.58), 273 (34.50), 272 (49.35), 271 (19.66), 270 (100.00), 250 (25.05), 207 (38.55). Analysis for: C_21_H_18_N_6_O_3_ (402.41): Calculated: C, 62.68; H, 4.51; N, 20.88. Found: C, 62.77; H, 4.50; N, 20.93%.

### Quantum chemical calculations: geometry optimization and global reactivity analysis

The initial geometries of all synthesized compounds were constructed and energy-minimized using Chem3D 16.0, and the resulting structures were saved in (*.mol) format. Geometry optimizations were subsequently carried out with the Gaussian 09 W software package (https://gaussian.com/)^50^. Density functional theory (DFT) calculations were performed at the B3LYP/6–311 + + G(d, p) level of theory^51^. This functional combine Becke’s three-parameter exchange with the Lee–Yang–Parr correlation, providing a well-established balance between accuracy, efficiency, and computational cost for organic systems^52^. The chosen split-valence triple-zeta basis set (6-311G) offers higher accuracy compared to minimal or double-zeta basis sets (e.g., STO-3G, 6-31G)^53^. Incorporation of polarization functions (d on heavy atoms and p on hydrogens) improves descriptions of molecular geometry and bonding^54^, while the addition of diffuse functions (++) accounts for long-range charge distribution, anionic states, lone pairs, and hydrogen bonding^55^. These features make the B3LYP/6–311 + + G(d, p) method highly suitable for the DFT analysis of organic compounds^56^.

### Antioxidant activity using DPPH assay

Serial dilutions of each sample were prepared in methanol. A 0.135 mM solution of DPPH• in methanol was freshly prepared and mixed in equal volume with each concentration of the sample dilution. Subsequently, 1.0 mL of the DPPH• solution was added to each reaction tube, and the mixtures were incubated at room temperature in the dark for 30 min to prevent photodegradation. The absorbance of the resulting solutions was then measured at 517 nm using a UV–Vis spectrophotometer. The percentage of remaining DPPH was calculated according to Eq. (1).

1 $$\:\% \:{\text{DPPH}}{\text{.}}\:{\text{remaining}} = \frac{{\left[ {{\text{DPPH}}{\text{.}}} \right]\:{\text{T}}}}{{\left[ {{\text{DPPH}}{\text{.}}} \right]{\text{T}} = 0}}\:{\text{x}}\:100$$

Eq.1[58].

The data were fitted to an exponential curve by plotting the percentage of remaining DPPH• against the sample concentration (mg/mL). The half-maximal inhibitory concentration (IC_50_) was defined as the concentration of antioxidant required to reduce the initial DPPH level by 50%. Accordingly, lower IC_50_ values reflect higher radical scavenging efficiency, indicating an inverse correlation between IC_50_ and antioxidant capacity of the tested samples^58^.

### Bacterial culture

The bacterial species were obtained from the Adama Public Health Research and Referral Laboratory Center in Ethiopia, where they were isolated from stool specimens that had been stored and identified using biochemical testing. It is essential to note that no patients were present during the specimen collection. Eosin Methylene Blue (EMB) agar was utilized to culture the species overnight^59^. The efficacy of the compounds was evaluated against the Gram-positive strains *Staphylococcus aureus* (ATCC 6538) and *Bacillus subtilis* (DMS 1088), as well as the Gram-negative species *Klebsiella pneumoniae* (ATCC 10031) and *Enterobacter cloacae* (DMS 30054). A great deal of information about the microbial inoculum was injected into the entire agar surface to produce varying quantities of the artificially generated chemicals. Subsequently, 100 µL of the sample at the required concentration is introduced into the well following the aseptic creation of a 9 mm diameter aperture using a sterile corn borer or tip. Agar plates are thereafter incubated under appropriate conditions according to the test microorganism. The antimicrobial agent inhibits the growth of the examined microbiological strain and diffuses throughout the agar media. The average inhibition zones were compared to the positive control, a disk containing azithromycin at 2 mg/ml, measured with a ruler, and represented in millimeters. DMSO functioned as a negative control for the duration of the experiment. For compounds **NM-1**, **NM-3**, and **NM-4**, a novel approach was utilized, which involved dispersing a volume of the microbial inoculum over the whole surface of the agar. A sterile cork tip or borer is utilized to aseptically create a 9-mm aperture, into which a volume of 50 µL of sample combined with 50 µL of antibiotic is introduced at the appropriate concentration. Subsequently, based on the test microorganism, agar plates are subjected to the appropriate incubation conditions. The antimicrobial agent uniformly distributes throughout the agar medium, inhibiting the growth of the examined microbiological strain. The mean value ± standard deviation was utilized to denote the mean inhibition zone (MIZ)^60,61^.

### Molecular induced fit (flexible) Docking study

The target protein underwent molecular docking studies utilizing E. coli gyrase B (24 kDa) (**PDB: 6YD9**)^62^. X-ray crystallography, with a resolution of 1.60 Å and devoid of known mutations, was employed to elucidate the structure of this protein. A co-crystallized ligand (ON_2_) at the active binding site is integral to the single polypeptide chain (chain A) constituting the protein. The structure was formed by including hydrogen atoms, identifying the binding site, and removing water molecules prior to docking. Docking simulations were restricted to Chain A, specifically targeting the active site comprised of the critical amino acid residues: Arg136, Pro79, Ile94, Asp73, and Gly77. The Molecular Operating Environment (MOE) 2019 software was the instrument used for molecular docking. The MOE 2019 program was utilized to generate the three-dimensional structures of the chemicals being examined. Pose reproduction, a prevalent method that entails re-docking a known ligand into its original binding site to evaluate the transparency of the docking algorithm, originated to validate the reliability of the docking process^63^. This study included re-docking the ligand and co-crystallization (ON_2_) into the binding site of the **6YD9** protein. Visual Molecular Dynamics (VMD) and Discovery Studio Visualizer were employed to compute the resultant RMSD between the docked and crystallographic positions, yielding a value of 1.181 Å. The accuracy and effectiveness of the MOE program in predicting how ligands bind are confirmed because its value is within the acceptable range for docking validation. MOE was deemed suitable for docking the newly synthesized ligands to the precisely same protein target^64^.

## Conclusion

A series of triazole-based carbohydrazide hydrazones (**NM-1 to NM-11**) was efficiently synthesized and characterized. Structural and electronic variations strongly influenced their antioxidant and antibacterial activities. Compounds bearing electron-withdrawing substituents, particularly **NM-7**,** NM-8**, and **NM-10**, exhibited the highest biological efficacy, supported by DFT and molecular docking results showing favorable charge transfer and strong enzyme binding. Overall, the findings demonstrate that electronic modulation within the hydrazone framework can effectively tune biological reactivity. These scaffolds represent promising leads for the development of multifunctional agents with combined antimicrobial and antioxidant potential. Future studies will focus on pharmacokinetic optimization and in vivo evaluation to advance these candidates toward therapeutic applications.

## Supplementary Information

Below is the link to the electronic supplementary material.

Supplementary Material 1

## Data Availability

All data generated or analyzed during this study are included in this published article and its supplementary information files.

## Declarations

### Dual publication

The results/data/figures in this manuscript have not been published elsewhere, nor are they under consideration (from you or one of your Contributing Authors) by another publisher.

### Third party material

All of the material is owned by the authors and/or no permissions are required.
