# Supplementary material for: Novel Triazole-Carbohydrazide hydrazones with dual antioxidant and antibacterial potential
Source: Sci Rep. 2025 Nov 21;15:41456. doi: 10.1038/s41598-025-26016-x (PMC12644544; doi:10.1038/s41598-025-26016-x)
Supplement: Supplementary file 1 — Supplementary Material 1 [file 41598_2025_26016_MOESM1_ESM.docx]

**Table (S1)**: presents the antioxidant evaluation results for the studied compounds (**NM-1** to **NM-11**), including their **IC_50_** (mg/mL), percentage of remaining DPPH, and percentage of free radical scavenging activity

| **Sample** | **Concentrations (mg/mL)** | **%Remaining DPPH** | **%Scavenging activity** | **IC_50_ (mg/mL)** |  |
| --- | --- | --- | --- | --- | --- |
| **NM-1** | 1.84 | 34.75±0.9 | 65.25±0.9 | **1.16±0.93** |  |
|  | 0.92 | 54.58 ±1.01 | 45.42±1.01 |  |  |
|  | 0.46 | 76.69±1.04 | 23.31±1.04 |  |  |
|  | 0.23 | 84.53±0.8 | 15.47±0.8 |  |  |
| **NM-2** | 0.057 | 52.92± 1.02 | 47.08±1.02 | **3.55±0.96** |  |
|  | 0.028 | 69.79±0.7 | 30.21±0.7 |  |  |
|  | 0.014 | 75.63 ±1.03 | 24.37±1.03 |  |  |
|  | 0.007 | 78.28±1.09 | 21.72±1.09 |  |  |
| **NM-3** | 3.64 | 59.91±1.11 | 40.09±1.11 | **4.75±0.88** |  |
|  | 1.82 | 68.63±0.8 | 31.37±0.8 |  |  |
|  | 0.91 | 81.7±1.03 | 18.3±1.03 |  |  |
|  | 0.455 | 93.25 ±0.6 | 6.754±0.6 |  |  |
| **NM-4** | 2.5 | 51.49 ± 0.09 | 48.51±0.09 | **2.58±0.46** |  |
|  | 1.25 | 71.84± 0.4 | 28.16±0.4 |  |  |
|  | 0.625 | 85.17 ± 0.65 | 14.83±0.65 |  |  |
|  | 0.313 | 95.63 ±0.70 | 4.368±0.70 |  |  |
| **NM-5** | 0.109 | 55.67±0.05 | 44.33±0.05 | **0.12±0.06** |  |
|  | 0.055 | 61.31±0.09 | 38.69±0.09 |  |  |
|  | 0.027 | 83.86±0.06 | 16.14±0.06 |  |  |
|  | 0.014 | 89.5±0.04 | 10.5±0.04 |  |  |
| **NM-6** | 0.444 | 42.19± 0.08 | 57.81±0.08 | **0.33±0.13** |  |
|  | 0.222 | 56.41±0.1 | 43.59±0.1 |  |  |
|  | 0.111 | 76.09 ±0.12 | 23.91±0.12 |  |  |
|  | 0.055 | 92.19±0.22 | 7.81±0.22 |  |  |
| **5** | 2.663 | 26.07±0.11 | 73.93±0.11 | **0.83±0.58** |  |
|  | 1.331 | 42.42± 0.32 | 57.58±0.32 |  |  |
|  | 0.666 | 49.89±0.90 | 50.11±0.90 |  |  |
|  | 0.333 | 62.44±1.01 | 37.56±1.01 |  |  |
| **NM-7** | 0.146 | 26.25±0.05 | 73.75±0.05 | **0.04±0.05** |  |
|  | 0.073 | 41.29±0.02 | 58.71±0.02 |  |  |
|  | 0.037 | 46.84±0.08 | 53.16±0.08 |  |  |
|  | 0.018 | 66.78 ±0.07 | 33.22±0.07 |  |  |
| **NM-8** | 0.285 | 19.93±0.01 | 80.07±0.01 | **0.09±0.05** |  |
|  | 0.143 | 27.12±0.02 | 72.88±0.02 |  |  |
|  | 0.071 | 57.63±0.06 | 42.37±0.06 |  |  |
|  | 0.036 | 78.43±0.11 | 21.57±0.11 |  |  |
| **NM-9** | 0.249 | 33.88±0.08 | 66.12±0.08 | **0.13±0.08** |  |
|  | 0.124 | 46.41±0.09 | 53.59±0.09 |  |  |
|  | 0.062 | 66.01±0.06 | 33.99±0.06 |  |  |
|  | 0.031 | 82.9±0.11 | 17.1±0.11 |  |  |
| **NM-10** | 3.333 | 52.9±0.08 | 85.67±0.08 | **0.06±0.07** | |
|  | 1.667 | 59.5 ±0.11 | 72.44±0.11 |  |  |
|  | 0.833 | 68.5 ±0.07 | 59.21±0.07 |  |  |
|  | 0.417 | 78.3 ±0.05 | 45.98±0.05 |  |  |
| **NM-11** | 0.222 | 28.75 ±1.01 | 71.25±0.01 | **0.12±0.19** | |
|  | 0.111 | 56.09 ±0.03 | 43.91±0.03 |  |  |
|  | 0.055 | 76.25±0.07 | 23.75±0.07 |  |  |
|  | 0.028 | 90.31±0.08 | 9.68±0.08 |  |  |
| **Ascorbic acid** | 0.06 | 15.3±0.01 | 84.7 ±0.01 | **0.02±0.03** | |
|  | 0.03 | 39.1±0.05 | 60.9 ±0.05 |  |  |
|  | 0.02 | 61.1 ±0.03 | 38.9 ±0.03 |  |  |
|  | 0.01 | 74.8 ±0.06 | 25.2 ±0.06 |  |  |

**
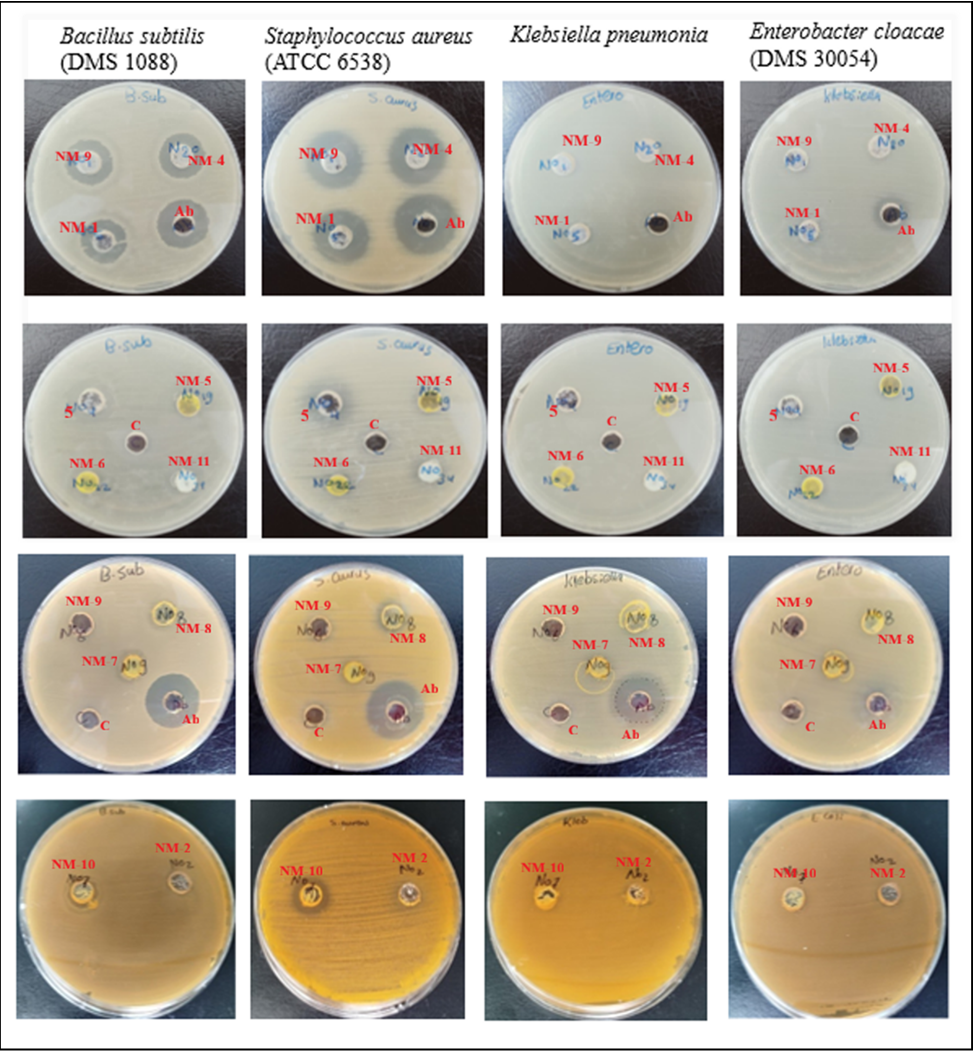
**

**Figure (S2). Antibacterial activity of synthesized triazole-carbohydrazide derivatives (NM-1 to NM-11 and compound 5) demonstrated by agar well diffusion assay. Representative inhibition zones are shown for each compound. ‘C’ denotes the negative control (DMSO), and ‘Ab’ denotes the positive control (standard antibiotic).**


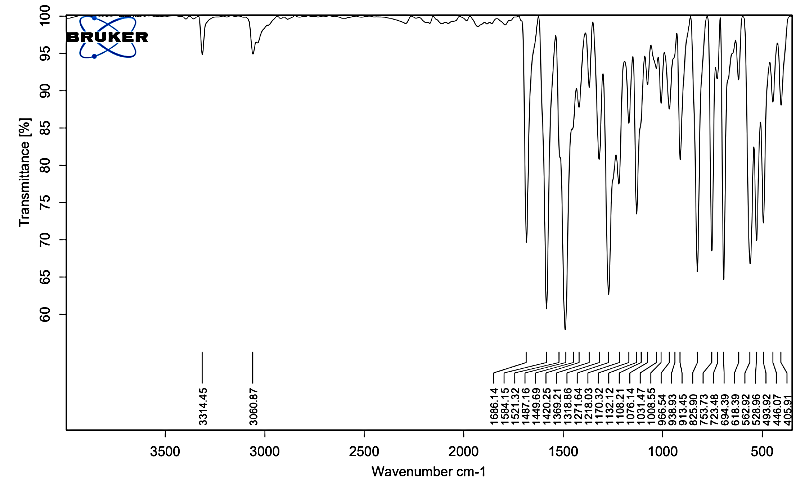

**Fig (S3): IR spectrum of compound NM-1.**

**
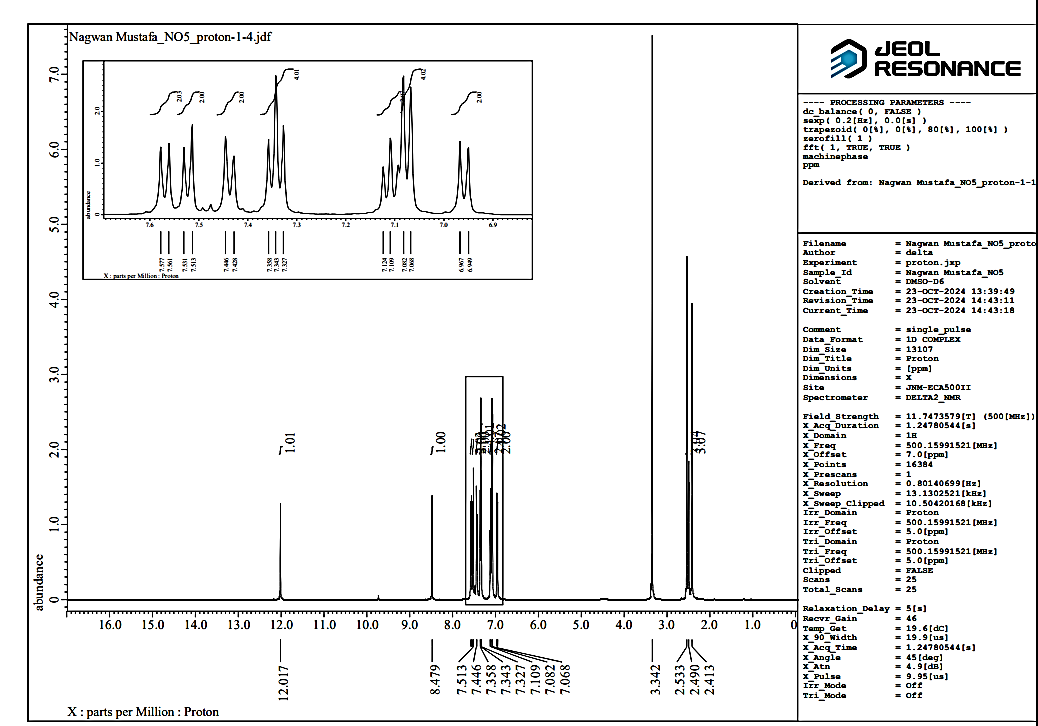
**

**Fig (S4): ^1^H NMR spectrum of compound NM-1.**

**
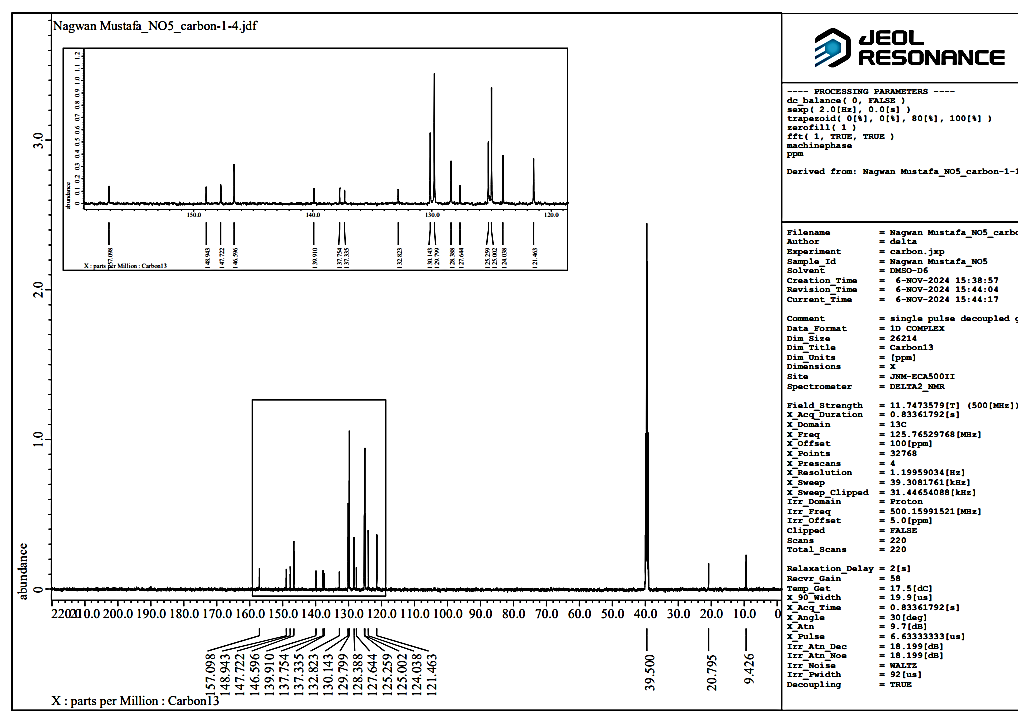
**

**Fig (S5): ^13^C NMR spectrum of compound NM-1.**

**Fig (S6): Mass spectroscopy of compound NM-1.**


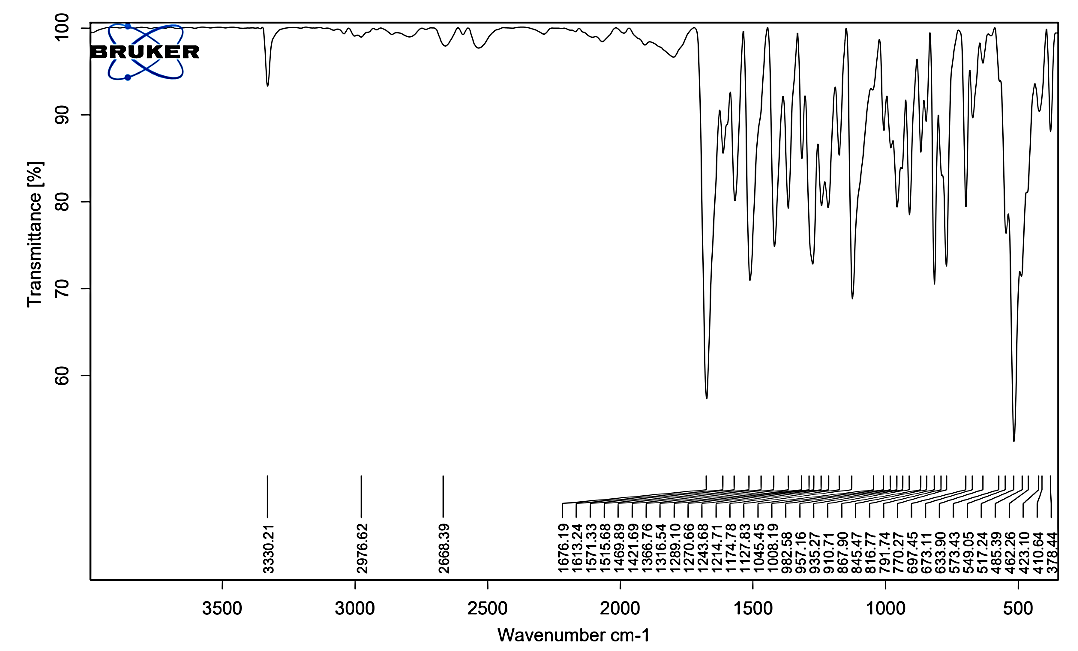


**Fig (S7): IR spectrum of compound NM-2.**

**
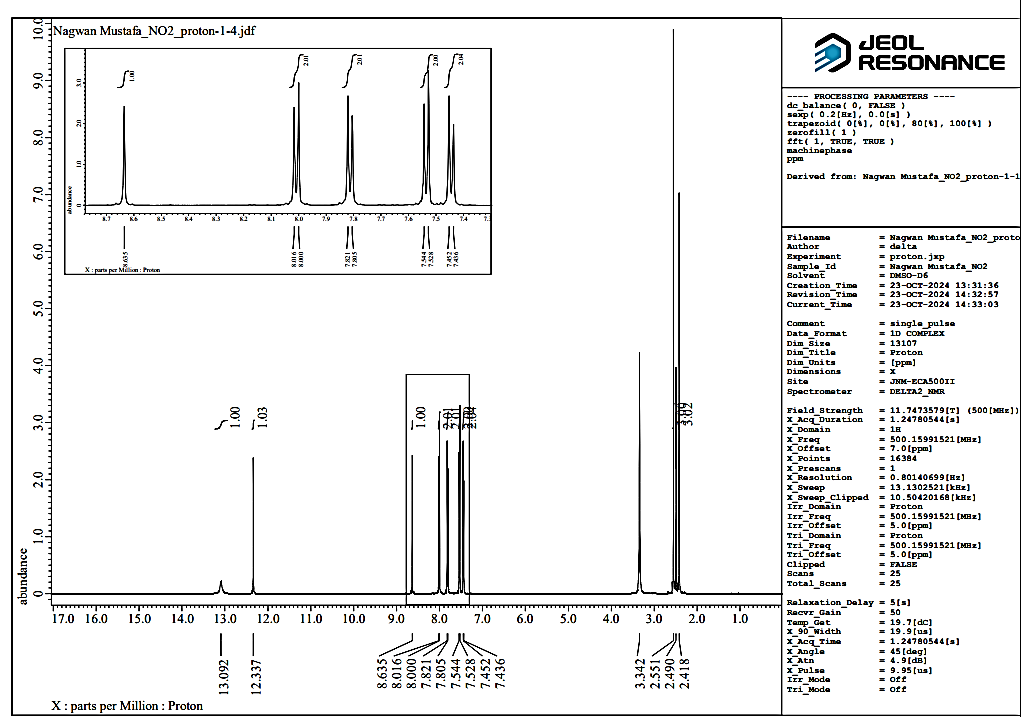
**

**Fig (S8): ^1^H NMR spectrum of compound NM-2.**

**
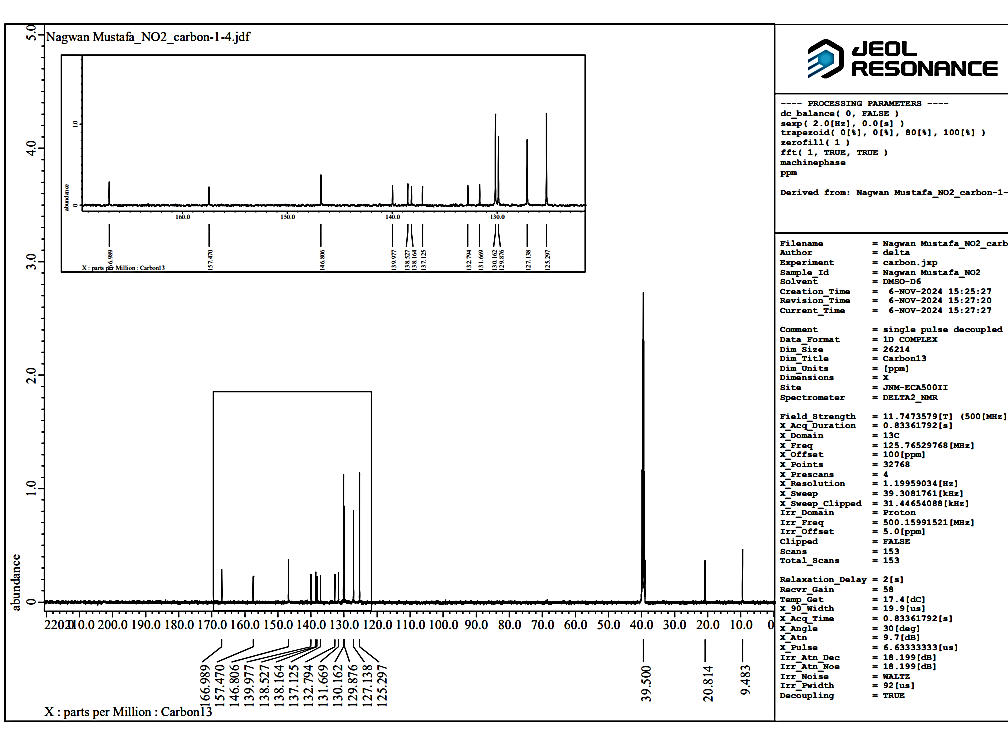
**

**Fig (S9): ^13^C NMR spectrum of compound NM-2.**

**Fig (S10): Mass spectroscopy of compound NM-2.**


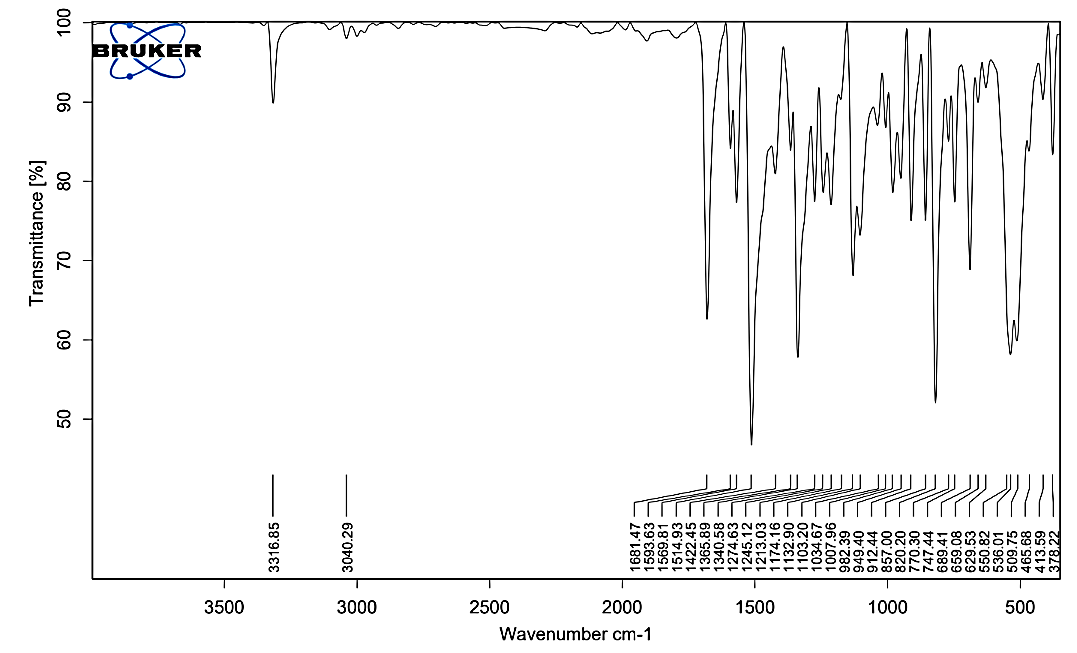


**Fig (S11): IR spectrum of compound NM-3.**

**
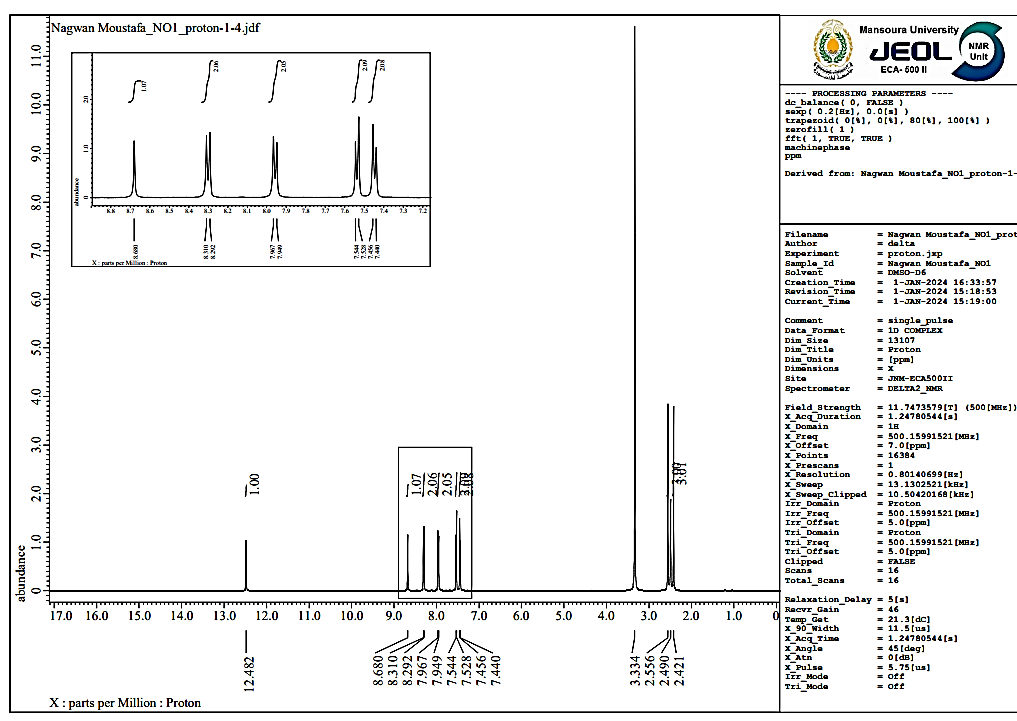
**

**Fig (S12): ^1^H NMR spectrum of compound NM-3.**

**
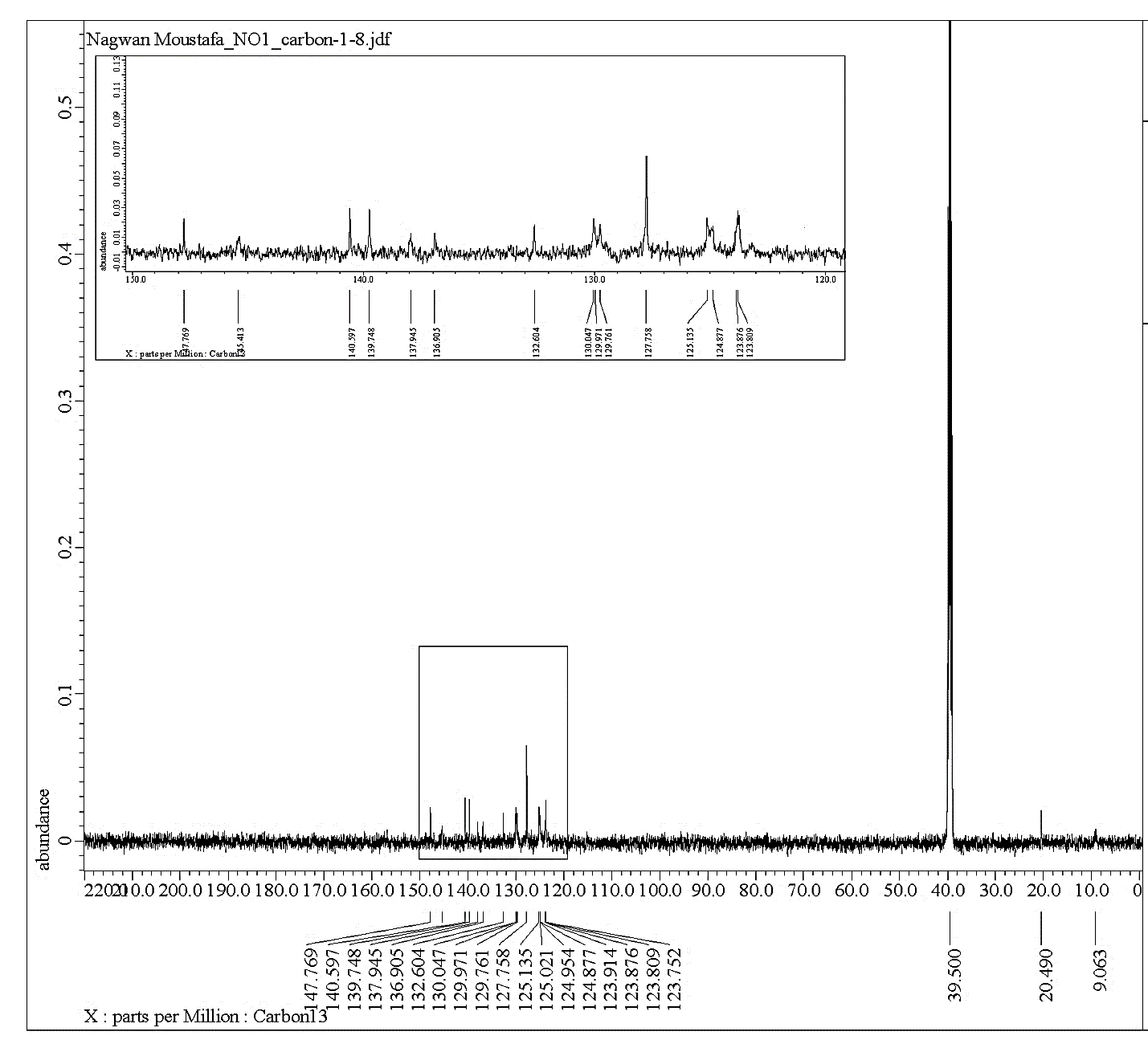
**

**Fig (S13): ^13^C NMR spectrum of compound NM-3.**

**Fig (S14): Mass spectroscopy of compound NM-3.**


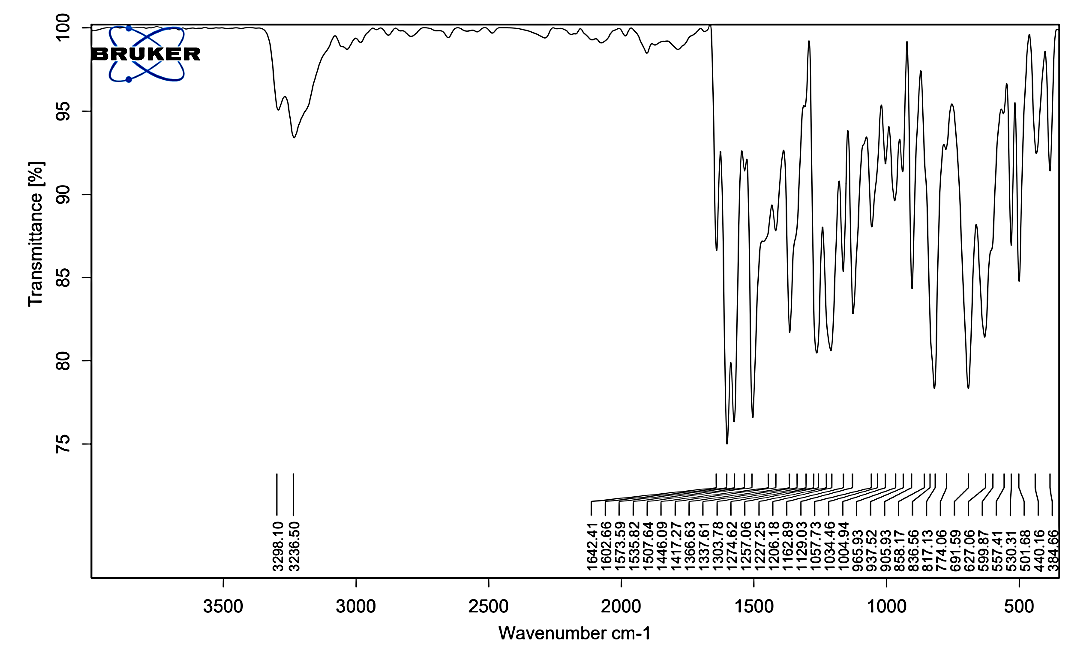


**Fig (S15): IR spectrum of compound NM-4.**

**
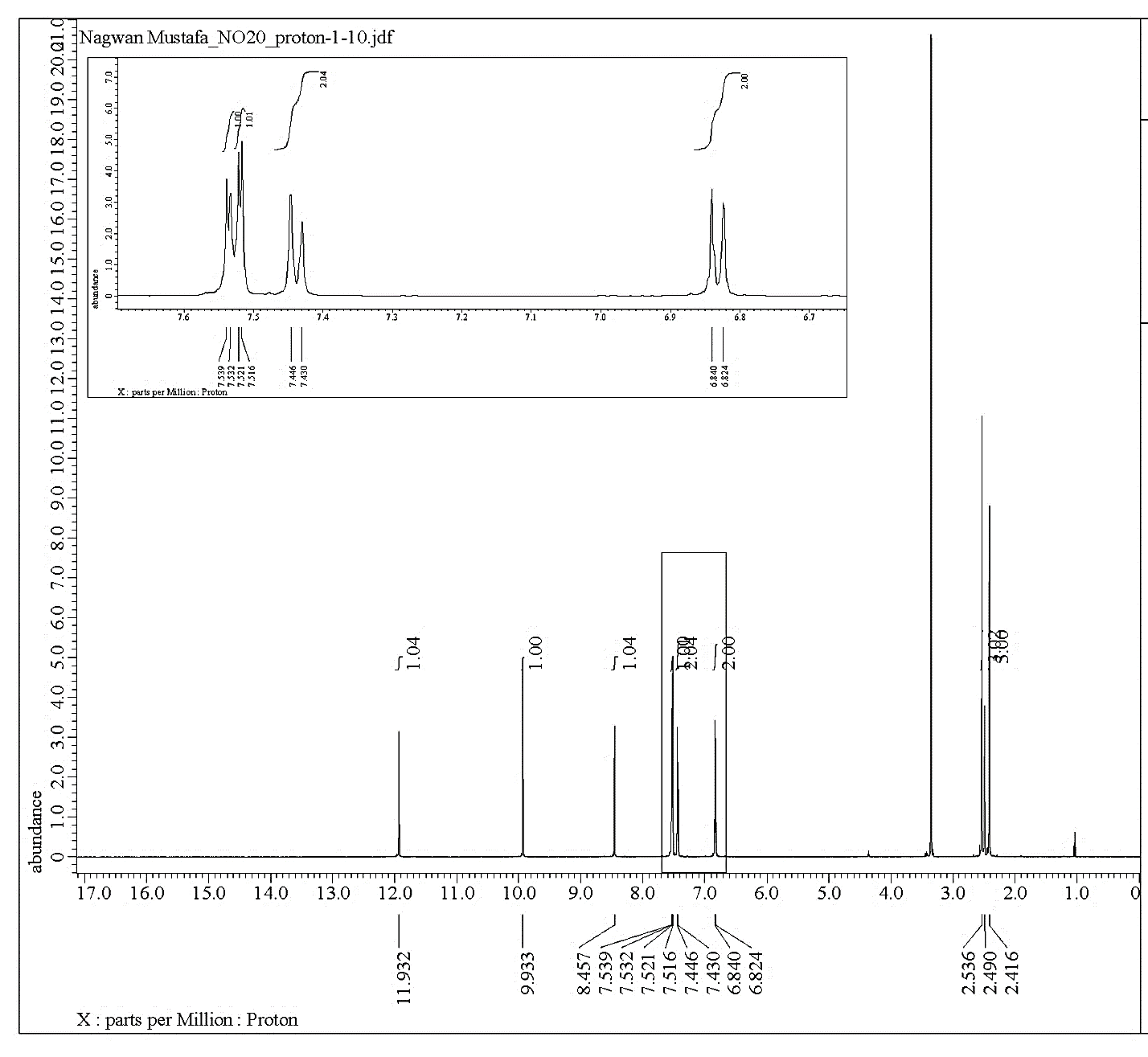
**

**Fig (S16): ^1^H NMR spectrum of compound NM-4.**


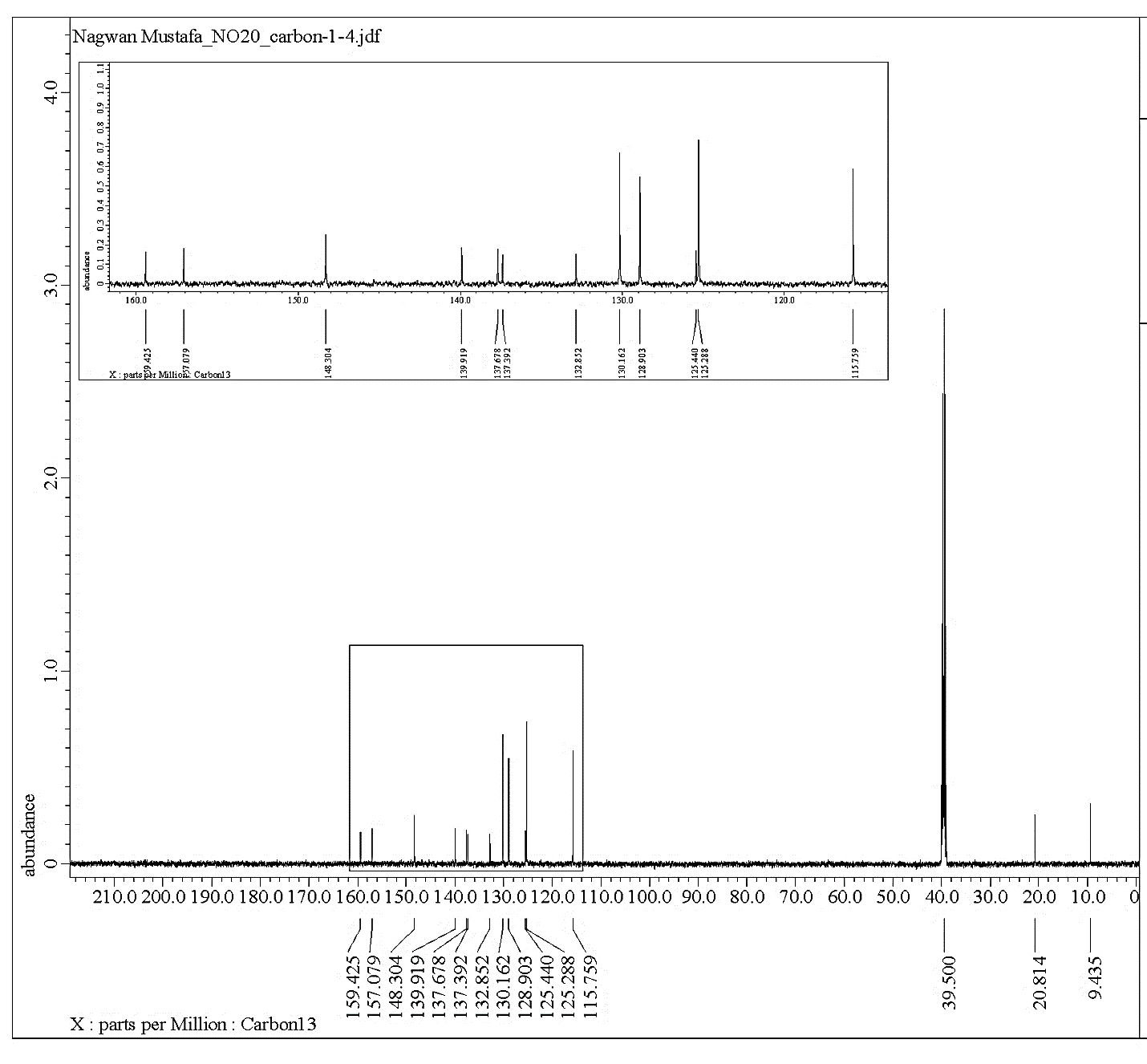

**Fig (S17): ^13^C NMR spectrum of compound NM-4.**

**Fig (S18): Mass spectroscopy of compound NM-4.**


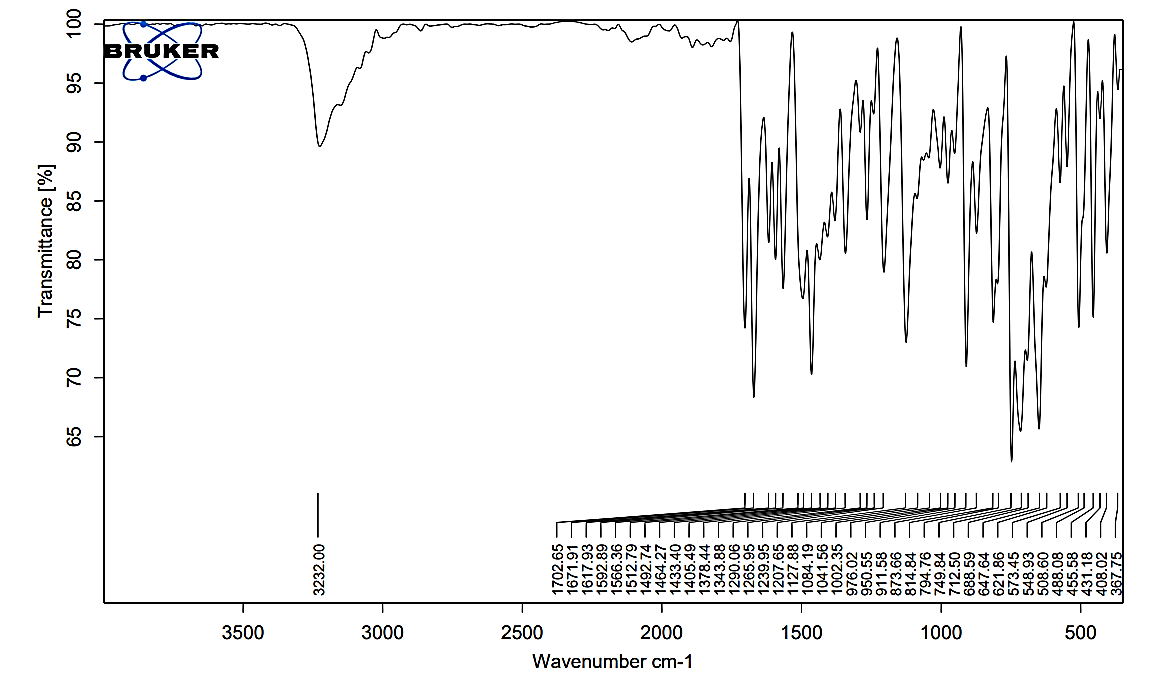


**Fig (S19): IR spectrum of compound NM-5.**

**
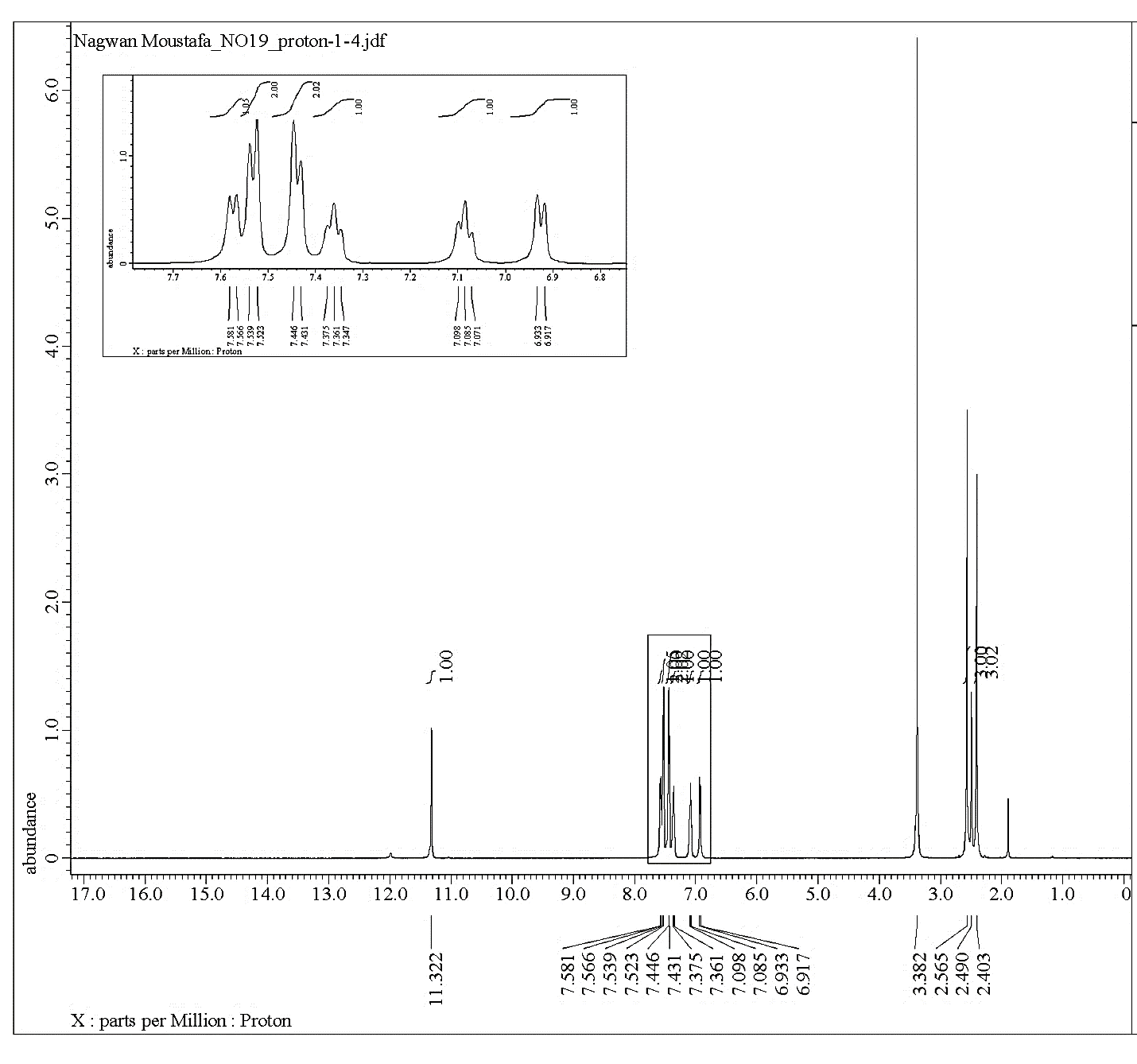
**

**Fig (S20): ^1^H NMR spectrum of compound NM-5.**

**
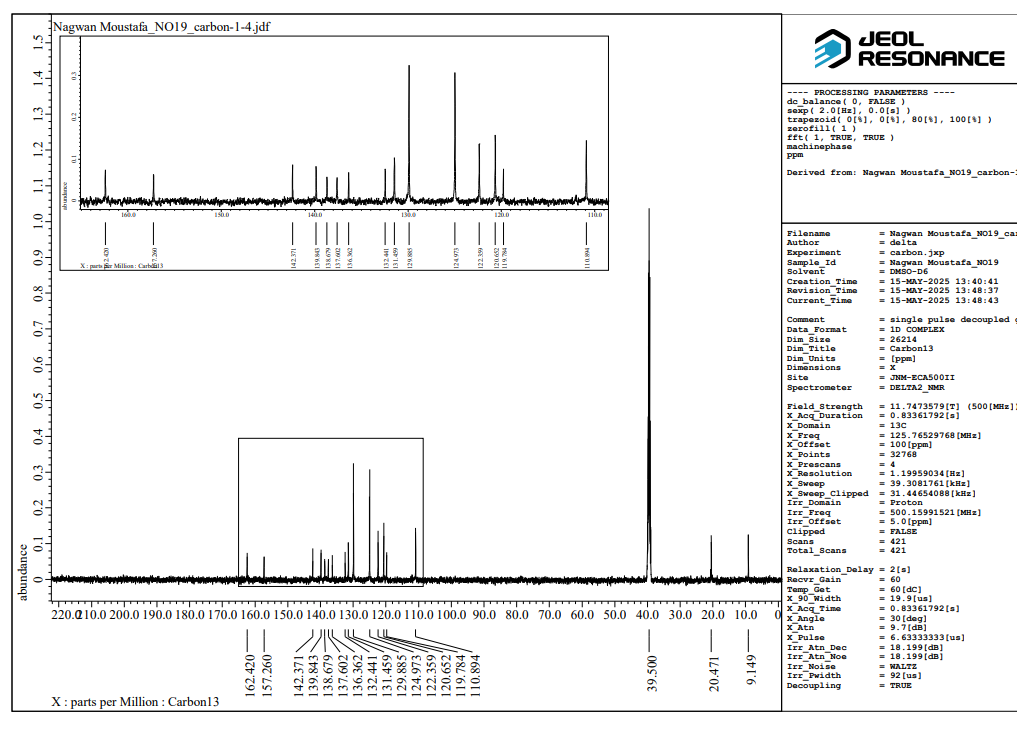
**

**Fig (S21): ^13^C NMR spectrum of compound NM-5.**


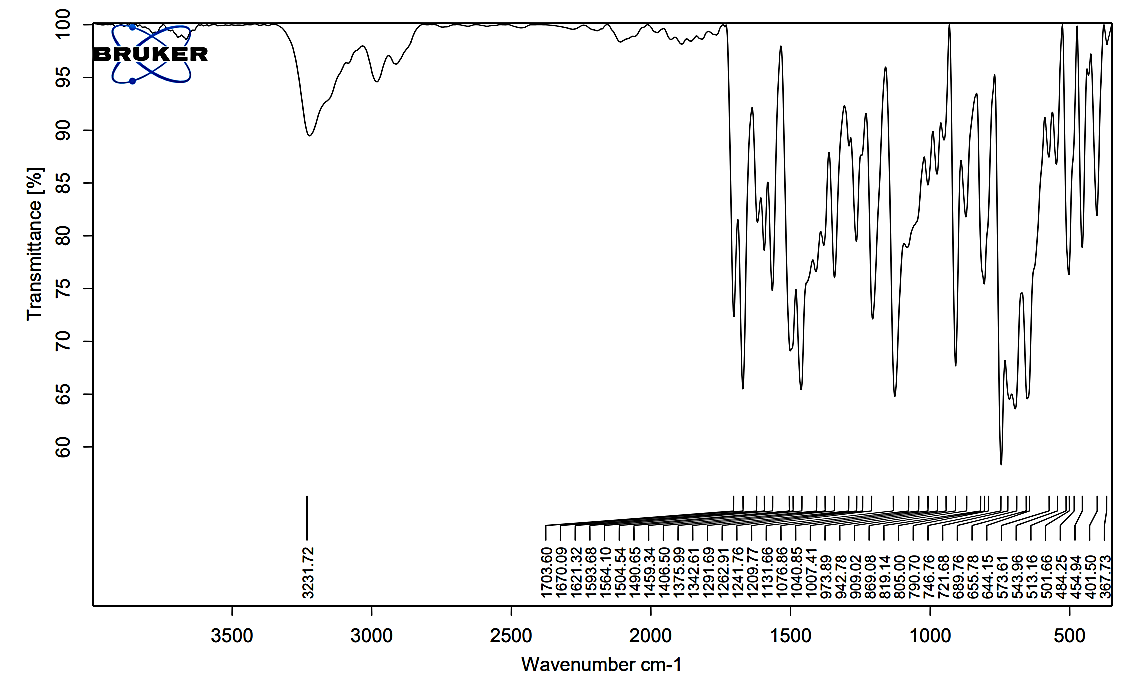

**Fig (S22): IR spectrum of compound NM-6.**


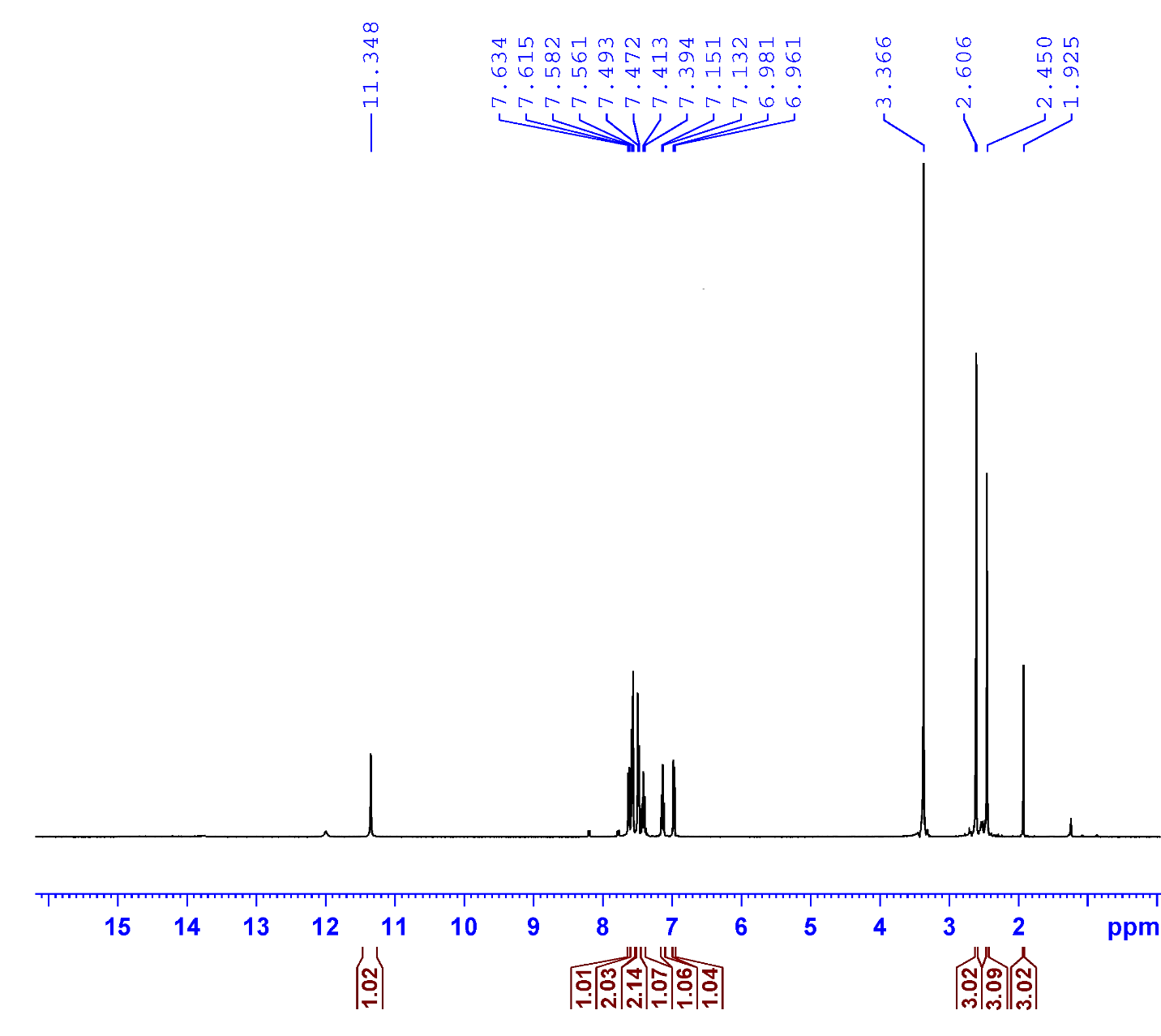

**Fig (S23): ^1^H NMR spectrum of compound NM-6.**


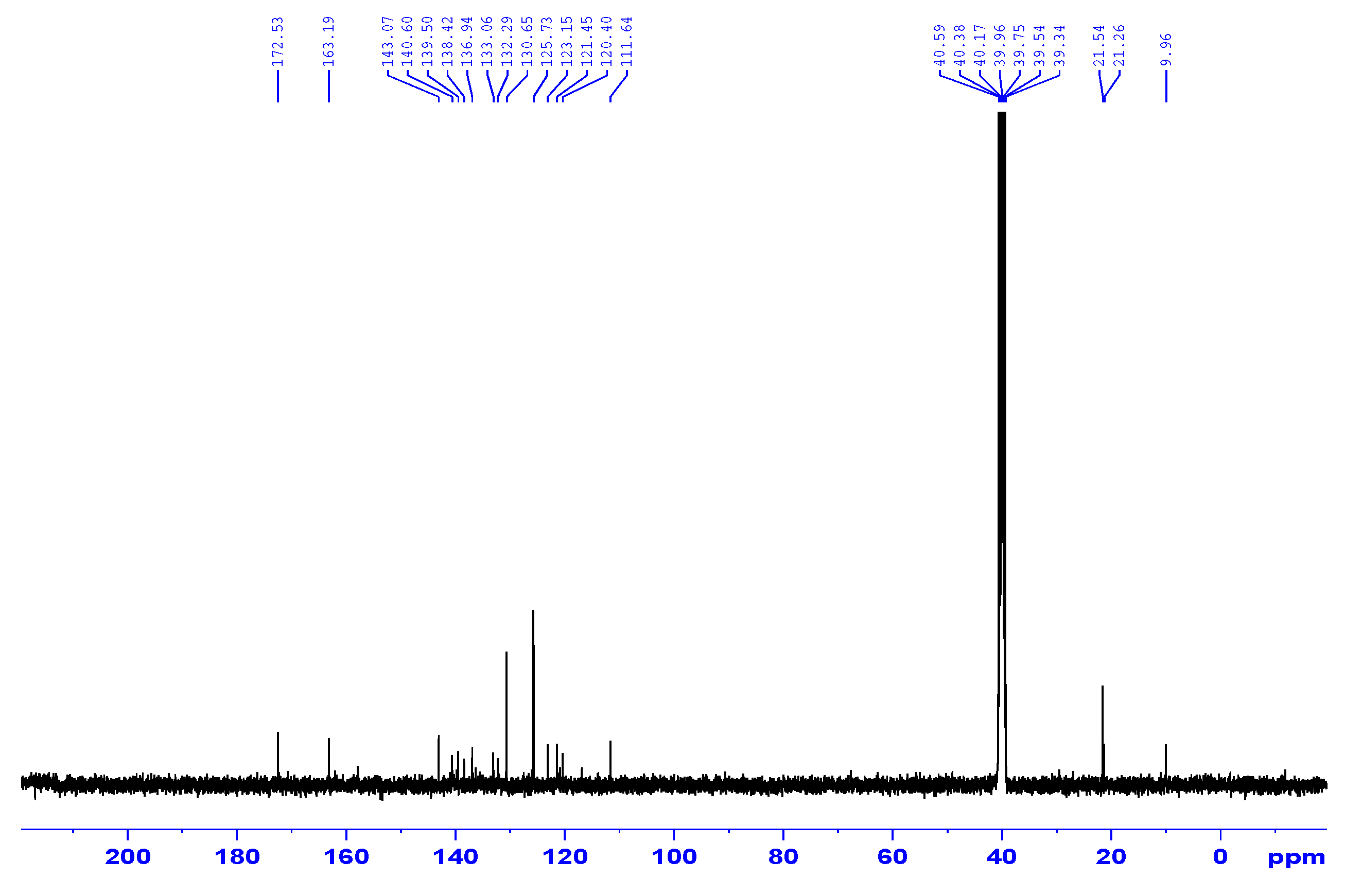

**Fig (S24): ^13^C NMR spectrum of compound NM-6.**

**Fig (S25): Mass spectroscopy of compound NM-6.**


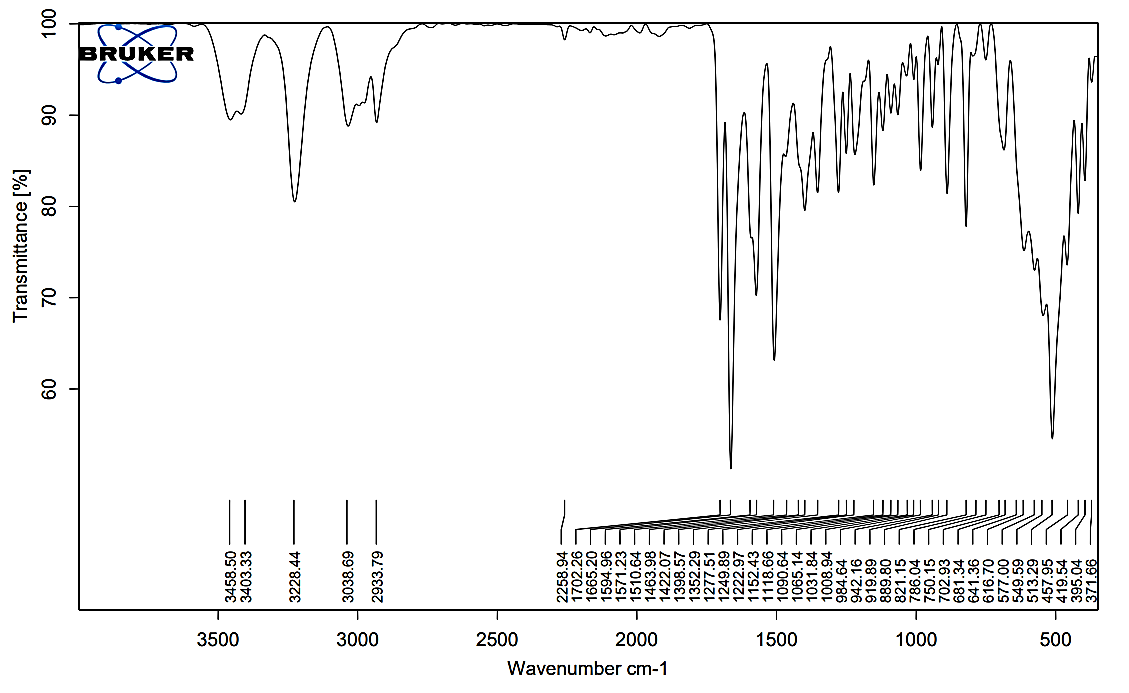

**Fig (S26): IR spectrum of compound 5.**


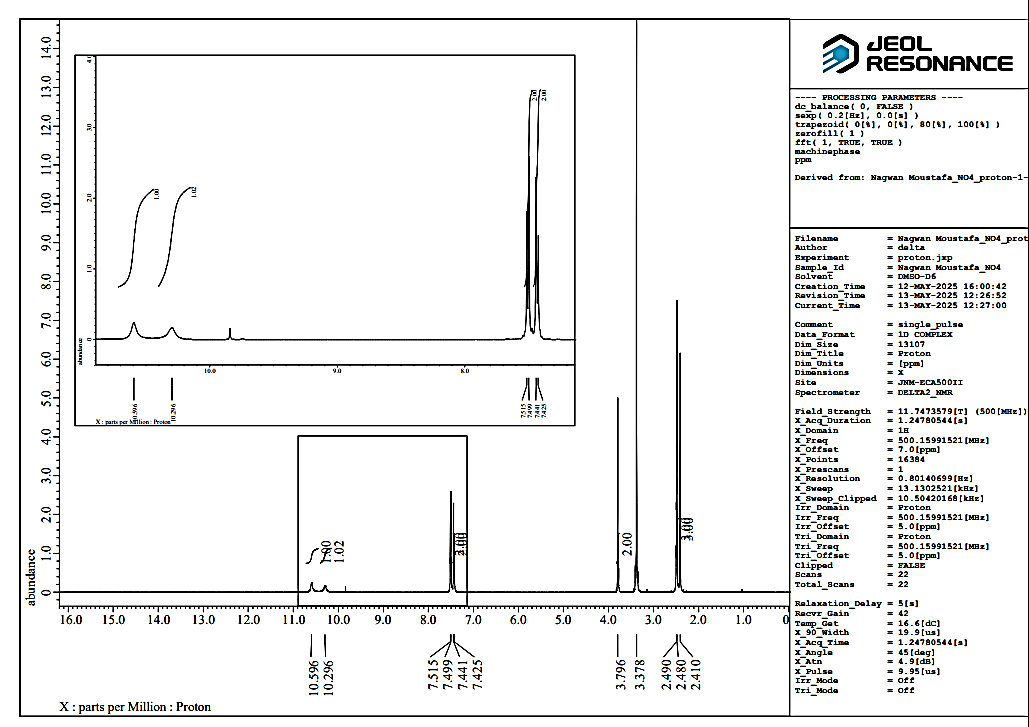

**Fig (S27): ^1^H NMR spectrum of compound 5.**


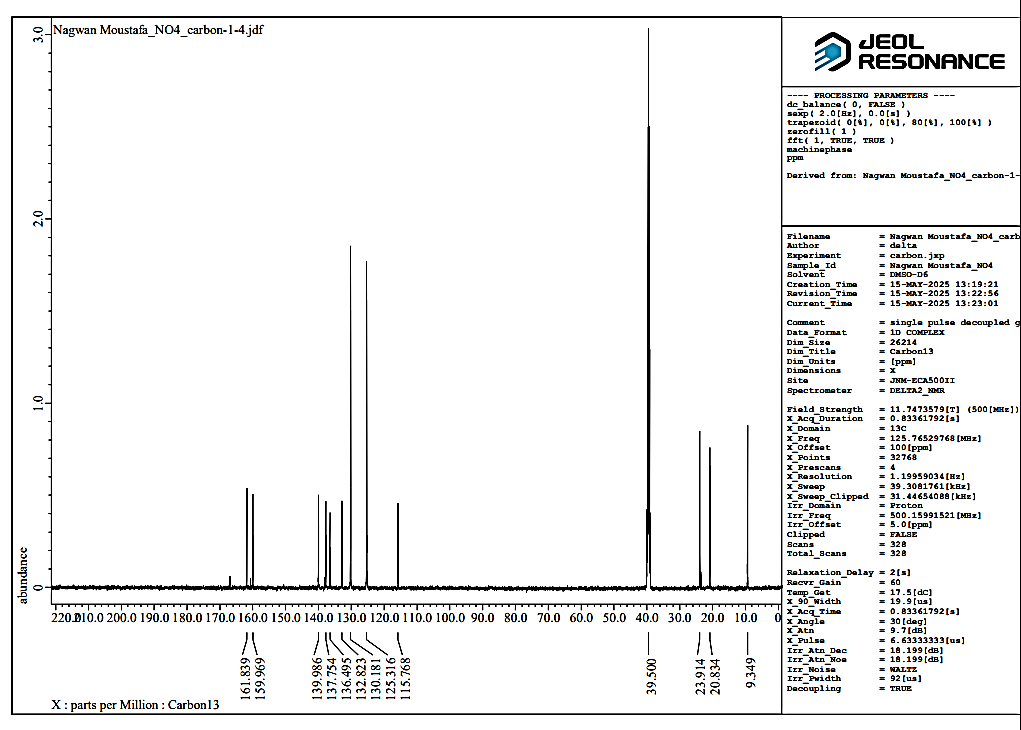

**Fig (S28): ^13^C NMR spectrum of compound 5.**


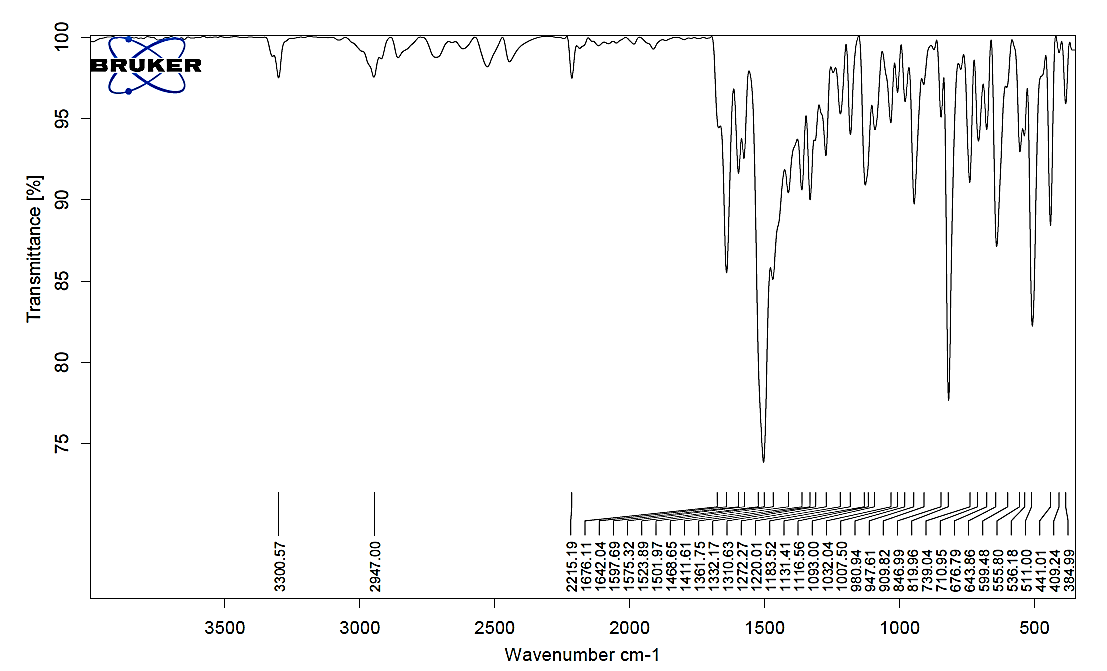


**Fig (S29): IR spectrum of compound NM-7.**

**
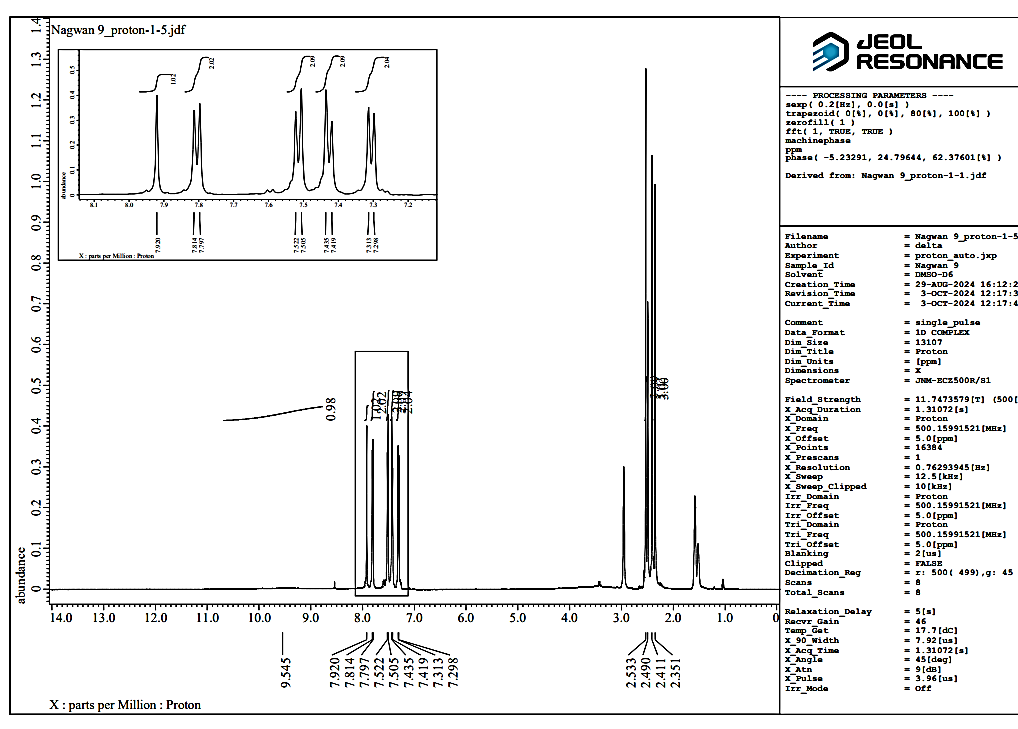
**

**Fig (S30): ^1^H NMR spectrum of compound NM-7.**


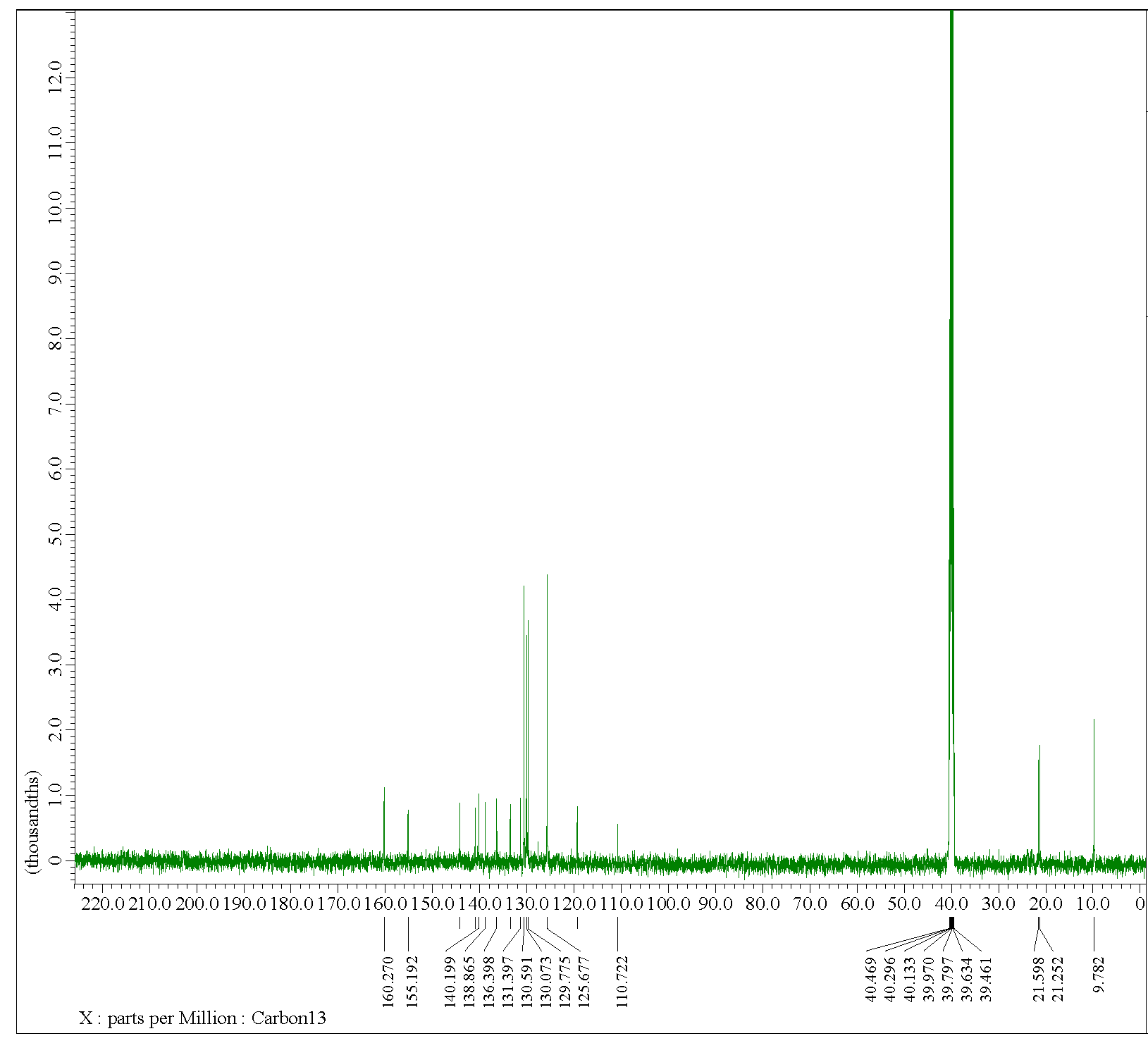

**Fig (S31): ^13^C NMR spectrum of compound NM-7.**

**Fig (S32): Mass spectroscopy of compound NM-7.**


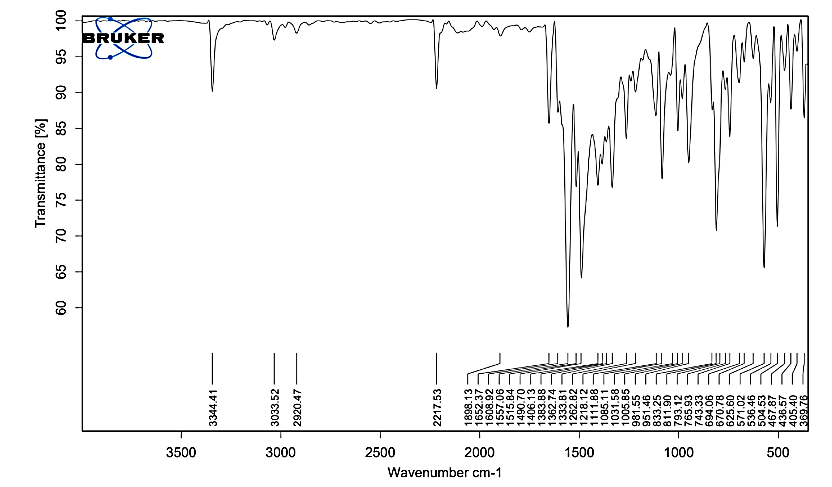


**Fig (S33): IR spectrum of compound NM-8.**

**
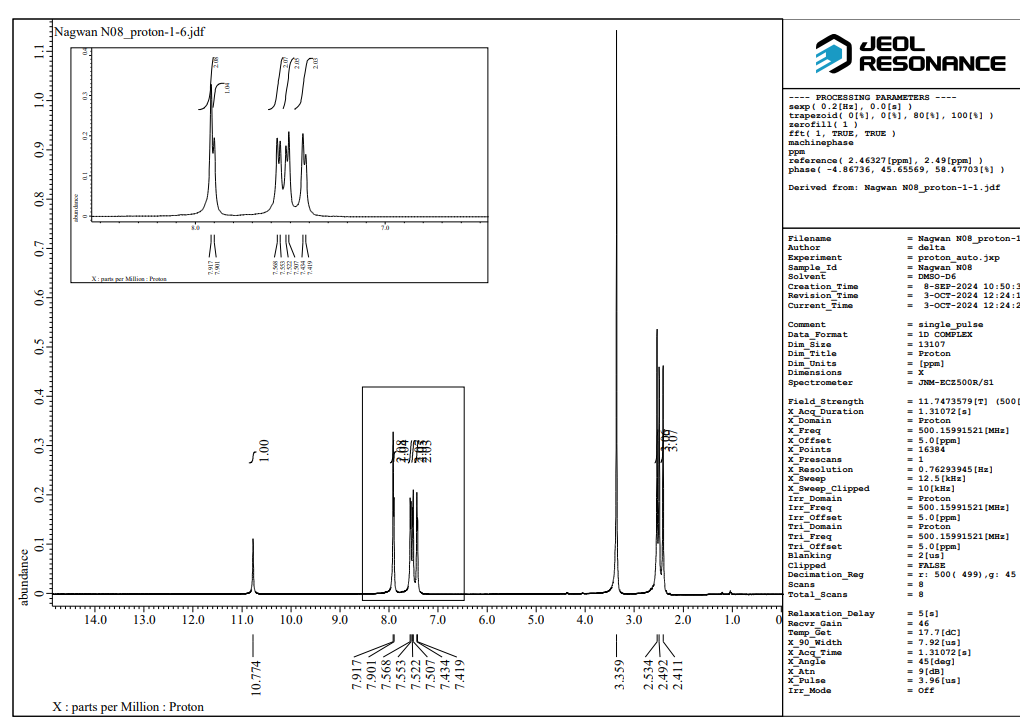
**

**Fig (S34): ^1^H NMR spectrum of compound NM-8.**

**
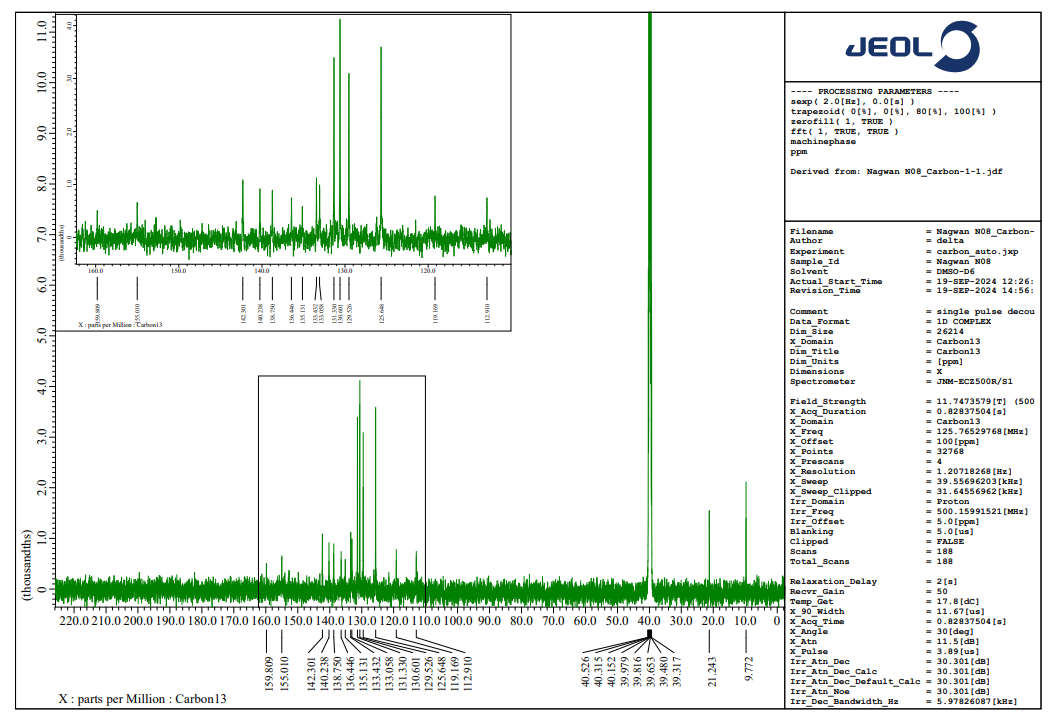
**

**Fig (S35): ^13^C NMR spectrum of compound NM-8.**

**Fig (S36): Mass spectroscopy of compound NM-8.**


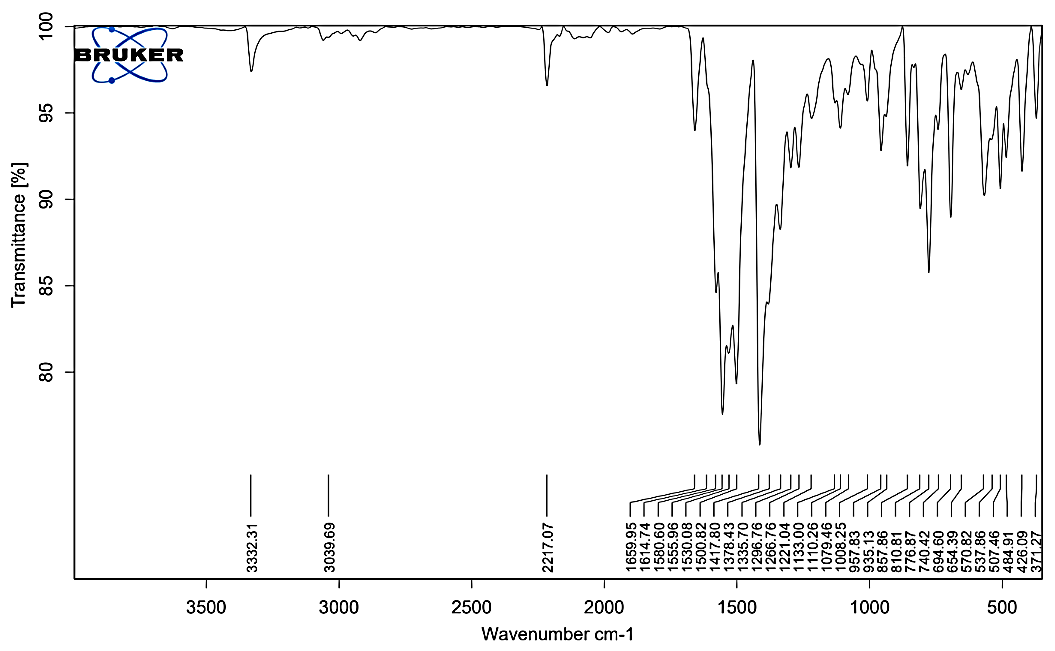


**Fig (S37): IR spectrum of compound NM-9.**

**
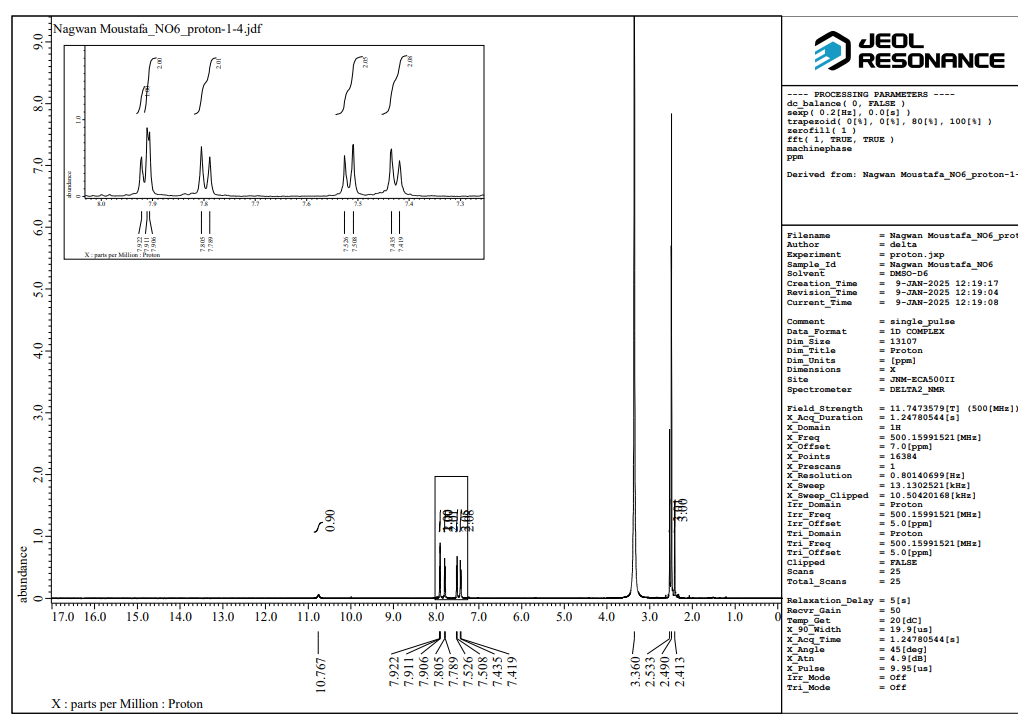
**

**Fig (S38): ^1^H NMR spectrum of compound NM-9.**


**Fig (S39): Mass spectroscopy of compound NM-9.**


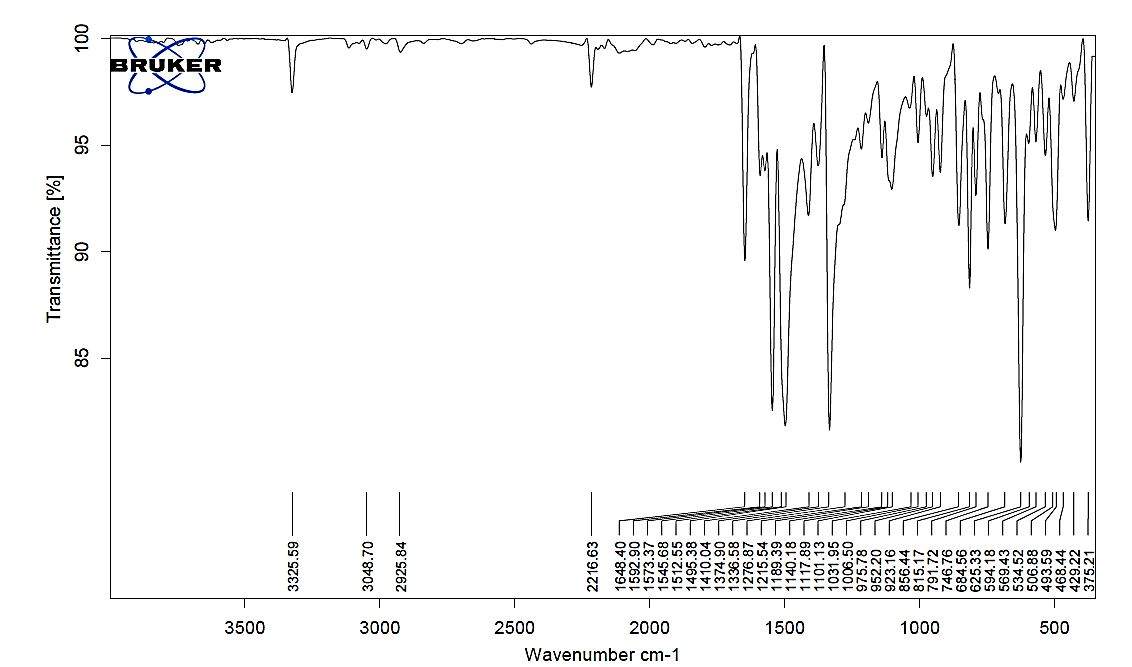


**Fig (S40): IR spectrum of compound NM-10.**

**
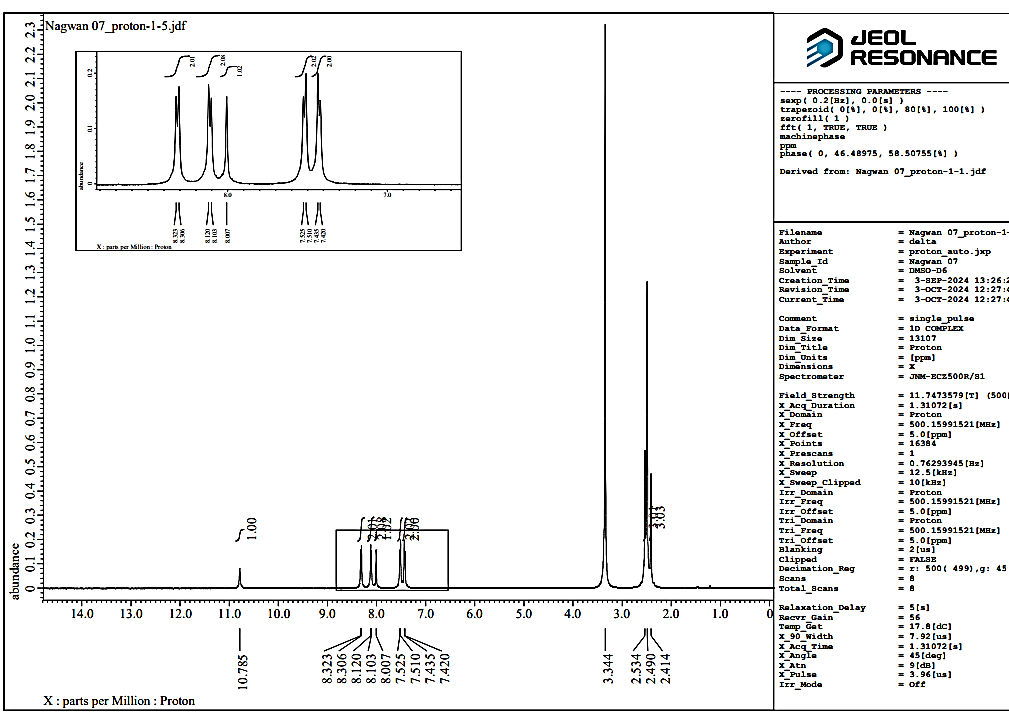
**

**Fig (S41): ^1^H NMR spectrum of compound NM-10.**


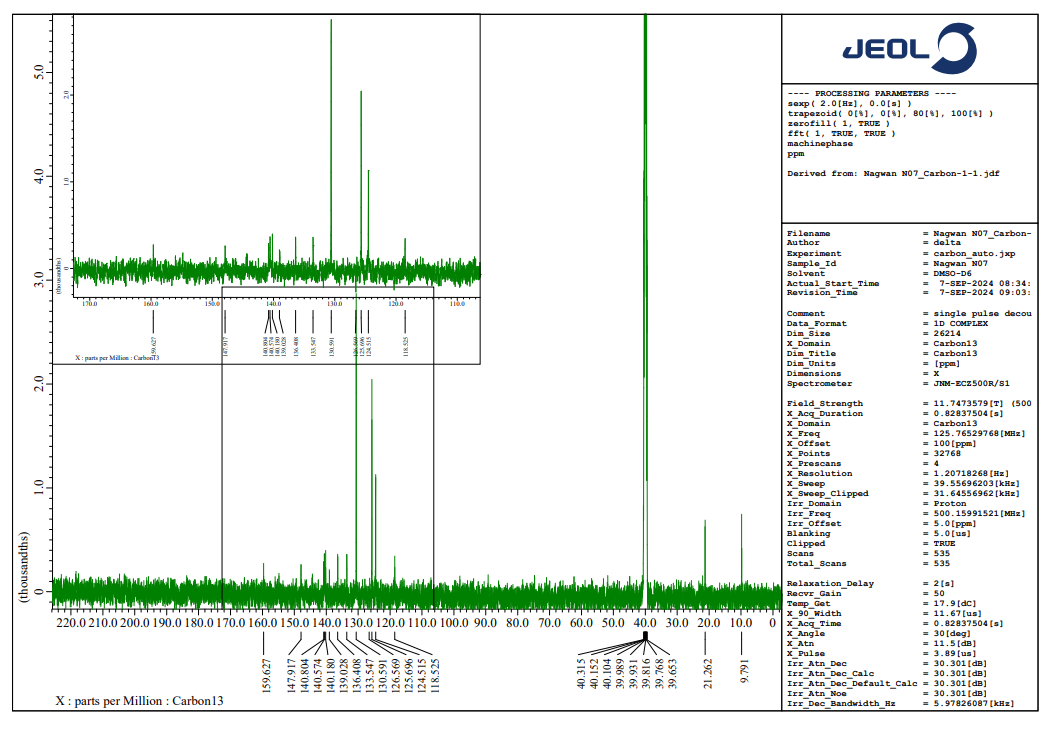

**Fig (S42): ^13^C NMR spectrum of compound NM-10.**

**Fig (S43): Mass spectroscopy of compound NM-10.**


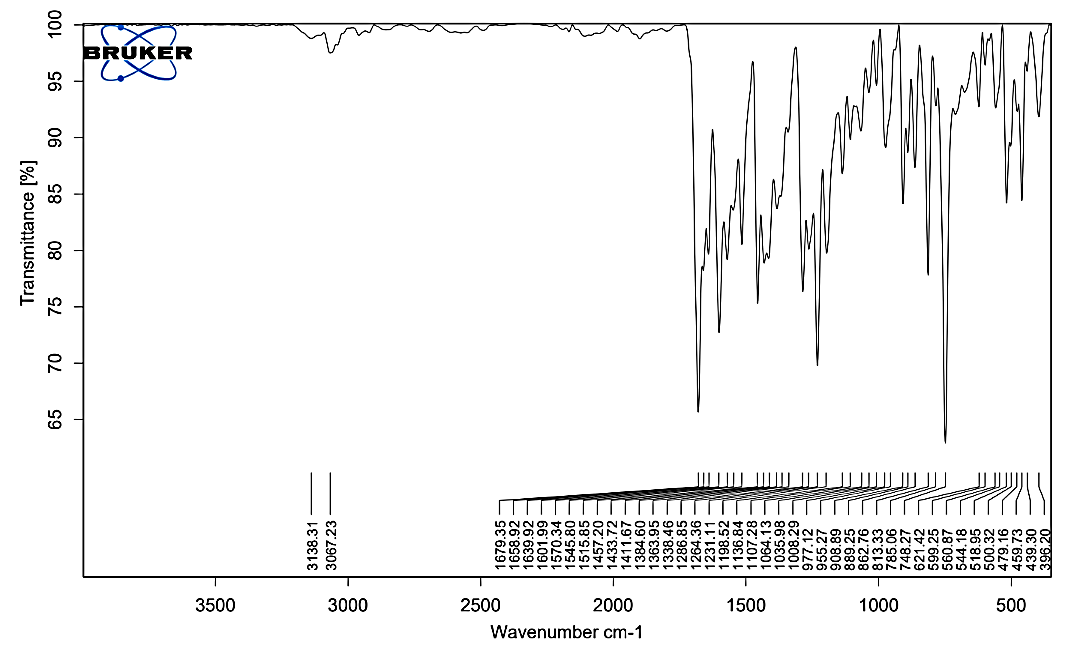

**Fig (S44): IR spectrum of compound NM-11.**


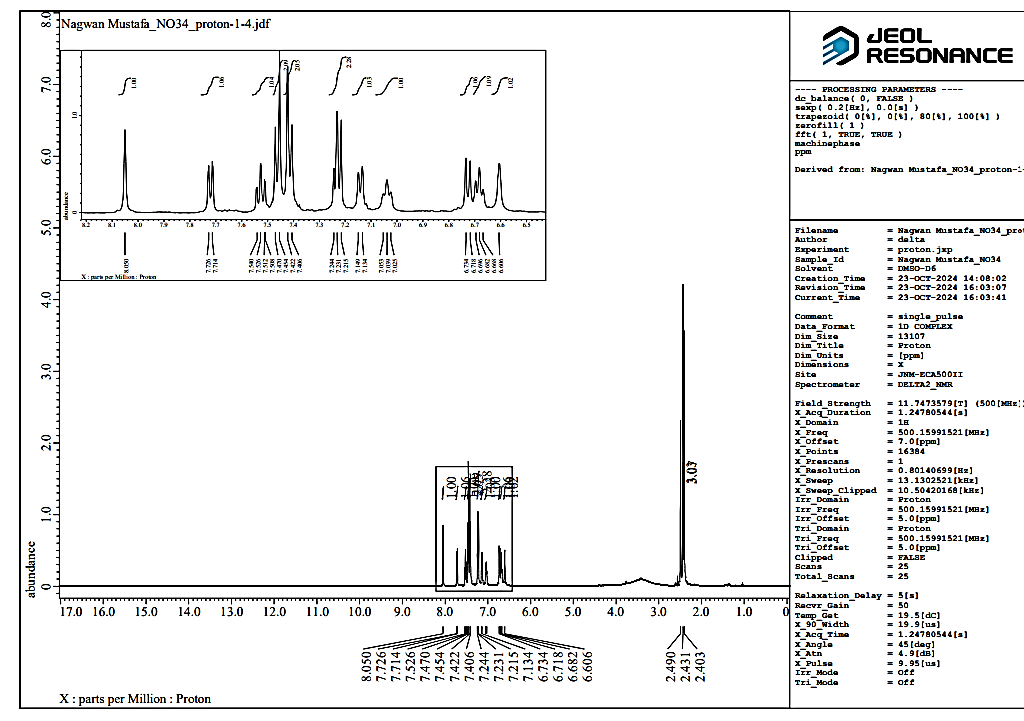


**Fig (S45): ^1^H NMR spectrum of compound NM-11.**


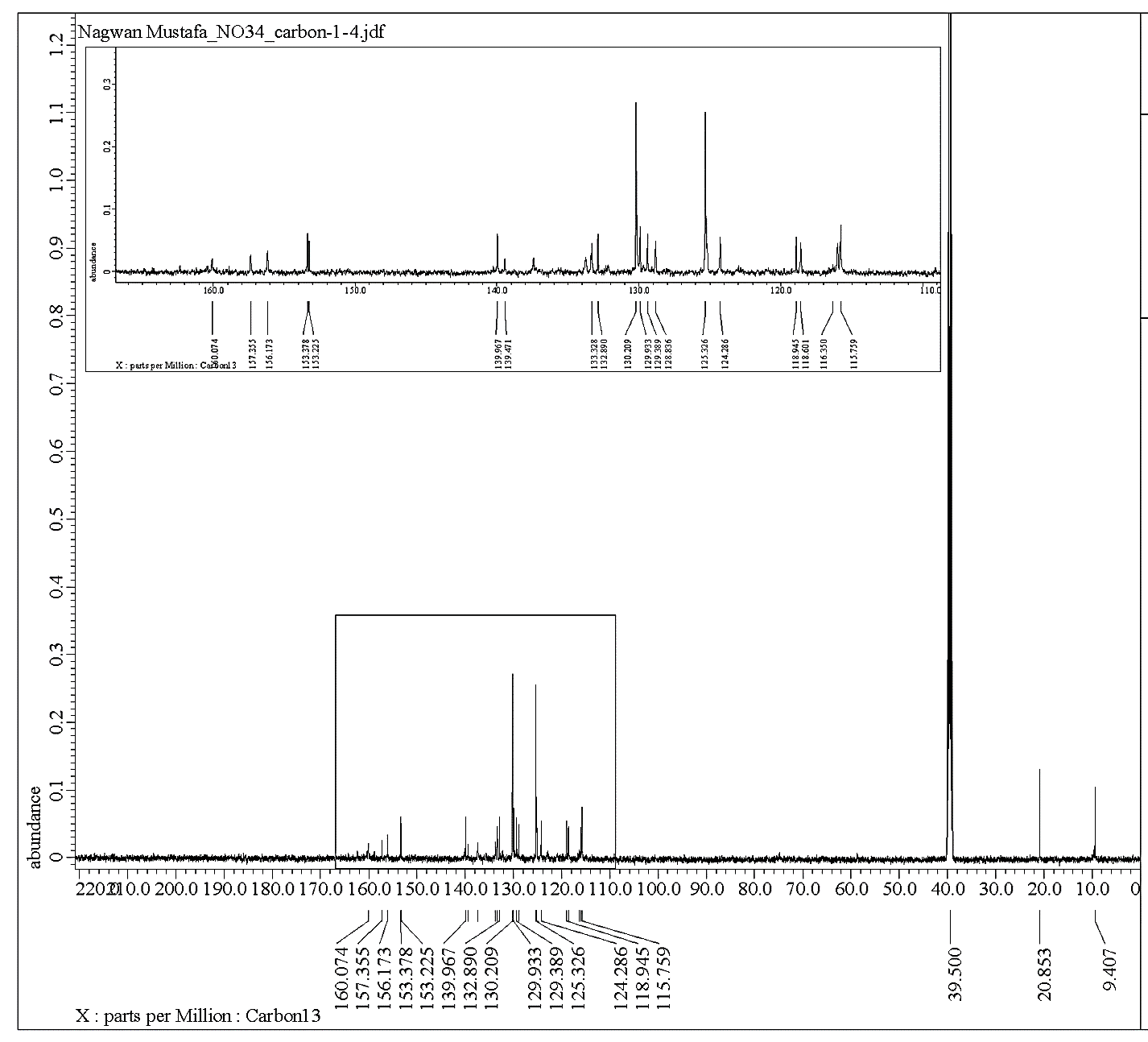

**Fig (S46): ^13^C NMR spectrum of compound NM-11.**

**Fig (S47): Mass spectroscopy of compound NM-11.**
